# Effect of financial voucher incentives provided with UK stop smoking services on the cessation of smoking in pregnant women (CPIT III): pragmatic, multicentre, single blinded, phase 3, randomised controlled trial 

**DOI:** 10.1136/bmj-2022-071522

**Published:** 2022-10-19

**Authors:** David Tappin, Lesley Sinclair, Frank Kee, Margaret McFadden, Lyn Robinson-Smith, Alex Mitchell, Ada Keding, Judith Watson, Sinead Watson, Alison Dick, David Torgerson, Catherine Hewitt, Jennifer McKell, Pat Hoddinott, Fiona M Harris, Kathleen A Boyd, Nicola McMeekin, Michael Ussher, Linda Bauld

**Affiliations:** 1 Child Health, School of Medicine, Honorary Senior Research Fellow, University of Glasgow, UK; 2 York Trials Unit, University of York, York, UK; 3 Centre for Public Health, Queen’s University Belfast, Belfast, UK; 4 National Health Service Lanarkshire Clinical Trials Unit, Airdrie, UK; 5 Health Sciences, University of York, York, UK; 6 Institute for Social Marketing and Health, University of Stirling, Stirling, UK; 7 Nursing, Midwifery and Allied Health Professions Research Unit, University of Stirling, Stirling, UK; 8 School of Health and Life Sciences, University of the West of Scotland, Paisley, UK; 9 Health Economics and Health Technology Assessment, School of Health and Wellbeing, University of Glasgow, Glasgow, UK; 10 Population Health Research Institute, St George’s, University of London, London, UK; 11 Usher Institute and SPECTRUM Consortium, University of Edinburgh, Edinburgh, UK

## Abstract

**Objective:**

To examine effectiveness, cost effectiveness, generalisability, and acceptability of financial incentives for smoking cessation during pregnancy in addition to variously organised UK stop smoking services.

**Design:**

Pragmatic, multicentre, single blinded, phase 3, randomised controlled trial (Cessation in Pregnancy Incentives Trial phase 3 (CPIT III)).

**Setting:**

Seven UK stop smoking services provided in primary and secondary care facilities in Scotland, Northern Ireland, and England.

**Participants:**

944 pregnant women (age ≥16 years) who self-reported as being smokers (at least one cigarette in the past week) when asked at first maternity visit, less than 24 weeks’ gestation, and notified to the trial team by routine stop smoking services.

**Interventions:**

Participants in the control group were offered the standard stop smoking services, which includes the offer of counselling by specially trained workers using withdrawal orientated therapy and the offer of free nicotine replacement therapy. The intervention was the offer of usual support from the stop smoking services and the addition of up to £400 ($440; €455) of LoveToShop financial voucher incentives for engaging with current stop smoking services or to stop smoking, or both, during pregnancy.

**Main outcome measures:**

Self-reported smoking cessation in late pregnancy (between 34 and 38 weeks’ gestation) corroborated by saliva cotinine (and anabasine if using nicotine replacement products). Results were adjusted for age, smoking years, index of multiple deprivation, Fagerström score, before or after covid, and recruitment site. Secondary outcomes included point and continuous abstinence six months after expected date of delivery, engagement with stop smoking services, biochemically validated abstinence from smoking at four weeks after stop smoking date, birth weight of baby, cost effectiveness, generalisability documenting formats of stop smoking services, and acceptability to pregnant women and their carers.

**Results:**

From 9 January 2018 to 4 April 2020, of 4032 women screened by stop smoking services, 944 people were randomly assigned to the intervention group (n=471) or the control group (n=470). Three people asked for their data to be removed. 126 (27%) of 471 participants stopped smoking from the intervention group and 58 (12%) of 470 from the control group (adjusted odds ratio 2.78 (1.94 to 3.97) P<0.001). Serious adverse events were miscarriages and other expected pregnancy events requiring hospital admission; all serious adverse events were unrelated to the intervention. Most people who stopped smoking from both groups relapsed after their baby was born.

**Conclusions:**

The offer of up to £400 of financial voucher incentives to stop smoking during pregnancy as an addition to current UK stop smoking services is highly effective. This bolt-on intervention supports new guidance from the UK National Institute for Health and Care Excellence, which includes the addition of financial incentives to support pregnant women to stop smoking. Continuing incentives to 12 months after birth is being examined to prevent relapse.

**Trial registration:**

ISRCTN Registry ISRCTN15236311.

## Introduction

Maternal tobacco smoking is responsible for substantial ill health and death among women and their offspring including 7% of acute childhood admissions for respiratory infection, 20% of infant deaths, and 30% of babies born underweight.^1^ Women who permanently stop smoking during pregnancy will have a near normal lifespan whereas women who continue to smoke could lose up to 10 years of life.^2^ Eighty per cent of women in the UK have at least one pregnancy, which provides an opportunity to help most young women to stop smoking before their health is permanently compromised.^3^

In the UK, midwives are routinely short of time, short of appropriate training, and perceive that detailed conversations about smoking cessation can instil guilt and undermine their relationships with pregnant women.^4^ As a result, women who are pregnant and smoke are offered counselling^5^ usually by a dedicated stop smoking service signposted by midwifery services in early pregnancy. Despite lack of evidence of effectiveness during pregnancy,^6^ in the UK^7^ (but not in many other countries, including the US^8^), nicotine replacement therapy and electronic cigarettes(e-cigarettes) are thought to reduce risk by providing nicotine only and no risk from other dangerous chemicals that are released when burning tobacco. The proportion of pregnant women who smoke has reduced in the US^9^ and the UK.^10^ Women in the US have been supported by Medicaid through changes to the Affordable Care Act that target pregnant women who smoke.^11^ In Scotland, between 1995 and 2019, self-reported smoking among pregnant women reduced from 30.5% to 14.6%,^12^ with associated reductions in miscarriage (6.9% to 4.5%)^13^ and in low birthweight for gestational age (4.2% to 2.5%)^14^

Despite this progress, women who continue to smoke while pregnant might not engage with cessation services.^15^ Interventions using financial incentives, piloted in the US,^16^ ^17^ led to service developments in the UK.^18^ ^19^ The acceptability of financial incentives to change behaviour depends on: effectiveness (even a small improvement increases acceptability); the type of incentive (grocery vouchers are more acceptable than cash or vouchers for luxury items); and the target behaviour (weight management is more acceptable than smoking cessation).^20^ However, evidence is missing from large pragmatic trials of effectiveness in the UK.^21^ Consequently, we report a phase 3 randomised controlled trial to assess whether financial incentives increase smoking cessation during pregnancy when in combination with current UK stop smoking services.

## Methods

The Cessation in Pregnancy Incentives Trial phase 3 (CPIT III) is based on a successful phase 2 (CPIT II) feasibility trial undertaken in Glasgow, Scotland.^22^ CPIT II was reviewed within the BIBS study,^23^ funded by the UK National Institute for Health and Care Research, to establish a platform for financial incentives trials. BIBS highlighted acceptability and feasibility of individually randomised trial design.

### Population

Pregnant women were recruited from seven UK stop smoking services serving maternity hospitals in Scotland, Northern Ireland, and England. Births at the sites ranged from 1000 to 6000 per year. Eligible women were self-reported smokers (at least one cigarette in the past seven days), 16 years or older, less than 24 completed weeks of gestation, and English speakers for verbal telephone consent. Examination of heterogeneity of stop smoking services at the first five of seven trial sites was part of the process evaluation outlined in the trial protocol (appendix B).^24^ The cost effectiveness analysis for the National Institute for Health and Care Excellence (NICE) guideline^25^ was based on the feasibility trial CPIT II.^26^ Cost effectiveness analysis from an NHS perspective from this trial^27^ generalisable to heterogeneous UK stop smoking services is presented in an accompanying paper preprint.^28^

### Trial design and interventions 

The trial protocol is available in appendix A.^24^ CPIT III was a pragmatic, multicentre, parallel group, single blinded, individually randomised, controlled trial. Posters and a shortened version of the patient information sheet were placed in antenatal clinic waiting rooms and information packs. Routinely collected self-reports of current smoking prompted automatic referral to stop smoking services. Information about the trial was given during the first stop smoking service contact: “You may also be suitable to take part in a study that we are currently running. The study wants to find out if giving pregnant women an incentive will help them to stop smoking. You could potentially obtain up to £400 in high street shopping vouchers if you stop smoking with our service.” Verbal permission was taken for personal details to be passed to the trial team who dispatched a patient information sheet. Trained staff at a telephone call centre within a database management company enrolled participants. Baseline and consent questions required database entry before automated randomised group allocation, ensuring concealment. Non-stratified randomisation (1:1 allocation) used randomly permuted blocks of varying size (four, six, and eight). Randomisation sequence was computer generated by the York Trials Unit and stored in a specially designed, secure, online programme for data collection.^22^ ^24^

To aid recruitment, a new region in England with five separate sites was added with enrolment starting in December 2018. The trial enrolment period was extended to achieve the calculated primary outcome sample size resulting in 25% of participants not reaching the secondary outcome six months post partum by database closure.

Control participants were offered usual stop smoking service care based on NICE guidelines^21^ which includes withdrawal orientated therapy^5^ and the offer of nicotine replacement therapy.^6^ Care varied by site in terms of general population or targeted, smoking only or more general health promotion, where care took place, who provided care, what was provided, who funded care (unpublished) and summarised in appendix B. The trial team liaised with stop smoking staff either directly or via stop smoking service notes to verify participant engagement including setting a stop smoking date, smoking status after four weeks, and carbon monoxide breath test result. Trial staff entered available carbon monoxide results into the database. Trial staff did not contact control participants before the late pregnancy primary outcome point.

Intervention participants were offered the addition of up to £400 ($540, €480) at four timepoints. Firstly, a £50 voucher to engage with stop smoking services and set a stop smoking date (face to face before start of covid (14 March 2020), then usually telephone). Secondly, if a stop smoking date was set, a £50 voucher if not smoking after four weeks confirmed by carbon monoxide (after start of covid self-report by telephone was accepted rather than carbon monoxide which required face-to-face consultation and was stopped by UK stop smoking services); thirdly, if abstinent at four weeks, a £100 voucher if smoke-free after 12 weeks confirmed by carbon monoxide. Fourthly, for all intervention participants, a £200 voucher if carbon monoxide verified smokefree, when the call centre phoned in late pregnancy at a random date between 34 and 38 weeks’ gestation (calculated from start of last menstrual period). Incentives were LoveToShop shopping vouchers redeemable in many retail outlets. If carbon monoxide results were not available for intervention participants who self-reported stopping smoking at four and 12 weeks from stop smoking services, trial staff arranged tests usually by home visit. Trial staff entered the carbon monoxide result into the database, which triggered the incentives vouchers to be dispatched by registered post from a fulfilment house. This intervention was bolted on to current services (ie, current services were changed as little as possible).

Call centre staff made multiple attempts to contact all participants in late pregnancy to establish self-report of smoking. If unsuccessful, local trial teams took over collection of primary outcome data. Some participants had delivered their babies early or had miscarried much earlier. Call centre staff attempted to contact all participants again six months after the expected date of delivery supported by local trial teams.

For all participants who self-reported as smoke-free, in late pregnancy and six months after the expected date of delivery, trial staff arranged a carbon monoxide test. If negative, a saliva sample was taken to biochemically verify self-report. To minimise loss to follow-up, women in both groups received shopping vouchers for providing late pregnancy (£50) and six months post partum (£25) outcome data including saliva for biochemical verification if abstinent.

### Outcomes and data collection

The primary outcome was verified by cotinine measured in saliva and, if required, anabasine for self-reported smoking abstinence for at least eight weeks in late pregnancy (34-38 weeks’ gestation). Self-report results were usually obtained by the call centre. At the same contact, other data were collected including current use of nicotine replacement therapy or e-cigarettes, or both.

If the participant reported that they were still smoking or had smoked in the past eight weeks, then this outcome was accepted as true and documented on the trial database. For participants recruited early in the trial and who completed the primary outcome follow-up before covid-19, if the participant reported abstinence, call centre staff made appointments with research staff using the online database to verify this abstinence. Staff visited the participant’s home to collect a carbon monoxide breath test. If negative a saliva sample was collected at the same visit. If participants were persistently not available to provide a carbon monoxide breath test, they were assumed to be smoking. The final arbiter for cessation was biochemical examination of the saliva sample. For participants recruited later in the study, appendix D describes the changes that were agreed by the ethics committee to cope with covid-19, where carbon monoxide tests and direct contact were stopped. Saliva samples for self-reported abstinence were taken by participants themselves with telephone support from trial staff. Receipt of saliva samples by trial staff prompted incentive voucher dispatch.

Cotinine is produced by the liver from nicotine from burning tobacco, nicotine patches, or inhaled nicotine, eg, e-cigarettes.^7^ Anabasine is a metabolic by product of burning tobacco and not produced from nicotine patches or e-cigarettes sold in the UK. Both can be measured in saliva. Women were biochemically verified as not smoking if their saliva cotinine concentration was <10 ng/mL,^29^ or where current nicotine replacement therapy or e-cigarette use was reported, saliva cotinine concentration was ≥10 ng/mL and saliva anabasine was ≤0.2 ng/mL.^30^

We had seven key secondary outcomes:^24^ (1) proportion of women who engaged with stop smoking services (either arrived at a face-to-face appointment or were available for the appointment by telephone and set a stop smoking date); (2) proportion of women with biochemically validated (carbon monoxide) self-reported abstinence from smoking for at least 14 days at four weeks after quit date; (3) proportion of women who self-reported point abstinence from smoking for at least eight weeks at six months post partum, verified by cotinine or anabasine tests (using the same cotinine and anabasine cut-off thresholds as the primary outcome); (4) proportion of women with cotinine and anabasine verified (using the same cotinine/anabasine cut-off thresholds as the primary outcome) self-reported continuous abstinence from smoking from late pregnancy to six months post partum; (5) mean difference in birth weight; (6) cost effectiveness^27^ presented in a linked preprint^28^; and (7) process evaluation,^24^ which provided information for heterogeneity of service formats at the first five of seven sites summarised in appendix B (unpublished).

Additionally, severity of prematurity (calculated from last menstrual period to date of birth) was collected as a proxy for length of neonatal stay, prespecified in the statistical analysis plan (appendix C). A small number of residual blood samples routinely taken for other purposes in late pregnancy were assayed for cotinine to assess if women lost to follow-up were still smoking or using nicotine replacement products.

### Trial oversight

The trial was conducted within Good Clinical Practice guidelines and ethical principles with the protocol^24^ (appendix A) approved by West of Scotland Research Ethics Committee 4. Participants provided audio-recorded informed consent obtained by specially trained call centre operators who were blind to random allocation. Data were added to the trial database by trained researchers using a secure internet portal.

Data monitoring coordinated by York Trials Unit (appendix E) was undertaken by local researchers. From March 2020, less intensive data monitoring focused on the primary outcome and key secondary outcomes, consistent with covid-19 guidance from the National Institute for Health and Care Research^31^ (see study and data monitoring plan, appendix E). Serious adverse events were reviewed by the chief investigator and were overseen by the trial steering committee.

### Statistical analysis

The planned sample size was 940 participants (470 per trial group). This number gave 90% power at 5% significance with 15% attrition to detect a clinically significant doubling of smoking cessation from 7% with usual care.

Analyses were carried out in accordance with a prespecified statistical analysis plan (appendix C) using Stata (StataCorp, release 17; College Station, TX). Statistical hypothesis tests were two sided, with a significance level of 5%. The intention-to-treat population was defined as all participants randomly assigned to the study who did not ask for all of their data to be removed and includes women who were no longer pregnant at the primary outcome data collection point.

Baseline data were summarised descriptively by treatment group for all randomly assigned participants, and for participants with primary outcome data.^32^ For each outcome, the number of participants who provided data was presented. For analysis of primary and each of the secondary outcomes relating to smoking, that is, biochemically and carbon monoxide verified smoking status, participants were assumed to be smokers (as per the Russell Standard)^33^ where the outcome was missing. Analysis of the primary outcome used a mixed-effects logistic regression model with randomised treatment group, age, smoking years, index of multiple deprivation group (divided by quintile),^34^ Fagerström score,^35^ and whether the outcome was obtained before 16 March 2020 (start of covid-19 lockdown in the UK) as fixed effects. Recruiting site was adjusted for as a random effect. The primary outcome for CPIT III was pooled with the identical outcome from the CPIT II Glasgow feasibility trial,^22^ using a random effects meta-analysis (appendix F) to obtain a pooled risk ratio.

To assess sparse data impact,^36^ the primary outcome was analysed using a Firth logistic regression model, adjusting for the same covariates as the primary analysis, with site as a fixed effect. The sensitivity to missing data was assessed using two methods, multiple imputation by chained equations and a pattern-mixture model to assess the sensitivity to deviations from the missing at random assumption.

To respond to reviewers’ concerns with regards to anabasine testing, we undertook a post hoc, worst case sensitivity analysis. This test repeated the primary analysis under the assumption that participants requiring anabasine testing in the incentives group smoked, while assuming participants who required anabasine testing in the control group did not smoke.

For each of the following subgroups, the primary analysis was repeated with addition of an interaction term between randomised treatment group and subgroup: maternal age (≤28 years *v* >28 years), index of multiple deprivation group (1st *v* 2nd *v* 3rd *v* 4th *v* 5th), years of smoking (≤10 years *v* >10 years), and Fagerstrom score (≤6 *v* >6). Subgroup analyses were prespecified in the statistical analysis plan.

We analysed engagement with stop smoking services, four week carbon monoxide validated smoking status, and continuous and point abstinence at six months post-partum using a logistic regression model, adjusting for the same fixed and random effects as the primary outcome. Birth weight analysis used mixed effects linear regression, including treatment group, age, height and weight of the mother at booking, years of smoking, income status, Fagerström score, and data collection before 16 March 2020, as fixed effects and site as a random effect. An average causal effect^37^ analysis for participants who adhered to their treatment allocation used an instrumental variable approach (appendix F) to explore intervention effects on birth weight accounting for non-adherence. Severity of preterm birth was summarised descriptively.

Covid-19 disrupted some trial processes. Primary and secondary outcomes were summarised descriptively by treatment group and before or after covid data collection timing for people who self-reported as not smoking, self-reported non-smokers with a biochemical sample, biochemically verified non-smokers, and the number of returned biochemical samples (reported in appendix D).

### Patient and public involvement

Trial planning included two people who smoked from the Glasgow feasibility trial^22^ and the UK Centre for Tobacco and Alcohol Studies smokers’ panel with additional representation on the Trial Steering Committee.

## Results

**Fig 1:**
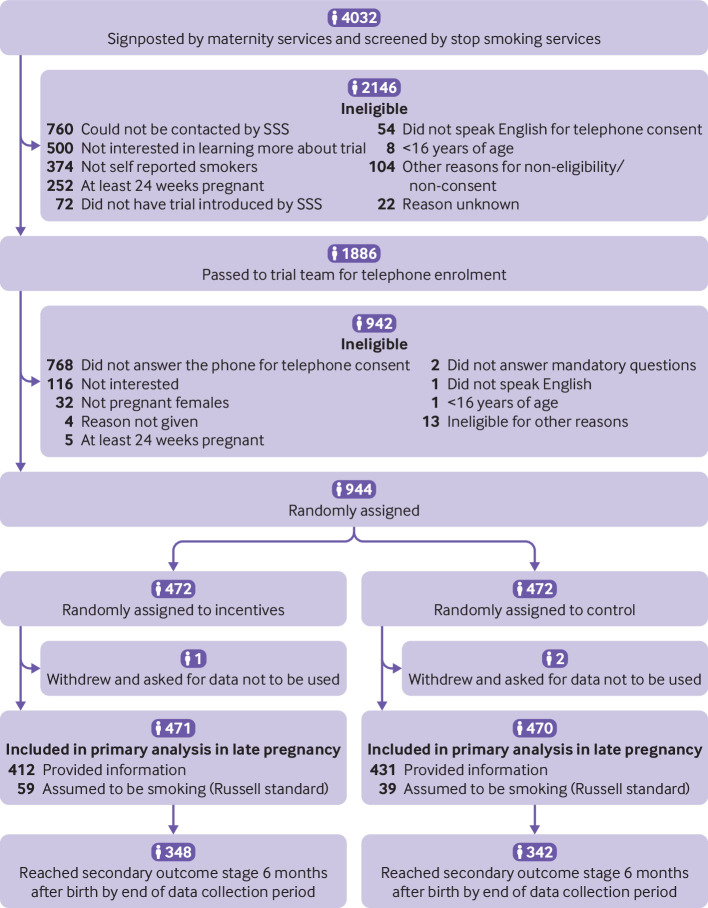
Trial profile of potential participants, and participants who were enrolled and randomly assigned to a group. SSS=stop smoking service

From 9 January 2018 to 4 April 2020, 4032 women were screened, of whom 3088 (76.6%) were either not eligible or declined consent (fig 1). As a result, 944 participants were recruited (incentives n=472; control n=472) from seven sites (n=468, 267, 77, 43, 34, 28, 27). Mean age for all participants at randomisation was 27.9 years (standard deviation 5.8) and mean gestational age at maternity booking was 11.3 weeks (standard deviation 3.3; table 1). 351 (37.2%) participants reported using nicotine replacement therapy and 171 (18.1%) reported using e-cigarettes (appendix G). Baseline characteristics were similar between randomised groups. Of 22 participants who withdrew from the study (incentives n=12; control n=10), 20 withdrew at or before the late pregnancy follow-up, whereas two withdrew at the postpartum follow-up. Seven withdrew because of a miscarriage or stillbirth (incentives n=5; control n=2), with one termination of pregnancy in the incentives group. Other reasons were: one participant not being allocated to intervention group, one participant in the control group not agreeing with using incentives to quit, one participant in the incentives group seeking support more locally, doctor asked for withdrawal for one participant in the incentive group, and 10 did not wish to continue in the trial (incentives n=4; control n=6).

**Table 1:** Baseline characteristics of pregnant smokers using stop smoking services in the trial, by study group for randomised and analysed populations. Data are number (%) of participants unless otherwise stated

Characteristic	All randomised participants* (n=944)			Participants who provided smoking status at the primary outcome stage (n=843)	
	Financial incentives (n=472)	Control (n=472)		Financial incentives (n=412)	Control (n=431)
Body mass index:					
No (%) of participants	434 (91.9)	449 (94.5)		381 (92.5)	412 (95.6)
Mean (standard deviation)	27.44 (6.35)	26.76 (6.18)		27.44 (6.32)	26.85 (6.23)
Ethnic group:					
White	466 (98.7)	464 (98.3)		407 (98.8)	426 (98.8)
Mixed/multiple ethnic groups	3 (0.6)	3 (0.6)		3 (0.7)	3 (0.7)
African/Caribbean/Black	1 (0.2)	1 (0.2)		1 (0.2)	1 (0.2)
Asian/Asian British	1 (0.2)	1 (0.2)		1 (0.2)	0 (0)
Missing	1 (0.2)	3 (0.6)		0 (0)	1 (0.2)
Maternal age at randomisation (years):					
No (%) of participants	471 (99.8)	470 (99.6)		412 (100)	431 (100)
Mean (standard deviation)	27.92 (5.71)	27.89 (5.86)		28.06 (5.60)	28.00 (5.91)
Previous live births:					
No (%) of participants	448 (94.9)	454 (96.2)		406 (98.5)	426 (98.8)
Mean (standard deviation)	2.1 (1.3)	2.2 (1.3)		2.1 (1.3)	2.2 (1.4)
Gestational age at maternity booking, weeks:					
No (%) of participants	446 (94.5)	440 (93.2)		393 (95.4)	403 (93.5)
Mean (standard deviation)	11.3 (3.3)	11.3 (3.2)		11.3 (3.3)	11.2 (3.3)
Index of multiple deprivation (divided by quintile:					
Group 1 (most deprived)	203 (43.0)	199 (42.2)		174 (42.2)	181 (42.0)
Group 2	132 (28.0)	131 (27.8)		120 (29.1)	125 (29.0)
Group 3	74 (15.7)	71 (15.0)		62 (15.0)	64 (14.8)
Group 4	33 (7.0)	32 (6.8)		30 (7.3)	25 (5.8)
Group 5 (least deprived)	15 (3.2)	19 (4.0)		12 (2.9)	19 (4.4)
Missing	15 (3.2)	20 (4.2)		14 (3.4)	17 (3.9)
Carbon monoxide reading at maternity booking (ppm):					
No (%) of participants	323 (68.4)	291 (61.7)		281 (68.2)	257 (59.6)
Mean (standard deviation)	9.7 (7.3)	9.6 (6.7)		9.7 (7.2)	9.6 (6.6)
Fagerström score:					
No (%) of participants	454 (96.2)	446 (94.5)		396 (96.1)	409 (94.9)
Mean (standard deviation)	4.1 (2.1)	4.0 (2.2)		4.1 (2.1)	4.0 (2.2)
No of cigarettes smoked a day at enrolment:					
≤10	282 (59.7)	279 (59.1)		241 (58.5)	251 (58.2)
11-20	165 (35.0)	168 (35.6)		149 (36.2)	157 (36.4)
21-30	22 (4.7)	21 (4.4)		20 (4.9)	21 (4.9)
≥31	2 (0.4)	2 (0.4)		2 (0.5)	2 (0.5)
Missing	1 (0.2)	2 (0.4)		0 (0)	0 (0)
Partner smokes:					
Yes	286 (60.6)	271 (57.4)		248 (60.2)	249 (57.8)
No	177 (37.5)	190 (40.3)		157 (38.1)	173 (40.1)
Missing	9 (2.1)	11 (2.3)		7 (1.7)	9 (2.1)
Age when participant started smoking (years):					
No (%) of participants	471 (99.8)	470 (99.6)		412 (100)	431 (100)
Mean (standard deviation)	14.7 (3.0)	14.8 (2.8)		14.7 (3.0)	14.8 (2.8)
Uses nicotine replacement therapy:					
Yes	175 (37.1)	176 (37.3)		161 (39.1)	164 (38.1)
No	296 (62.7)	294 (62.3)		251 (60.9)	267 (61.9)
Missing	1 (0.2)	2 (0.4)		0 (0)	0 (0)
Uses electronic cigarettes:					
Yes	88 (18.6)	83 (17.6)		79 (19.2)	74 (17.2)
No	383 (81.1)	387 (82.0)		333 (80.8)	357 (82.8)
Missing	1 (0.2)	2 (0.4)		0	0

Ppm=parts per million.

* Three participants who requested their data be removed from the trial database after withdrawing are included in the denominator when reporting the percentage of non-missing data (eg, the missing data for cigarettes smoked a day at enrolment is from the three participants who requested their data be removed after withdrawing).

Three participants who withdrew from the trial also asked for their data to be removed from the trial database (incentives n=1; control n=2). These participants were excluded from the analysis of the primary and secondary outcomes, along with the sensitivity analyses. Of the 690 participants who had or would have had their postpartum follow-up initiated at the planned time, 12 withdrew from the study (incentives n=6; control n=6).

### Primary outcome

Participants reached the primary outcome between 31 May 2018 and 11 September 2020 at a mean gestation of 36.0 weeks (standard deviation 1.2 weeks). A total of 843 (89.3%) women provided a self-report and biochemical verification of smoking status (412 (87.3%) in incentive group, 431 (91.3%) in control group). A significant difference was noted in biochemically verified non-smokers (126/471 (26.8%)) who were offered incentives versus participants in the control group (58/470 (12.3%); adjusted odds ratio 2.78 (95% confidence interval 1.94 to 3.97; P<0.001)). Table 2 gives information on the primary outcome analysis and the derivation of the primary outcome.

**Table 2:** Primary outcome derivation and primary analysis in pregnant smokers using stop smoking services in the trial, by study group. Data are number (%) of participants

Primary outcome	Financial incentives	Control
**Self-reported smoking status **		
Non-smoker	169/472 (35.8)	87/472 (18.4)
Smoker	267/472 (56.6)	360/472 (76.3)
Missing self-report (assumed to be smoking):	36/472 (7.6)	25/472 (5.3)
No contact	25/36 (69.4)	16/25 (64.0)
Withdrew*	11/36 (30.6)	9/25 (36.0)
**Saliva test changed outcome from non-smoker to smoker **		
Yes	19/169 (11.2)	13/87 (14.9)
No	126/169 (74.6)	58/87 (66.7)
Multiple appointments missed for saliva test (assumed to be smoking)	24/169 (14.2)	16/87 (18.4)
**Biochemically verified smoking status (primary analysis)†**		
Non-smoker	126/471 (26.8)	58/470 (12.3)
Smoker	345/471 (73.2)	412/470 (87.7)

* Includes one case and two controls who asked for their data to be removed.

† Adjusted odds ratio 2.78 (95% confidence interval 1.94 to 3.97); P<0.001.

The findings did not change by use of Firth logistic regression (adjusted odds ratio 2.72 (95% confidence interval 1.91 to 3.88); P<0.001) and by multiple imputation of chained equations (3.03 (2.10 to 4.36); P<0.001). Pattern mixture modelling showed interpretation was robust to large deviations from the missing at random assumption (appendix H).

Thirty one participants at the late pregnancy primary outcome had an anabasine test result (incentives n=22; control n=9). Assuming that the 22 participants in the incentive group were smokers and the nine in the control group were not smokers, a large treatment effect remained favouring the incentive group (adjusted odds ratio 2.17 (95% confidence interval 1.51 to 3.12); P<0.001). Nicotine replacement therapy and e-cigarette use is reported in appendix G. No significant interaction was reported between treatment allocation and prespecified subgroups.

Although the Russell standard^33^ designated people missing as smokers, of 25 participants in the incentives group who were lost to follow-up ( ‘No contact’ - table 2), six had an available residual late pregnancy blood sample and five were cotinine verified smokers or using nicotine replacement therapy or e-cigarettes. Similarly, of 16 controls lost, four had samples and three were smokers or using nicotine replacement therapy or e-cigarettes.

A random-effects meta-analysis of CPIT III with CPIT II found a pooled risk ratio of being a biochemically verified non-smoker towards the end of pregnancy of 2.30 (95% confidence interval 1.82 to 2.91; P<0.001).

### Secondary outcomes

Secondary and exploratory outcomes are summarised in table 3. Smoking status six months post partum was collected between 4 January 2019 and 13 April 2021. Significantly more engagement with stop smoking services and carbon monoxide verified non-smoking at four weeks after stop smoking date was seen with incentives (P<0.001). Data were collected for 493 (52%) participants who engaged with a stop smoking service at four weeks after a stop smoking date was obtained (302 (64.0%) of 472 participants recruited in the incentives group; 191 (40.5%) of 472 in the control group).

**Table 3:** Secondary outcome analyses in pregnant smokers using stop smoking services in the trial, by study group. Data are number or number/total number (%) of participants unless stated otherwise

Secondary outcome	Financial incentives			Control					P value
	No with data*	No/total No (% non-smokers)		No with data	No/total (% non-smokers)		Adjusted odds ratio (95% CI)		
Engaged with service and set stop smoking date	469	335/469 (71.4)		470	301/470 (64.0)		1.42 (1.06 to 1.92)		0.02
Carbon monoxide verified non-smoker at four weeks after stop smoking date	302	162/471 (34.4)		191	62/470 (13.2)		4.11 (2.85 to 5.92)		<0.001
Biochemically verified point as not smoking at six months postpartum	267	21/348 (6.0)		259	15/342 (4.4)		1.39 (0.69 to 2.79)		0.36
Biochemically verified continuous as not smoking at six months post partum	267	20/348 (5.7)		259	15/342 (4.4)		1.32 (0.65 to 2.67)		0.44
Preterm birth (exploratory)	446	—		453	—		—		—
Term (≥37 weeks)	—	406/446 (91.0)		—	423/453 (93.4)		NA		NA
Preterm (≥32 to <37 weeks)	—	33/446 (7.4)		—	26/453 (5.7)		NA		NA
Very preterm (≥28 to <32 weeks)	—	5/446 (1.1)		—	3/453 (0.7)		NA		NA
Extreme preterm (<28 weeks)	—	2/446 (0.4)		—	1/453 (0.2)		NA		NA

CI=confidence interval. NA=not calculated because statistical analysis plan indicated a descriptive summary only.

* Number of participants with data indicates the number where the research team could collect the data from staff at stop smoking services and from patient notes. The data are almost complete from those who engaged with stop smoking services but the data are less complete in some instances because the Russell standard had been adopted and participants without data were assumed to be smokers. 6 months post partum, the denominator for non-smokers is the number of participants who had reached that stage by the end of data collection.

Trial recruitment went on longer than expected owing to delay in permissions, slow recruitment, trial database and call centre relocation, and covid-19. This extension did not affect the primary outcome; however, 690 (73.3%) of 941 women (348 (73.9%) of 471 in the incentive group, 342 (72.8%) of 470 in the control group) could be followed up to six months post partum within the trial funding period. Data for biochemically verified smoking status were obtained for 526 (76.2%) of 690 participants (267 (76.7%) in the incentive group; 259 (75.7%) in the control group) with no significant difference between groups in biochemically verified non-smokers (table 3; adjusted odds ratio 1.39 (95% confidence interval 0.69 to 2.79); P=0.36).

Birth weight of babies from 443 (94%) of 471 intervention participants (mean 3.18 kg (standard deviation 0.61)) and 450 (96%) of 470 control participants (3.13 kg (standard deviation 0.60)) showed no difference between groups (mean difference 0.05 kg (95% confidence interval −0.03 to 0.13, P=0.21)). An average causal effect analysis reported a clinically important, but not significant, difference in the subset of participants who adhered with their treatment allocation (adjusted mean difference 0.31 kg (a 10% birthweight increase); 95% confidence interval −0.18 to 0.80 kg; P=0.22). Severity of preterm birth was similar between groups (table 3). Six participants for whom data for timing of birth were available did not have birth data, such as birth weight, available.

Fifty eight participants (incentives n=39; control n=19) had 61 serious adverse events (n=42; n=19). These events included: 17 miscarriages (n=12; n=5), four stillbirths (n=2; n=2), five terminations of pregnancy (n=4; n=1) of which two were for birth defects (both incentives), three neonatal deaths (n=2; n=1), one birth defect in the incentives group, one participant in the incentives group died from a drug overdose, one premature birth (requiring hospital admission or prolonging hospital admission) in the incentives group, and five admissions related to covid-19 (n=4; n=1). Twenty four other events required hospital admission: 17 for reduced fetal movements (n=11; n=6), one hyperemesis (incentives), one deep vein thrombosis (incentives), one tooth abscess (control), one for urine monitoring (control), one abdominal pain (incentives), one per vaginal bleeding (control), and one back pain and fever (incentives). All serious adverse events were considered to be unrelated to the intervention. Detection bias in the collection of adverse event data could be present owing to the nature of the intervention that meant participants randomly assigned to incentives had more contact with the trial team, and that these participants had more opportunities to report adverse events than the control group. Appendix B summarises heterogeneity of stop smoking service formats at trial sites.

## Discussion

### Principal findings

Offering up to an additional £400 of financial incentives to engage with UK stop smoking services, or to stop smoking, or both, during pregnancy increased biochemically validated stop smoking rates from 12% to 27% towards the end of pregnancy (odds ratio 2.78 (95% confidence interval 1.94 to 3.97); P<0.001). Meta-analysis with the feasibility trial^22^ that used the same bolt-on intervention in Glasgow provided a risk ratio of 2.30 (95% confidence interval 1.82 to 2.91; P<0.001).

### Strengths and limitations of this study

Contamination was explored in the current trial process evaluation by asking control group participants, in interviews, how they felt about their allocation. Although some indicated disappointment, none said this allocation had put them off stopping smoking, and these findings will be reported with supporting quotations in a future process evaluation paper. No evidence from interviews suggested that control participants felt any resentment towards participants in the incentive group. The pragmatic nature of the incentive approach—bolting on financial incentives to heterogeneous stop smoking service formats from three UK countries (Scotland, England, and Northern Ireland; appendix B)—did not disrupt current services and supports generalisability.

Reliability of anabasine test analysis, used to identify people who did not smoke but used nicotine replacement products, has been questioned. A sensitivity analysis for a worst case scenario indicates that a strong effect from incentives on smoking cessation remains (adjusted odds ratio 2.17 (95% confidence interval 1.51 to 3.12; P<0.001)).

A potential trial weakness relates to enrolment of only 944 (23%) of 4032 self-reported smokers: participants needed to agree for their contact details to be given by a stop smoking service to the trial team. Screening by stop smoking services (fig 1) reduced the population of pregnant smokers for recruitment by 50% from more than 4000 to fewer than 2000. Nearly 800 were not available for contact by stop smoking services (a common service difficulty), which could have resulted in bias towards smoking cessation, potentially raising rates of stopping smoking in both groups but not relative stop smoking rates. Smoking abstinence rates among control participants was 12% compared with the 7% used to calculate trial sample size. Of note, the number recruited from women screened (23%, 944/4032) was higher than two recent smoking cessation in pregnancy trials in which only 10% of those screened were enrolled.^38^ ^39^ Furthermore, almost all participants were white. 

### Comparisons with other studies

Unlike other trials, acceptability of financial incentives to pregnant women and health professionals from recorded interviews^24^ was examined along with cost effectiveness.^27^ ^28^ Two contemporary US trials^40^ ^41^ showed similar cessation improvements when financial incentives were added to Best Practice defined by the Centres for Disease Control. The first also examined cost effectiveness.^42^ A multicentre French study offered similar value incentives with improvement in abstinence from 7% to 16% with incentives (odds ratio 2.45, 95% confidence interval 1.34 to 4.49).^43^ Although the intervention took place in 18 maternity wards with different professionals providing support, the French incentives were closely integrated with a single cessation support design. This design would make rolling it out difficult without substantial changes to the heterogeneous UK stop smoking service formats. A phase 4 study completed in a real life setting in Glasgow, Scotland,^44^ showed that the bolt-on format of financial incentives in the current trial can be integrated into normal care and remain cost effective even with reduced incentive value.

### Policy implications

This study programme, including the current trial, the feasibility trial,^22^ and the phase 4 study in Glasgow,^44^ have shown that financial incentives more than double the smoking cessation rate and can be integrated without substantially changing current UK stop smoking services. The effectiveness should increase acceptability of using financial incentives.^20^ The variety of usual care formats for stop smoking services included in this trial (summarised in appendix B and submitted for publication (unpublished)) and cost effectiveness^28^ support rolling out the approach across the UK. This trial supports implementation advocated in NICE guidelines^25^ by showing an effective, cost effective, and generalisable pragmatic bolt-on UK format for incentive payments with real life experience from the phase 4 service development in Glasgow.^44^

### Unanswered questions and future research

Many studies have shown a rapid return to smoking post partum, suggesting the use of continued incentive payments post partum to prevent relapse (subject of an ongoing trial, ISRCTN5521821).^19^ The current trial shows a clinically important^45^ ^46^ increase in birth weight among participants who adhered to their treatment allocation (0.31 kg (95% confidence interval −0.18 to 0.80 kg); 10% of birthweight), similar to the feasibility trial^37^ (0.15 kg (−0.62 to 0.80 kg); 5% of birthweight) and to application of average causal effect analysis to the French trial^43^ (0.52 kg; 15% of birthweight). A meta-analysis of these and other relevant data would allow a definitive answer to be reached regarding an important birthweight increase^45^ ^46^ among those participants who quit smoking with the offer of financial incentives but would not have quit without that offer. Future research should look into what format and incentive level at what frequency achieves the most effective and cost effective outcome. Further trials, or well planned (phase 4) service evaluations using routinely collected stop smoking service data,^18^ ^44^ ^47^ ^48^ can now refine incentive formats for maximum effect at least cost.

**What is already known on this topic**

The proportion of women who smoke during pregnancy has halved over the past 20 years in the UK
Women who continue with smoking during pregnancy are more difficult to reach
The offer of financial incentives increases smoking cessation among pregnant women

**What this study adds**

An effective incentives framework does not disrupt current heterogeneous services to help women stop smoking during pregnancy in the UK
Adding financial incentives to current cessation support for pregnant women can save costs to the NHS in the longer term

## Data Availability

Limited data will be made available on reasonable request to York Trials Unit alex.mitchell@york.ac.uk.

## Web extra

Extra material supplied by authors

Web appendix: Supplementary material

## Notes

Contributors: DTa was the chief investigator who designed the study, wrote all the funding applications, wrote the original protocol and applied for ethics approval, oversaw and line managed Glasgow based research staff, managed all the funding, provided reports to grant funders, solved problems to do with covid, adding five English sites, as well as difficulties with ECHO moving the call centre from Bristol to Walsall, wrote the current paper. DTa with LS, MM, and LR-S worked with ECHO Managed Services to design all aspects of the data collection programme and problem solved making adjustments via ECHO database builders as problems occurred. LS was the main Trial Manager who controlled all the day to day running of the trial, problem solved all aspects of trial delivery, at seven sites including travelling to six sites with DTa and MM to support local researchers, ran the seventh site with MM, wrote the protocol publication and reviewed the current paper drafts. LS managed the trial administrator based in Glasgow. FK line managed research staff at one site, supported trial design, led application for funding at the site as well as additional funding for the process evaluation, provided support and advice to DTa whenever asked for, reviewed drafts of the current publication. MM was the lead research nurse for the whole trial, who supported all the research nurses at the six research sites as well as running the trial at the seventh site. MM travelled to all sites with DTa and LS solved problems, and with DTa and LS travelled to ECHO in Bristol and Walsall to train call centre staff and monitor performance and provide further advice. MM reviewed the current manuscript. LR-S took control of the ethics and NHS Research and Development approval including all protocol amendments. LR-S also supported LS with the running of the whole trial. LR-S organised the Trial Steering Committee and Trial Management Group meetings providing all the paperwork and recorded minutes. LR-S organised for all the saliva samples to be packaged and transferred to the laboratory from all seven sites. LR-S developed and put in place the Trial Data Monitoring Plan. L-RS reviewed this manuscript. AM provided the main statistical support and supported redevelopment of the trial database. AM developed the programmes to analyse the trial data. AM provided all the results for this manuscript. AM wrote the Statistical Analysis Plan, the statistical analysis section of the manuscript, and reviewed the manuscript multiple times. AK supervised all the work of AM and gave frequent advice as required. AK helped to develop the Statistical Analysis Plan and guided the analysis and statistical aspects of responses to reviewers comments. AK reviewed this manuscript. JW oversaw the clinical trial methodology of the study and supported LR-S with all aspects of trial oversight. JW reviewed this manuscript. SW and AD ran one site organising and implementing data collection and data entry into the trial database. SW also liaised locally with NHS Research and Development who provided approvals. SW and AD supported the process evaluation aspects of the trial by creating an online questionnaire with JM to augment face to face interviews in order to gather the opinions of health carers regarding the use of financial incentives to support smoking cessation during pregnancy. AD and AW supported LR-S with sample transport. DTo and CH provided clinical trial unit support for all aspects of CPIT III. They allocated time for AM, LR-S, AK, JW to work on the trial, adjusted commitment and timelines to cope with two trial extensions allowing the statistical analysis and interpretation to be documented when needed. DTo and CH both reviewed the manuscript and DTo provided comments on drafts many times. JM supervised by PH and FMH undertook the process evaluation for the CPIT III trial. JM travelled to sites and observed interactions between the central trial team and local site research nursing staff. JM interviewed face to face local research nurses, local maternity care, and stop smoking service staff as well as trial participants and pregnant smokers who did not participate. JM developed with SW and AD the online questionnaire for local staff to augment face to face interviews which were curtailed by covid 19. JM, PH, and FMH developed the analysis plan for the process evaluation and undertook the analysis. JM wrote the accompanying paper submitted to *BMJ Open*. JM, PH, and FMH have reviewed and provided changes to this manuscript. NM supervised by KB provided the health economic assessment for the CPIT III trial. NM travelled to sites with the central trial team and developed the health economics data collection system with local site research staff. NM worked with AM and produced a health economics dataset. NM analysed the data and both NM and KB wrote a sister paper reporting the health economic outcomes of the CPIT III trial. NM and KB have read and provided input to drafts of this manuscript. MU has provided advice and input to the trial from the beginning of grant applications in 2015 through until the present. MU has been an active member of the trial management group; having run a trial of exercise to support pregnant smokers to quit, he has important insights into difficulties and provided solutions; he has read and reviewed many drafts of this manuscript and helped respond to reviewers comments. LB has worked with DTa on this programme of work since 2008. LB has guided grant application, removed or found a way around roadblocks and supported prolonged frustrating efforts to fund this important trial. LB has guided the trial team through difficult situations and provided support to DTa and line management to LS the trial manager. LB guided the team to gain approval for two extensions, one funded by Cancer Research UK and the Chief Scientist Office. LB has reviewed this manuscript. DTa is study guarantor. The corresponding author attests that all listed authors meet authorship criteria, that no others meeting the criteria have been omitted and acts as a guarantor.

The lead author (the manuscript’s guarantor) DTa affirms that the manuscript is an honest, accurate, and transparent account of the study being reported; that no important aspects of the study have been omitted; and that any discrepancies from the study as planned (and, if relevant, registered) have been explained.

Dissemination to participants and related patient and public communities: A lay summary has been created and sent to all participants who wished to receive it. A summary for the Chief Scientist Office, Scottish Government will be available after publication of this manuscript: https://www.cso.scot.nhs.uk/outputs/focus-on-research-summaries/focus-on-research-reproductive-health-and-childbirth.

Provenance and peer review: Not commissioned; externally peer reviewed.
