# Supplementary material for: Effect of financial voucher incentives provided with UK stop smoking services on the cessation of smoking in pregnant women (CPIT III): pragmatic, multicentre, single blinded, phase 3, randomised controlled trial
Source: BMJ. 2022 Oct 19;379:e071522. doi: 10.1136/bmj-2022-071522 (PMC9580214; doi:10.1136/bmj-2022-071522)

Appendices

Appendix A: Final protocol with summary of changes

STUDY PROTOCOL (Cover Page)

The Smoking Cessation in Pregnancy Incentives Trial (CPIT):

A Phase III Randomised Controlled Trial

V7.1 10th March 2022

CHIEF INVESTIGATORS (CIs):

Professor David M Tappin<br>Professor of Clinical Trials for Children<br>University of Glasgow<br>Scottish Cot Death Trust<br>West Glasgow Ambulatory Care Hospital<br>Dalnair Street, Yorkhill<br>Glasgow, G38SJ	Professor Linda Bauld<br>Bruce and John Usher Professor of Public Health<br>Usher Institute<br>University of Edinburgh<br>Teviot Place<br>Edinburgh, EH8 9AG
**Phone:**07784 304568	**Phone:**0131 650 3226
**Email:** david.tappin@glasgow.ac.uk	**Email:** linda.bauld@ed.ac.uk

**SPONSOR:**<br>NHS Greater Glasgow and Clyde	**SPONSORREPRESENTATIVE:**<br>Ms Joanne McGarry (2017 to October 2019)<br>Ms Emma McDonough (October 2019 to October 2021)<br>Ms Barbara Ross (October 2021 to date)<br>Research Co-ordinator<br>NHS Greater Glasgow & Clyde<br>Clinical Research & Development Central Office<br>Ward 11 Dykebar Hospital<br>Grahamstone Road<br>Paisley, PA2 7DE
	**Phone:**0141 314 4011
	**Email:** Barbara.Ross2@ggc.scot.nhs.uk

FUNDING SOURCES:
Cancer Research UK, Chief Scientist Office Scottish Government, Health and Social Care Northern Ireland, Chest Heart & Stroke Northern Ireland, Public Health Agency Northern Ireland, Lullaby Trust, Scottish Cot Death Trust

REFERENCE NUMBERS
Sponsor Project Number: GN14OB206

ISRCTN: ISRCTN15236311 (date registered 13/10/2017)

IRAS Project ID: 227489

REC Ref: 17/WS/0173. West of Scotland REC 4

NIHR Clinical Research Network Portfolio Number: 36323

Trial web site: https://www.york.ac.uk/healthsciences/research/trials/research/trials/cpit-iii/

SIGNATURE PAGE

Authorised signatories of protocol:

**Joint Chief Investigator**<br>Prof David Tappin<br>University of Glasgow	**Signature**<br>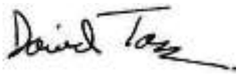	**Date**<br>**09/07/2018,**<br>**16/03/2020**
**Joint Chief Investigator**<br>Prof Linda Bauld<br>University of Stirling	**Signature**<br>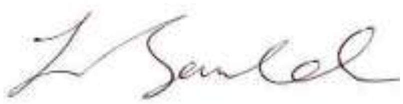	**Date**<br>**09/07/2018,**<br>**16/03/2020**
**Sponsor Representative**	**Signature**	**Date**
Ms Joanne McGarry<br>NHS Greater Glasgow & Clyde	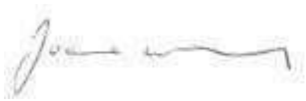	**01/06/2018**
Mrs Emma McDonough<br>NHS Greater Glasgow & Clyde	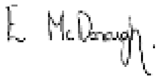	**04/08/2020**
Mrs Barbara Ross<br>NHS Greater Glasgow & Clyde	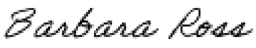	**18/03/22**

PROTOCOL AMENDMENT HISTORY

AM No.	AM Type & No.	AM Date	Purpose of Amendment
N/A	N/A	16/08/2017	Original REC submission
AM01	NSA	14/09/2017	Change of PI in NHSL from D Tappin and L Bauld to Shirley Mitchell prior to initial approval
AM02	SA01	01/11/2017	Changes to content of main consent form, main PIS and protocol. No changes to data collected, safety of participants or trial procedures
AM03	NSA	05/01/2018	Changes to PIS: Formatting and layout. Also made clear that £100 voucher at 3 months only available if quit smoking at 4 weeks<br>Change to Consent Form: Paragraph 2 - changed 'treatment' to 'support'
AM04	NSA	24/05/2018	Change to protocol regarding clarification of CO cut off and vouchers new text: 'Incentive payments at 4 and 12 weeks post-quit date (see 10.2.2 and 10.2.3) and 34-38 weeks gestation are based on the SSS accepted CO levels for a non-smoker at each site. In routine practice an element of discretion is exercised around these CO levels to allow for passive smoking. The trial will thus adhere to the same principles and consideration will be given to individual cases (agreed with sponsor).' page 30
AM05	NSA	21/06/2016	AM05 was an "administrative clarification to protocol" dated 21 June 2016 (year looks incorrect) processed by the HRA, which the REC was not informed about that is related to AM04 - Change to protocol regarding clarification of CO cut off levels for incentive payments
AM06	SA02	10/07/2018	See AM06 Full Details tab Appendix 1 for details of the amendment.
AM07	NSA	28/09/2018	GDPR compliance changes and addition of new site (Site A) (Adding a site is a NSA - see 31/07/2018 e-mails from Louise Bell)
AM08	NSA	10/01/2019	Addition of new sites
AM09	NSA	19/03/2019	Addition of new site - Site G
N/A	Administrative change included in AM11	26/07/2029	CPIT Central Office address change
AM10	NSA Dec 2019	05/12/2019	3-month funded extension

AM11	SA03	05/03/2020	Changes to timing of planned post-partum follow-up<br>Addition of sensitivity analyses<br>Change of PI at Site C from Joanna Dover to Beth Gilhooly<br>Addition of new sites in Wessex (AM0728/09/2019, NSA Jan 2019, NSA Mar 2019)<br>Inclusion of text re adherence with intervention<br>Incorporation of 3-month funded extension to timelines (NSA Dec 2019)<br>Inclusion of secondary objective/outcome measure previous omitted in error<br>Updates to trial reference numbers (cover page), protocol amendment history (p3), contact details (cover page, section 1.0), funding sources (cover page, Study synopsis), list of abbreviations and TSC to reflect all previous approved SA's and NSA's<br>Revised improved version of study visits flow chart : 2.2 Schedule Of Assessment and Data Collection for Trial Outcomes<br>CPIT Central Office address change<br>Other minor corrections to and formatting of text<br>Full details shown in separate tab/protocol
AM12	NSA	30/03/2020	Exception from suspension of GG&C hosted clinical trials due to Covid-19. Face-to-face contact between NHS research staff and participants replaced with remote contact by university research staff and participants to continue data collection.<br>Full details shown in separate tab
AM13	SA04	18/12/2020	Addition of online HCP survey for Process Evaluation
AM14	NSA	24/03/2021	Revised HP Survey & no-cost extension
AM15	NSA	03/02/2022	Timeline update to reflect further no-cost extension to 31/07/2022 and update to sponsor rep and LS contact details
AM16	NSA	10/03/2022	Minor changes to study documents

STUDY SYNOPSIS

**Lay Title**	**Quitting smoking in pregnancy with the help of shopping vouchers**
**Official Title**	The Smoking **C**essation in **P**regnancy **I**ncentives **T**rial (CPIT): A phase III randomised controlled trial
**Brief summary**	We will compare the smoking cessation rate when offering pregnant smokers financial incentives in the form of shopping vouchers, in addition to usual care, to engage with stop smoking services and/or to quit smoking. Our aim is to show that, within a range of usual care situations, the addition of financial incentives provided in a simple transferrable format, increases smoking cessation; that stopping during pregnancy leads to prolonged cessation to at least six months after birth and that the addition of financial incentives is cost effective, well below the threshold set by NICEto recommend widespread deployment of a new intervention
**Sponsor reference number**	GN14OB206
**Public database Trial identifiernumber**	ISRCTN15236311
**Study type and phase**	Non-CTIMP. Phase III
**Study design**	A randomised controlled trial
**Chief Investigators**	Professor David Tappin, University of Glasgow<br>Professor Linda Bauld, University of Edinburgh
**Study population**	Smokers routinely identified at maternity booking
**Condition**	Smokers
**Study groups**	Intervention/Control, no stratification
**Eligibility criteria**	**Inclusion criteria**<br>Self-reportedsmoker<br>>= 16 years<br>Pregnant less than 24 weeks<br>English speaking
	**Exclusion criteria**<br>Non-smoker<br><16 years<br>Pregnant > = 24 weeks<br>Non-English speaking
**Target number of participants**	940
**Interventions**	Control and intervention groups: The offer of usual NHS smoking cessation service support and shopping vouchers for £50 and £25 for providing primary outcome data and secondary outcome data respectively.<br><br>Intervention group: In addition the intervention group will receive the offer of up to an additional £400 of shopping vouchers: £50 for attending first routine face to face counselling appointments and setting a quit date, £50 if quit 4 weeks later verified by exhaled carbon monoxide (CO), £100 if CO verified quit 12 weeks post quit date, £200 if CO verified quit at 34-38 weeks gestation
**Criteriafor evaluation**	**Primary outcome measure(s)**

	Self-reported abstinence from smoking for at least eight weeks prior to 34-38 weeks gestation verified by cotinine and/or anabasine in urine/saliva
	**Secondary outcome measure(s)**<br>Engagement with smoking cessation services. Biochemically validated (CO) self-reported abstinence from smoking at four weeks after quit date. Cotinine and/or anabasine verified self-reported continuous abstinence from smoking six months after birth for participating women enrolled to the study prior to 31/12/2019. Women recruited later will be followed up at the latest point postpartum (e.g. 0 up to 6 months) until 31/10/2020 when data collection ceases. The starting point for continuous abstinence will be from birth. Birth weight, cost effectiveness (using the EQ-5D as the measure of utility) and process evaluation will also be considered
	**Other outcome measure(s) if applicable**<br>Cotinine and/or anabasine verified self-reported point abstinence up to six months after birth. Point abstinence will be defined as abstinence for at least eight weeks prior to contact
**Sources of funding**	Cancer Research UK, Chief Scientist Office Scottish Government, Health and Social Care Northern Ireland, Chest Heart and Stroke Northern Ireland, Public Health Agency Northern Ireland, Lullaby Trust, Scottish Cot Death Trust. Applications also made to National Cancer Institute, NIH USA for the London Centre and Yorkshire Cancer Research for Centre in Sheffield
**Start date:**	1 September 2017
**Anticipated finish date**	31st July 2022

TABLE OF CONTENTS

STUDY PROTOCOL (Cover Page) 1

SIGNATURE PAGE 2

PROTOCOL AMENDMENT HISTORY 3

STUDY SYNOPSIS 7

TABLE OF CONTENTS 9

LIST OF ABBREVIATIONS 12

1.0 ROLES AND RESPONSIBILITES 13

1.1 Chief investigators responsible for the study 13

1.2. Co-investigators (named funding applicants) 13

1.3 Principal Investigators for sites 14

1.4 Trial Management 15

1.4.1 Contact Details 17

2.0 STUDY SUMMARY & SCHEMA 20

2.1 Participant Flow Diagram 20

2.2 Schedule of Assessment and Data Collection for Trial Outcomes 20

2.3 Lay Summary 23

3.0 INTRODUCTION 24

3.1 Background 24

3.2 Rationale and justification for study 24

4.0 HYPOTHESIS AND OBJECTIVES 25

4.1 Hypothesis 25

4.2 Primary objective 25

4.3 Secondary objectives 25

5.0 STUDY DESIGN 26

6.0 STUDY POPULATION 27

6.1 Setting and locations 27

6.2 Number of participants to be enrolled 27

6.3 Eligibility criteria 27

6.3.1 Inclusion criteria 27

6.3.2 Exclusion criteria 27

6.4 Withdrawal criteria 27

6.5 Participant replacement 27

7.0 INTERVENTION 28

7.1 Intervention group 28

7.2 Control group........ 28

8.0 OUTCOME MEASURES........ 29

8.1 Primary outcome measure........ 29

8.2 Secondary outcome measures........ 29

9.0 STUDY TIMELINE........ 29

10.0 METHODS ANDASSESSMENTS........ 30

10.1 Recruitment........ 30

10.1.1 Initial screening and permission to pass contact details to the trial team........ 30

10.1.2 Informed consent........ 31

10.1.3 Randomisation........ 32

10.1.4 Blinding........ 32

10.2 Study visits and procedures........ 33

10.2.1 Initial face to face appointment with smoking cessation advisor (Intervention and controlgroups)........ 33

10.2.2 At 4 weeks post-quit date (intervention and controlgroups)........ 33

10.2.3 At 12 weeks (3 months) post-quit date (only intervention participants who self-report quit at 4 weeks)........ 34

10.2.4 34-38 weeks gestation (allparticipants)........ 34

10.2.5 Up to 6 months post-partum (calculated from the expected date of delivery) (all participants): cotinine validated quit........ 35

10.2.6 Discontinuation visit and procedures........ 36

11.0 ECONOMIC EVALUATION........ 37

11.1 Process and Analysis........ 37

11.2 Gaming........ 37

12.0 PROCESS EVALUATION RESEARCH........ 39

12.1 Study Design........ 39

12.1.1 Data Collection........ 40

12.1.2 Data analysis........ 42

12.2 Data Handling........ 42

12.3 Ethical issues related to the process evaluation........ 43

12.4 Payment fortaking part in interviews........ 43

13.0 SAFETY ASSESSMENTANDREPORTING........ 44

13.1 Definitions........ 44

13.2 Collecting, recording and reporting of adverse events........ 44

14.0 DATA MONITORING ANDQUALITY ASSURANCE........ 45

14.1 Audits and inspections........ 45

14.2 Data quality assurance.....45
14.3 Data entry, data management and storage.....45
15.0 STATISTICAL CONSIDERATIONS.....47
15.1 Determination of sample size.....47
15.2 Main Analysis.....47
15.2.1 Primary outcome analysis.....47
15.2.2 Secondary outcome analysis.....47
15.2.3 Other outcome analysis.....48
15.2.4 Missing data.....48
15.2.5 Sensitivity analyses.....48
15.3 Health economics analysis.....48
16.0 REGULATORY CONSIDERATIONS.....50
16.1 Ethical and research governance approval.....50
16.2 Informed consent.....50
16.3 Confidentiality of data and patient records.....51
16.4 Potential risks and benefits.....51
16.5 Indemnity.....52
17.0 TRIAL OVERSIGHT.....53
17.1 Trial Management Group (TMG).....53
17.2 Trial Steering Committee (TSC).....53
17.3 Data Monitoring Committee (DMC).....53
18.0 USER AND PUBLIC INVOLVEMENT.....54
19.0 PUBLICATIONS.....54
20.0 RETENTION OF TRIAL DOCUMENTS.....54
21.0 STUDY CLOSURE/DEFINITION OF END OF TRIAL.....55
REFERENCES.....56
Appendix 1 – Study Timelines.....58
Appendix 2 - Proposalfor CPIT III follow-up during COVID-19 pandemic.....57

LIST OF ABBREVIATIONS

CO	Carbon Monoxide
DMC	Data Monitoring Committee
GP	General Practitioner
ISF	Investigator Site File
NICE	The National Institute for Health and Care Excellence
NRT	Nicotine Replacement Therapy
PIS	Participant Information Sheet
SE	Serious Event
SAE	Serious Adverse Event
SSS	Stop Smoking Service(s)
TMF	Trial Master File
SFTP	Secure File Transfer Protocol
TMG	Trial Management Group
TSC	Trial Steering Committee

1.0 ROLES AND RESPONSIBILITES

1.1 Chief investigators responsible for the study

Professor David M Tappin<br>Professor of Clinical Trials for Children<br>University of Glasgow<br>Scottish Cot Death Trust<br>5th Floor, West Glasgow Ambulatory Care Hospital<br>Dalnair Street, Yorkhill<br>Glasgow, G38SJ	Professor Linda Bauld<br>Bruce and John Usher Professor of Public Health<br>Usher Institute<br>University of Edinburgh<br>Teviot Place<br>Edinburgh, EH8 9AG
**Phone:**07784 304568	**Phone:**0131 650 3226
**Email:** david.tappin@glasgow.ac.uk	**Email:** linda.bauld@ed.ac.uk

1.2. Co-investigators (named funding applicants)

Professor Frank Kee<br>Director of the UKCRC Centre of Excellence for Public Health (Northern Ireland)<br>Centre for Public Health<br>School of Medicine, Dentistry and Biomedical Sciences, Queen's University Site C Grosvenor Road, Site C, BT12 6BA	Professor Michael Ussher<br>Professor of Behavioural Medicine<br>Population Health Research Institute<br>6th Floor Hunter Wing<br>St Georges, University of London<br>Cranmer Terrace<br>London, SW17 ORE
**Phone:**028 9097 8943	**Phone:**020 8725 5605
**Email:** F.Kee@qub.ac.uk	**Email:** mussher@sgul.ac.uk

	Dr Kathleen Boyd<br>Senior Lecturer (Health Economics)<br>Institute of Health & Wellbeing<br>Health Economics and Health Technology Assessment<br>University of Glasgow<br>1 Lilybank Gardens<br>Glasgow, G12 8RZ
	**Phone:**0141 330 2713
	**Email:** Kathleen.Boyd@glasgow.ac.uk

Lesley Sinclair<br>Research Fellow/Trial Manager<br>York Trials Unit<br>Department of Health Sciences<br>Faculty of Science<br>ARRC Building<br>University of York<br>Heslington, York YO10 5DD	Professor Pat Hoddinott<br>Chair in Primary Care<br>NMAHP Research Unit<br>13 Scion House<br>University of Stirling<br>Stirling, FK9 4NF
**Phone:** TBC	**Phone:**01786 466341
**Email**: Lesley.sinclair@york.ac.uk	**Email:** p.m.hoddinott@stir.ac.uk

Dr Fiona Harris	Jennifer McKell

Associate Professor<br>NMAHP Research Unit<br>13 Scion House<br>University of Stirling<br>Stirling FK9 4NF	Research Fellow<br>Institute for Social Marketing<br>Faculty of Health Sciences & Sport<br>Pathfoot Building<br>University of Stirling<br>Stirling, FK9 4LA
**Phone:**01786 466184	**Phone:**01786 467351
**Email:** fiona.harris1@stir.ac.uk	**Email:** j.e.mckell@stir.ac.uk

Professor Catherine Hewitt<br>Deputy Director of York Trials Unit and Senior Statistician<br>Department of Health Sciences<br>Faculty of Science<br>University of York<br>Heslington, York, YO10 5DD	Professor David Torgerson<br>Director of York Trials Unit<br>Department of Health Sciences<br>Faculty of Science<br>University of York<br>Heslington,<br>York, YO10 5DD
**Phone:**01904 321374	**Phone:**01904 321340
**Email:** catherine.hewitt@york.ac.uk	**Email:** david.torgerson@york.ac.uk

Dr Sue Cooper<br>Principal Research Fellow and NIHR Programme Manager<br>University of Nottingham<br>Division of Primary Care, School of Medicine<br>Room 1408, Tower Building, University Park<br>Nottingham, NG72RD	Professor Tim Coleman<br>Professor of Primary Care<br>University of Nottingham<br>Division of Primary Care<br>D1411, Medical School<br>Queen's Medical Centre<br>Nottingham, NG72UH
**Phone:**0115 82 31898	**Phone:**0115 8230204
**Email:** Sue.Cooper@nottingham.ac.uk	**Email:** Tim.Coleman@nottingham.ac.uk

13 3 Principal Investigators for sites

1.4 4 Trial Management

The trial will be co-ordinated by staff based at both the trial co-ordinating centre in Glasgow and York Trials Unit (YTU). The process evaluation will be led by staff at University of Stirling, the health economic evaluation by staff at University of Glasgow and the data management and analysis by staff at YTU (Figure 1).

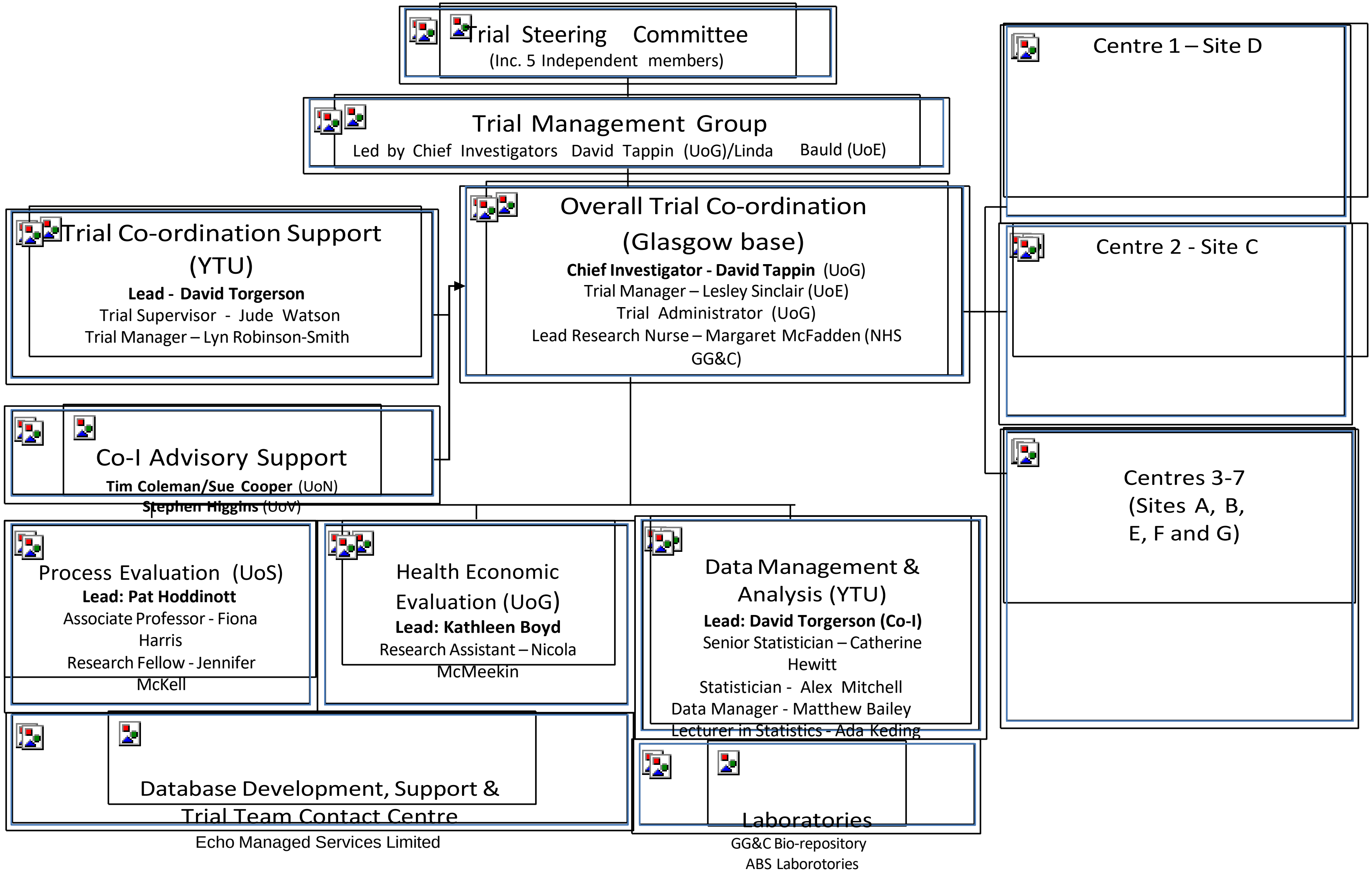

Key: GG&C = Greater Glasgow & Clyde
QUB = Queens University Site C
UoS = University of Stirling
SGUL = St George's University of London
UoV = University of Vermont
UoG = University of Glasgow
YTU = York Trials Unit
UoN = University of Nottingham
UoE = University of Edinburgh

Figure 1: Trial team configuration

1.4.1 Contact Details

GLASGOW CO-ORDINATING CENTRE:

NHS Greater Glasgow & Clyde<br>Clinical Research & Development<br>Central Office<br>Ward 11<br>Dykebar Hospital<br>Grahamstone Road<br>Paisley<br>PA2 7DE	**Principal Investigator:**<br>Professor David Tappin<br>david.tappin@glasgow.ac.uk
	**Lead Trial Nurse:**<br>Margaret McFadden<br>Margaret.McFadden@ggc.scot.nhs.uk

YORK TRIALS UNIT:

York Trials Unit<br>ARRC Building, Lower Ground Floor<br>University of York<br>Heslington<br>York<br>YO10 5DD	**Co-applicant & Director of YTU:**<br>Prof David Torgerson<br>david.torgerson@york.ac.uk
	**Senior Research Fellow:**<br>Dr Jude Watson<br>jude.watson@york.ac.uk
	**Deputy Director of YTU & Senior Statistician:** Prof Catherine Hewitt<br>Catherine.hewitt@york.ac.uk
	**Lecturer in Statistics:**<br>Dr Ada Keding<br>ada.keding@york.ac.uk
	**Statistician:**<br>Alex Mitchell<br>Alex.mitchell@york.ac.uk
	**Senior Data Manager:**<br>Matthew Bailey<br>matthew.bailey@york.ac.uk
	**Trial Manager & Research Fellow:**<br>Lyn Robinson-Smith<br>lyn.robinson-smith@york.ac.uk<br>(Helen Tilbrook helen.tilbrook@york.ac.uk<br>(until Nov-19)

CO-INVESTIGATORS ADVISORY SUPPORT

These co-investigators willnot lead a centre but provide advice and support to the study team:

**Nottingham (study advisers):**	**Vermont USA (study advisers):**
Professor Tim Coleman<br>Tim.Coleman@nottingham.ac.uk<br>Professor of Primary Care<br>University of Nottingham	Professor Stephen T. Higgins<br>Stephen.Higgins@uvm.edu<br>Director<br>Vermont Centre on Behavior and Health
Dr Sue Cooper<br>Sue.Cooper@nottingham.ac.uk<br>Principal Research Fellow<br>University of Nottingham	Professor Virginia H. Donaldson<br>Professor of Translational Science<br>Departments of Psychiatry and Psychology<br>Vice Chair, Department of Psychiatry<br>University of Vermont<br>1 South Prospect Street<br>Burlington<br>VT<br>05401-1419<br>USA

DATABASE DEVELOPMENT AND CONTACT CENTRE:

Echo Managed Services Limited<br>Green Lane<br>Walsall<br>West Midlands<br>WS2 7PD

HEALTH ECONOMIC EVALUATION:

Dr Kathleen Boyd<br>Kathleen.Boyd@glasgow.ac.uk<br>Senior Lecturer<br>Health Economics and Health Technology Assessment<br>University of Glasgow

MIXED METHODS PROCESS EVALUATION:

Professor Pat Hoddinott<br>p.m.hoddinott@stir.ac.uk<br>Chair in Primary Care<br>University of Stirling	Dr Fiona Harris<br>fiona.harris1@stir.ac.uk<br>Associate Professor<br>Qualitative Research Lead for NMAHP Research Unit<br>University of Stirling

Jennifer McKell<br>j.e.mckell@stir.ac.uk<br>Research Fellow<br>Institute for Social Marketing<br>University of Stirling	

LABORATORIES FOR CLINICAL SAMPLES:

ABS Laboratories Ltd<br>36 Hospital Fields Road<br>York<br>YO10 4DZ<br>www.abslabs.com	**Local Laboratories:**<br>Local secure storage facility at each centre
**Phone:**01904 403600	
**Email:** queries@abslabs	

TRIAL STEERING COMMITTEE:

**Chair:**<br>Dr Felix Naughton<br>F.Naughton@uea.ac.uk<br>Senior Lecturer in Health Psychology<br>University of East Anglia

STUDY SUMMARY & SCHEMA

21 1 Participant Flow Diagram

22 2 Schedule of Assessment and Data Collection for Trial Outcomes

**Data Item/Outcome**		**Definitions and measurement techniques**	**Timepoint obtained/measured**						
			**Baseline/ Consent/ Randomisation**	**Follow-up 1: SSS Engagement**	**Follow-up 2: 4 weeks post quit date**	**Follow-up 3: 12 weeks post quit date**	**Follow-up 4: Late pregnancy (34-38 weeks gestation)**	**Delivery**	**Follow-up 5: 6 months post-partum**
**Demographic/health data**									
	Age		x						
	Height		x						
	Weight		x						
	Ethnicity		x						
	English speaking		x						
	Household income	Total annual income of household before tax & deductions	x						
	Deprivation quintile	Index of Multiple Deprivation derived from postcode of residence	x						
	Quality of Life	EuroQol EQ-5D-5L	x				x		x
**SSS/Smoking related data**									

	Event dates		x	x	x	x	x	x	x
	Age first smoked		x						
	Nicotine dependence level	Fagerstrom Test for Cigarette Dependence	x						
	SSS attendance			x	x	x			
	Self-reported smoking status	Modified Russell Standard criteria	x		x	x	x		x
	CO breath test result	Expired Carbon Monoxide	x	x	x	x	x		x
	Quit date			x					
	Urine/saliva cotinine/anabasine	As per published guidance [1]					x		x
**Smoking cessation aids**									
	E-cig use	Current use/use since becoming pregnant	x				x		x
	NRT use	Current use/use since becoming pregnant	x	x			x		x
**Maternity/birth data**									
	Expected date of delivery		x						
	Actual date of delivery							x	
	Multiple birth							x	
	Birthweight							x	
	Stillbirth/Miscarriage			x	x	x	x	x	
	Parity	No. times given birth to fetus >=24 weeks gestation						x	

2.3 3 Lay Summary

BACKGROUND: Lifelong smokers lose 10 years of life. Smoking cessation by age 40 leads to a near normal lifespan. Eighty percent of women have a baby, most by age 40, making pregnancy an opportunity to help women quit before their health is irreversibly compromised. Few of the UK's current 130,000 pregnant smokers quit despite free counselling and Nicotine Replacement Therapy. Offering financial incentives for smoking cessation has worked in local single site trials including in Glasgow where the pilot study for this trial took place. NICE have asked for evidence from a multi-centre trial.

AIMS: To conduct a pivotal phase III randomised controlled multi-centre trial to examine the effectiveness and cost effectiveness of offering financial incentives in the form of shopping vouchers to pregnant smokers to engage with smoking cessation services, quit smoking during pregnancy and stay quit afterpregnancy.

METHODS: This 42 month trial will recruit 940 smokers over 18-24 months in 2-4 UK centres and follow them until 6 months after birth. The extra cost and long-term benefits will be used to calculate the cost per Quality Adjusted Life Year gained. Pregnant smokers attending their first maternity booking appointment will be invited to participate. All participants will be offered usual smoking cessation services. In addition, the intervention group will be offered up to £400 of shopping vouchers, £50 if they attend counselling and set a quit date, £50 if proven quit 4-weeks later, £100 if quit after 12 weeks, and £200 if quit near the end of pregnancy. Self-report in late pregnancy and up to 6 months afterbirth will be verified by saliva cotinine, a nicotine metabolite.

HOW RESULTS OF RESEARCH WILL BE USED: Trial results will provide sufficient evidence for NICE (PH26) Smoking: stopping in pregnancy and after childbirth – to decide if financial voucher incentives should be recommended ornot.

3.0 INTRODUCTION

3.1 Background

Tobacco smoking is the leading preventable cause of cancer, accounting for more than 64,000 cases in the UK each year (Parkin, 2011). Lifelong smokers lose 10 years of life. Individuals who give up by age 40 avoid much of the morbidity (e.g. lung cancer risk is reduced to two times that of never smokers compared with 16 times for lifelong smokers) and early mortality of continued smoking (Doll, 2004). Smoking cessation by age 40 leads to a near normal lifespan. Latercessation only returns half the 'lost' years (Doll, 2004). Most pregnant women are younger than 40, so permanent cessation is very effective.

3.2 Rationale and justification for study

Around eighty percent of UK women have a baby (Portanti, 2009), making pregnancy an opportunity to help most women to quit before their health is irreversibly compromised. Stopping smoking during pregnancy also reduces the likelihood of offspring becoming smokers (Leonardi-Bee, 2011), reducing the future risk of cancer and heart disease. Few of the UK's current 130,000 pregnant smokers quit. NHS smoking cessation services offer counselling plus free Nicotine Replacement Therapy (NRT), however only 10% of pregnant smokers use these services and as few as 3% stop (Tappin, 2010).

Offering financial incentives to help pregnant women stop smoking has worked in local single site trials including the pilot study for this trial, CPIT II, in Glasgow (Tappin, 2015). The pilot study suggested a cost per Quality Adjusted Life Year (QALY) for financial incentives to support pregnant women to stop smoking of less than£500 per QALY (Boyd, 2016), well below the ceiling of £20,000 - £30,000 per QALY set by NICE for recommending the introduction of a new intervention strategy in the UK.

This study is a definitive trial of financial incentives in the UK as recommended by the National Institute for Health and Care Excellence (NICE, 2010). The level of incentive at £400 (about £60 per month from maternity booking to the end of pregnancy) is between the lowest level suggested by the general public that would be effective (£20 per month) and the highest acceptable level (£80 per month) (Morgan, 2015).

4.0 HYPOTHESIS AND OBJECTIVES

4.1 Hypothesis

Will the offer of financial voucher incentives in a simple transferrable format to pregnant smokers engage them in smoking cessation services and/or incentivise them to quit during and stay quit after pregnancy.

4.2 Primary objective

To assess whether a clinically and statistically significant doubling of smoking cessation rate by the end of pregnancy will be achieved by adding the offer of financial voucher incentives, for engaging with smoking cessation services and stopping smoking, to routine smoking cessation services in pregnancy.

4.3 Secondary objectives

- To compare quit rates at four weeks post quit date between women offered incentives and those receiving usual SSS care only
- To assess if women remain quit (cotinine or anabasine validated self-report) up to six months afterbirth (continuous abstinence)
- To assess if women have quit (cotinine or anabasine validated self-report) at up to six months afterbirth (point abstinence)
- To assess whether financialincentives are cost effective in terms of incrementalcost per quitter (at birth and up to six months postpartum) and perquality adjusted life yeargained
- To identify the effects of differences in smoking cessation services and demographic diversity of pregnant smokers on the effectiveness, cost effectiveness and transferability of this intervention
- To determine, if effective and cost-effective, how the intervention can be rolled out across the UK

5.0 STUDY DESIGN

A phase III pragmatic multi-centre individually randomised controlled, parallel group, superiority trial with an embedded economic evaluation and an embedded process evaluation – a mixed methods study. This study will be performed according to the Research Governance Framework for Health and Community Care (Second edition, 2006).

6.0 STUDY POPULATION

6.1 Setting and locations

The target population is pregnant smokers routinely identified at maternity booking. Participants will be recruited from three of the four UK nations – Scotland, England and Northern Ireland. The first centres to open will be Site D and Site C and, later, centres in the Wessex region.

6.2 Number of participants to be enrolled

We aim to recruit 940 participants. This will give 90% power and 5% significance to show a doubling of quit rate from 7% control to 14% incentives, allowing 15% loss to follow-up.

6.3 Eligibility criteria

631 1 Inclusion criteria

Womenwill be eligible to join the study if they meet all of the inclusion criteria:

- Self-reportedsmokers
- >= 16 years
- Pregnant < 24 weeks
- Live in the catchment areaof the participating NHS site
- English speaking forverbaltelephone consent

632 2 Exclusion criteria

Women will not be eligible for the study of they meet one of the exclusion criteria:

- Non-smoker
- <16 years
- Pregnant > = 24 weeks
- Non-English speaking

6.4 Withdrawal criteria

Participants may ask to withdraw from the study at any time without influencing their usual care or treatment. Participants who withdraw will not receive further follow-up and will not receive future vouchers. Data already supplied will be retained and used in the analysis. Participants may also withdraw from the intervention but remain in the trial for follow-up data.

6.5 Participant replacement

Participants who withdraw from the study will not be replaced.

7.0 INTERVENTION

The intervention group and control group will receive the offer of smoking cessation services support which includes a face to face counselling appointment (in some areas within individual trial sites, the face to face contact may be within pharmacy services as in Site D where a private room is used as the place for face to face support). Participants in both groups will receive a £50 shopping voucher if they provide data at the primary outcome time point (late pregnancy) and a £25 shopping voucher if they provide data at the secondary outcome time point (Up to 6 months post- partum). (If a participant does not provide primary outcome data, they are still able to receive a £25 shopping voucher if they provide secondary outcome data).

7.1 Intervention group

In addition, the intervention group will receive the offer of up to £400 of shopping vouchers: £50 for attending the first routine face-to-face counselling appointment at the smoking services support meeting and setting a quit smoking date, £50 if quit 4 weeks later verified by exhaled carbon monoxide (CO), £100 if CO verified quit 12 weeks post quit date, £200 if CO verified quit at 34-38 weeks gestation. (This means that intervention participants who do not use NHS Smoking Cessation Services can still receive the final £200 payment if they are verified quit at the primary outcome point in late pregnancy).

Adherence to the intervention will be assessed by considering distribution and receipt of shopping vouchers, which will be confirmed by Royal mail signature.

7.2 Control group

The control group will receive the offer of smoking cessation services support which includes a face to face counselling appointment. They will also receive a £50 shopping voucher if they provide data at the primary outcome time point (late pregnancy) and a £25 shopping voucher if they provide data at the secondary outcome time point (up to 6 months post-partum).

8.0 OUTCOME MEASURES

8.1 Primary outcome measure

Smoking in late pregnancy at 34-38 weeks gestation corroborated by urinary or salivary cotinine (or anabasine if taking NRT) for women who self-report as quit when contacted by the Trial Team.

8.2 Secondary outcome measures

Set a quit date with smoking cessation services before 26 weeks gestation

Biochemically validated (CO) self-reported abstinence from smoking at four weeks after quit date

Cotinine validated (or anabasine validated) smoking cessation up to six months after birth (point abstinence)

Cotinine validated (or anabasine validated) smoking cessation from late pregnancy up to six months after birth (continuous abstinence)

Birth weight

Cost-effectiveness

Process evaluation

Data for the primary outcome and for three secondary outcomes (setting quit date, four week quit status and birth weight) will be combined with data from the feasibility trial in a meta-analysis (see Section 15),

9.0 STUDY TIMELINE

See Appendix 1

10.0 METHODS AND ASSESSMENTS

10.1 Recruitment

10.1.1 1 Initial screening and permission to pass contact details to the trial team

Women identified as self-reported smokers at maternity booking appointments in each centre are routinely referred to NHS SSS. A combination of referral pathways to SSS are in operation. Some centres operate an 'opt-out' referral policy i.e. referral occurs automatically (as recommended in NICE guideline PH26 Smoking: Stopping in pregnancy). Others operate an 'opt-in' policy i.e. women agree to referral when asked by their maternity booking midwife. Information passed from NHS maternity services to NHS SSS includes data (i.e. date of birth, expected date of delivery) to allow smokingcessation advisors to determine if women are eligible to join the study.

In order to optimise recruitment we will raise awareness of the trial in this patient group by placing posters and flyers/shortened version of approved PIS in local antenatal clinic waiting rooms and in antenatalinformation packs.

Initial routine contact with women by smoking cessation advisors is usually by telephone, but may on occasion, be face to face. If no contact is made, a letter may be sent to pregnant ladies who smoke (as happens in Site D) informing them that a service is available to help them stop. In this letter will be a paragraph introducing the trial. For Site D this paragraph will be: 'You may also be suitable to take part in a study that we are currently running. The study wants to find out if giving pregnant women an incentive will help them to stop smoking. You could potentially obtain up to £400 in high street shopping vouchers if you stop smoking with our service.' During this routine telephone contact to engage women with SSS, smoking cessation advisors will verify women's eligibility to participate in the study using an electronic screening log (reasons for ineligibility will be recorded and retained without direct patient identifiers), provide study information using a pre - determined script, and where appropriate, request verbal permission to pass contact details and CO results taken as part of their routine care to the study team (including the Call Centre). The advisor will then continue their 'usual' smoking cessation support which includes the offer of a face to face appointment to discuss smoking with the aim of setting a quit date.

Women who give permission for their contact details to be transferred will be informed that they will receive a) a study information sheet in the post, b) a SMS from the Trial Contact Centre (TCC) informing them that they will be telephoned in a few days, and c) a telephone call from the TCC to enrol them in the study. They will be given the phone number of the TCC so they feel safe to answer a call from that number. Contact details of eligible women who agree to study contact will be transferred to the Study Team using an agreed secure File Transfer Protocol (sFTP). This process was used in the feasibility study (Tappin 2015) following agreement by both the NHS GG&C Caldicott Guardian and security advisers. On receipt of personal details at the TCC a letter and a Participant Information Sheet (PIS) willbe automatically sent to women by post.

In addition, to improve the TCC's rate of contact with the participant, following the patient's permission to be contacted to enrol in the trial, researchers at the site may phone patients from a local telephone number to answer any questions that they may have and highlight that to enrol in the study they will need to answer the telephone call from the 0800 number stated in the PIS. Womenwho do not give permission for their contact details to be passed to the study team will be asked (where agreed at local site) if they are willing to be contacted by a researcher interested in

speaking with them about their decision not to find out more or participate in the trial. If interested, they will be asked if their contact details can be passed to the researcher who will send them a separate information sheet (Discussion Information Sheet) by post and follow this up with a phone call within seven days. The contact details of women consenting to the interviews will be passed to the process evaluation team researcher and not the main study team. A member of the process evaluation team will then contact the women within seven days by phone to see if they would be willing to take part in an interview lasting approximately 5 to 10 minutes. Women that take part in an interview will receive £25 in Love2Shop vouchers.

All women will receive the usual SSS that is available in each centre regardless of whether or not they have given permission for further study contact. This will be emphasised to women during the initial routine contact by smoking cessation advisors.

10.1.2 2 Informed consent

On receipt of the contact details for women who gave permission for these data to be passed, the TCC will send a SMS to inform women to expect a phone call in the next few days from the TCC number to enrol them in the study. A few days later the TCC will then telephone women to conduct study enrolment. At the beginning of the call, women will be informed that the call will be audio-recorded and kept as proof of consent (Tappin, 2015), and that it may be used for analysis purposes as part of the process evaluation. The TCC advisors will:

a) verify that women have received the study information sheet and have had time to read it. Where this is not the case, women will be given the option to request that another information sheet is sent to them by post and the enrolment telephone call rescheduled;
b) inform women about the study in detail using an agreed script (Tappin, 2015) similar to the content of the PIS. This will include the following:
   - what would be involved if they decide to take part i.e. the number of contacts/follow-ups that will be made by the team
   - how allocation to one of the two study groups will be made at random
   - the different voucher values in the two groups of the study
   - if they join the study and the TCC are unable to contact them at the end of the study to obtain their smoking status a Research Nurse will seek confirmation of their address and phone number from hospital records, and if required from their GP
   - that pregnancy data collected routinely will be made available to the Trial Team e.g. gestation at birth and birth weight, maternal height/weight
c) answer any questions they may have, and
d) obtain verbalconsent for taking part in the study (Tappin, 2015)
   Consent will also be asked:
   - for a member of the process evaluation team to contact them with a view to sharing their study experiencewith a researcher
   - to re-contact them in the future (i.e. after the end of the study) to assess long term effects on mother and child if incentives prove to be effective
   - to use residual routine blood samples taken from them in pregnancy for research purposes. They will also be informed of who to contact if they later decide that their samples should not be retained

- After explanation about frequent changes to mobile phones and sometimes addresses, permission will be sought to make contact via telephone, text, letter or contact using a private message via Facebook and email to help achieve complete outcome assessment

Refusalof consent for any/all of these additional processes will not preclude women from taking part in the main trial.

Confirmation or otherwise of consent will be recorded directly into the trial database during the telephone consent call. This will trigger the dispatch of a hard copy of the consent form to the participant with the TCC advisor's signature together with a further copy of the PIS.

Baseline data will be collected at this point. This includes: household income, area based measures of socio-economic status, age, previous pregnancies, weight, height, expected delivery date, EQ-5D, Fagerstrom score for Nicotine Dependence and already using NRT and/or e-cigs.

Participants' General Practitioners (GPs) willbe informed that they are taking part in the study.

Potentialparticipants who cannot be contacted by the TCC (ECHO Managed Services Limited) despite passing over their contact details, will be sent a short SMS by the process evaluation team and asked to text YES back to indicate that they are interested in taking part in an interview and agreement for a researcher to call them to arrange a date/time. The text will aim to establish contact and enable arrangement of an interview only. Interviews with this group of women will be 5 to 10 minutes. This group of women do not require a separate information sheet as they will have received the main trial PIS after passing over their details. Women that take part in an interview will receive £25 in Love2Shop vouchers.

10.1.3 3 Randomisation

After collecting all the required information the TCC advisor will use the trial database to randomly allocate the trial participant to either intervention or control. The participant will be informed of their random allocation (including details of the values of the vouchers offered and time points at which they will be offered) over the phone. Some participants may be disappointed not to be allocated to the intervention group and the TCC advisors will be trained to respond appropriately to this.

Participants will be allocated 1:1 ratio to either intervention or control using random permuted blocks with randomly varying block sizes. In addition, a random date between 34 and 38 weeks gestation for each pregnancy will be generated as the date for primary outcome data collection. The randomisation schedule will be generated by York Trials Unit and integrated into the online trial database hosted by ECHO Managed Services Limited.

10.1.4 4 Blinding

It will not be possible to blind participants to the intervention. The Trial Team (Contact Centre) collecting primary outcome self-report of smoking cessation will be blind to allocation. Women will be asked not to disclose group status during the follow-up telephone call with the TCC. The statistician conducting analyses will have no contact with women but will not be blind to treatment allocation.

10.2 Study visits and procedures

ECHO Managed Services Limited will be responsible for sending financial incentives to participants. Registered post allows tracking and also view of 'signature for receipt' by the trial team. Vouchers are redeemable in many outlets. The voucher company provide a breakdown of where the vouchers have been redeemed e.g. Mothercare, Argos etc. Below details the study visits and data collection procedures of the trial up until the COVID-19 pandemic in March 2020. Within Appendix 2, Table 1 'proposed alternative protocol B' provides details of changes to these procedures in light of the pandemic which are intended to remain in place until the end of the trial, as agreed by the study sponsor GG&C.

10.2.1 1 Initial face to face appointment with smoking cessation advisor (Intervention and control groups)

Intervention participants who arrive for a face-to-face appointment or engage with services via telephone and set a quit date will be sent a financial incentives voucher of £50 via registered post. The research nurses or administrators will contact the SSS to assess participants' engagement with standard SSS including if they have set a quit date and if they are using e-cigs or NRT. Quit date is often set at the first face-to-face appointment or telephone contact with the SSS. If this does not take place, smoking cessation advisors will be able to advise the Trial Research Team, by text or phone, if a quit date is set later. However, the set quit date should be before 26 weeks gestation.

10.2.2 2 At 4 weeks post-quit date (intervention and control groups)

Data provided by Stop Smoking Services:

CO data routinely collected: If a participant (in the intervention or the control qroup) has quit smoking and a CO reading has been taken as part of routine care, then the CO result will be recorded on the trial database. CO results for the control group will only be recorded where this information is available as part of routine care; attempts will not be made to collect this information from participants in the controlgroup for trial purposes.

Contact by smoking cessation advisor: The smoking cessation advisor will meet or phone pregnant smokers who have set a quit date including trial participants and contact the Trial Team to advise how the quit attempt is progressing. If the smoking cessation advisor does not make contact with the Trial Team, the Trial Team will contact the advisor (for intervention participants only) to verify smoking cessation quit attempt progress.

Contact by a research nurse (intervention participants only): If participants self-report as quit and are in the intervention group, a research nurse will contact the participant and make an arrangement for face-to-face contact to biochemically verify smoking cessation using a CO breath test monitor. If the CO level is at the accepted level for a non-smoker at the site (site specific CO cut-off levels agreed with Sponsor) the participant will be sent a second incentives voucher for £50 through the post (automatically sent by ECHO Managed Services Limited Fulfilment House when database item is completed). The research nurse will record CO reading on the trial database. The CO reading is purely to decide if the incentive payment should be dispatched. If the service has taken a CO reading at the time of self-report at 4 weeks and the reading is available then another CO will not be taken by the research nurse.

10.2.3 3 At 12 weeks (3 months) post-quit date (only intervention participants who self- report quit at 4 weeks)

<u>Data provided by Stop Smoking Services:</u>

CO data routinely collected: If a participant in the intervention qroup has not quit smoking and a CO reading has been taken as part of routine care, then the CO result will be recorded on the trial database. CO results for the control group will not be recorded in the trial database where this information is available as part of routine care; attempts will not be made to collect this information from participants in the control group for trial purposes.

Contact by smoking cessation advisor: All clients who self-report quit at 4 weeks are routinely contacted by their smoking cessation advisor about 12 weeks after theirquit date to ascertain how the quit attempt is progressing. This information will be passed to the Trial Team.

Contact by trial team (intervention participants only): If no contact is made by the smoking cessation advisor, self-report of smoking by text message or telephone contact will be sought by the Trial Team from intervention participants who had quit at 4 weeks.

Contact by a research nurse (intervention participants only): If participants self-report as quit a research nurse will contact the intervention participants and make an arrangement for face -to-face contact to biochemically verify smoking cessation using a CO breath test monitor. If the CO level is at the accepted level for a non-smoker at the site the participant will be sent a third vouchers incentive for £100 via registered post. The research nurse will record the CO reading on the trial database. The CO reading is purely to decide if the incentive payment should be dispatched.

10.2.4 4 34-38 weeks gestation (all participants)

<u>Primary outcome data collected by the Trial Contact Centre:</u>

Contact by TCC: All participants will be contacted by telephone by the TCC at a random time (built in to the trial database between 34-38 weeks gestation) near the end of pregnancy to ascertain current smoking status. Trial research nurses will review womens' notes one week prior to the telephone contact to check health status of mother and baby and alert TCC staff to any adverse events. Participants will be asked the following questions:

1. Have you smoked at all in the last 8 weeks (Yes/No)

2. If Yes, have you smoked more than five cigarettes in total in the last 8 weeks (Yes/No)

If the answer is 'No' to question 1 <u>or</u> 'No' to question 2, the participant will have self-reported as a 'non-smoker' forthe primary outcome of the trial.

3. A further question for self-report quitters about continued use of nicotine replacement therapy and electroniccigarettes will also be asked (Tappin, 2015).

4. All will be asked for EQ-5D data forhealth economicanalysis.

Questions will also be asked about level of use throughout pregnancy of e-cigs and NRT including whetherthese were prescribed orpurchased.

If the TCC is unable to make contact with the participant, the local Research Administrator or Research Nurse will make further enquiries for contact e.g. contacting GP surgery to ask about new

address or telephone numbers. Three further attempts at telephone contact will be made followed by three text messages (Tappin, 2015), a personal Facebook enquiry (individuals tend to keep their Facebook page but mobile phone/text and address can change frequently) and finally a paid return letteras in the feasibility trial (Tappin, 2015).

All those who self-report as quit will have a face-to-face meeting arranged with a Research Nurse (either in a clinic or home setting as suits the participant) to biochemically verify smoking cessation with a CO breath test to decide if the incentive payment should be dispatched and to collect a saliva (or urine) sample for cotinine estimation (Tappin, 2015) (or, for participants taking NRT, anabasine estimation) as the primary outcome.

To minimise loss to follow-up all participants who self-report as still smoking in late pregnancy, or who report as quit and provide the biochemical samples will receive £50 (automatically sent by registered post by ECHO Managed Services Fulfilment House when information is keyed into the trial database by trial team staff) as a 'thank you' for providing this important information. Those in the intervention group who have a CO breath test levelat the accepted levelfor a non-smoker at the site will also receive a finalvoucher incentives payment of £200.

If a participant is lost to follow-up and, has given permission to use their residual routinely collected blood sample, the Trial Team will contact the local laboratory services to obtain any available residual blood samples from late pregnancy to be sent for cotinine testing. This was undertaken in the feasibility trial (Tappin 2015) and allowed us to demonstrate that those lost to follow-up (where samples were tested) in both the intervention and controlgroups had all relapsed to smoking.

Incentive payments at 4 and 12 weeks post-quit date (see 10.2.2 and 10.2.3) and 34-38 weeks gestation are based on the SSS accepted CO levels for a non-smoker at each site. In routine practice an element of discretion is exercised around these CO levels to allow for passive smoking. The trial will thus adhere to the same principles and consideration will be given to individual cases (agreed with sponsor).

1025 5 Up to 6 months post-partum (calculated from the expected date of delivery) (all participants): cotinine validated quit

Participants enrolled in the trial prior to 31/12/2019 will be followed-up at 6 months postpartum, this will be approximately 65% of the participating sample. Women recruited later will be followed up at the latest point postpartum until 31/10/2020 where data collection ceases. Thus, some trial participants will be followed up between 0 and 6 months postpartum. Adapting a variable follow-up model for participants recruited later in the trial will allow some postpartum data capture from all participants.

Secondary outcome data collected by the TCC:

Contact by Trial Team: The TCC will telephone all trial participants to ascertain smoking status. EQ-5D data will also be collected at face-to-face meeting or over the phone. All those who self-report as quit at post-partum follow-up will have a face-to-face meeting with a research nurse to biochemically verify smoking cessation with a CO breath test and to collect saliva (or urine) for cotinine estimation. During collection of samples, the participants will be asked if they are using NRT or e-cigarettes. Forparticipants, who answer 'yes' urine samples will be tested foranabasine which

comes from tobacco and not NRT/e-cigarettes. All participants who self-report as smoking at postpartum follow-up , or who report as quit and provide the biochemical samples will receive £25 as thanks for providing this important information. In the event a participant delivers her baby beyond the data collection phase (31/10/2020), they will automatically receive the £25 postpartum follow-up incentive. Biological samples of saliva and urine will not be available for use by other researchers.

1026 6 Discontinuation visit and procedures

If a woman miscarries during pregnancy, the research nurses will highlight this where known (e.g. by having access to maternity records) prior to the late pregnancy telephone contact. This was achieved by close links with the SSS and midwifery staff via patient notes in the feasibility study (Tappin 2015). The TCC staff will be sensitive to this. A judgement willbe made by the trial and local managers about further contact. If the participant is contacted and wishes to continue in the trial, they will be encouraged to do so. Based on the pilot study of 612 pregnant smokers at maternity booking, the numberof miscarriages was small (less than 10).

11.0 ECONOMICEVALUATION

11.1 Process and Analysis

The economic evaluation will utilise resource and cost data – financial incentives, SSS delivery and NRT – as well as trial outcome data – quit rates and birth weight. We will use the EQ-5D as the measure of utility, which will be collected at baseline (consent call) and at follow-up (late pregnancy primary outcome and up to and including six months postpartum secondary outcome). The literature will provide long term health outcomes formother and baby.

The economic analysis will assess whether offering financial incentives in addition to routine SSS is cost-effective, using an NHS perspective for cost year 2020. Best practice guidelines and the NICE reference case will be adhered to (NICE 2013, Husereau, 2013).

Within trial analysis: Resource use data from the trial (contacts with cessation advisors, NRT and cessation aids, financial incentives) will be collected along with primary outcome data (cotinine validated self-reported quits at 34-38 weeks gestation and up to six months post-partum reportedas incremental cost per quitter). Multiple imputation will account for missing data assuming data are missing at random, so that missingness can be predicted by othercomplete cases.

EQ-5D will be collected at the baseline consent call after consent for the trial has been obtained, again at the primary outcome call in late pregnancy and finally at the secondary outcome call up to six months afterbirth.

Lifetime analysis: A longer term analysis will incorporate additional costs for low birth weight and premature births, and account for smoking relapse post-partum (using the postpartum trial data). Lifetime analysis will adapt a published probabilistic decision analytic model to examine incremental cost per QALY gained, presenting confidence limits around cost and QALY outcomes. Extensive sensitivity analysis will include (i) re-analysis to account for gaming identified, (ii) self-reported outcomes, and (iii) additional cost for smoking related disease.

11.2 Gaming

Audit of cotinine levels in leftover blood from routinely collected blood samples taken in late pregnancy will detect ‘gaming’ on an individual level to appear as a quitter in late pregnancy (i.e. stopping smoking a few days before sample collection). Gaming identified will only be used as part of sensitivity analysis to assess the robustness of the trial results. Individuals will not be ‘accused’ of gaming and will receive incentives if their CO levels are at the accepted level for a non-smoker at the site at the incentive timepoints regardless of any cotinine values measured. (In reality cotinine measurement is undertaken on a batch basis usually weeks after the sample has been taken (Tappin 2015) and the two issues are separate for the researchers and only brought together at the time of analysis).

Late pregnancy residual routine sample collection allowed us to assess the proportion of women who had quit smoking judged by saliva cotinine who had in fact relapsed at some point within the eight weeks prior to the primary outcome collection. Again this system (Tappin 2015) allowed us to

check and show that 80% of both the intervention **and** the control group who were saliva cotinine quit in late pregnancy were also cotinine negative on a blood sample taken for a different reason in late pregnancy (i.e. that gaming of the primary outcome to show abstinence was at an acceptable level).

12.0 PROCESS EVALUATIONRESEARCH

12.1 Study Design

The aims of the process evaluation are:
1. To reduce the risk of the trial failing due to poor enrolment, by understanding the mechanismsof identification and referralof pregnant women to smoking cessation services in each area, and
2. To understand the varied contexts in which smoking cessation services operate so that facilitators and barriers to implementation in a range of contexts, including diverse populations can be identified.

This is a mixed methods process evaluation, following a case study design conducted in two stages. Stage 1 (set-up) will consist of field notes from structured observations, unstructured interviews with key staff and routine data collection in order to understand the fit between CPIT, usual care services and local context. Stage 2 (trial) will consist of semi-structured audio-recorded interviews and recordings of monthly meetings held at each trial site.

Stage 1: During trial set-up
Structured observations will be conducted to record trial recruitment and processes discussed between site staff and researchers in the pre-trial training workshops. A range of possible strategies (e.g. text message, phone, post) for referral of women to SSS and recruitment are proposed to ensure optimal fit between the trial and usual services. These observations will be recorded via observation templates and unstructured interviews will be recorded in field notes. Baseline routine quantitative data (most recent 12 months) on SSS referral, engagement and attrition for each site will be accessed for each of the case studies. The service assessment will also establish the characteristics of each cessation service and usual care for pregnant smokers. This will be supplemented by unstructured interviews recorded in field notes with key staff that can provide any further details required in order to fully establish the context for conducting the trial. During this 'service assessment' phase, trialparticipants will not be directly involved.

Stage 2: Sampling and recruitment during internalpilot
Informed by data collection from stage 1, we will recruit professionals involved in trial implementation and service delivery in each of the sites to take part in semi-structured interviews. The total case study sample size and sampling strategy will depend on data saturation for the research question: how can we optimise recruitment and retention in the trial for each service. However, based on previous experience, this will be a purposive sample targeting the following sampling characteristics: high and low recruiters; 2-4 Smoking Cessation staff; 1-2 service managers; and 3-4 midwives. Contact centre staff (1-2) will also be sampled to take part in an interview with regard their perspective on recruitment. Recruitment will be facilitated by site leads and contacts made in stage 1. Information sheets and consent forms will be sent to all participants in advance of interview. Potential participants will be given an opportunity to ask questions about taking part and will be given assurances that their decision whether or not to take part in interviews will not affect their involvement in either the trial or provision of SSS support. They will be informed that they are free to withdraw from the study at any time without giving a reason.

Women eligible to take part in the trial (e.g. those who take part and those who don't) will be identified by the NHS SSS. The first contact from the SSS is usually by telephone to introduce the

service and to ask pregnant smokers if they would like help to change their smoking habit. Many services to the public, for instance banking services, audio-record contacts with clients after informing clients that this will happen for training purposes to improve the service. We propose that the SSS for pregnant women in the trial sites adopt this approach for the period of the trial, and if they find it useful as a training tool, to continue this practice thereafter. This will be put forward as a service development to be made by the SSS in each area. The contact centre is already recording the consent call made by the trial team to document verbal consent procedures have taken place and the participant has consented to take part in the trial.

At the points of discussion of the trial with smoking cessation advisors during the first SSS telephone contact' and during 'consent for enrolment in the trial by the TCC permission will be requested to pass contact details to a process evaluation researcher for potential participation in an interview. In addition consent will be requested for qualitative researchers to listen to and transcribe anonymised recordings at both timepoints. Using direct review of anonymised 'consent' events has been shown to improve the efficiency and of trial recruitment (Donovan 2016). Women who decide not have their contact details passed to the trial team will be asked if they can be contacted by a researcher to talk about their decision not to become involved further and given a discussion information sheet in relation to that contact.

By including permission to be contacted by a researcher at this point and obtaining verbal consent prior to interview (see below), the process evaluation team hope to reduce the information burden on women, some of whom may be harder to engage than others. All potential participants will be entered into a sampling frame and will be contacted if they meet ongoing sampling criteria. In order to complement the CPIT pilot sample/results (deprived, Caucasian women) this sample will purposively select more affluent and 'Black or Minority Ethnic' women using an iterative process informed by concurrent analysis whichwill include live trial eligibility, recruitment and attrition data.

Prior to the interview taking place, the interviewer will confirm consent to take part by obtaining verbal consent. The researcher will emphasise that taking part in an interview is voluntary, will not have any bearing on their status as trial participants, other medical treatment or smoking cessation support and that they are free to withdraw from the study at any time without giving a reason. Participants will be given the opportunity to ask questions about the study prior to providing verbal consent. Prior to the formal (usually) telephone consent process, the qualitative researcher will discuss audio-recording of interviews and the consent process which will be kept as a record of the consent process.

Additionally, to enhance learning from qualitative data and to compensate for gaps in data collection due to the COVID-19 pandemic, a brief, online, anonymous survey will be distributed to maternity and smoking cessation service professionals in each of the seven trial sites towards the end of the trial. Professionals will be asked to provide their views and feedback on the operation of CPIT III in their local area. This survey sub-study will be led by the process evaluation team at the University of Stirling with assistance from trial researchers at Queen's University, Site C to analyse survey responses.

12.1.1 Data Collection

Semi-structured qualitative interviews with women who consent to take part in the trial will take between 30-45minutes depending on what interviewees have to say and time they are available.

Qualitative interviews withwomen who are eligible but decline to have contact details passed to the contact centre and with women who do give permission but subsequently cannot be contacted by the contact centre will take 5 to 10 minutes.

The interviews will be semi-structured, following an iterative approach informed by on-going analysis, to explore some areas in more depth or follow potential new lines of inquiry. This will be supported by input from FH (qualitative expertise) and PH (Clinical and qualitative expertise) drawing on further expertise from the wider team as required. All interviews will be conducted by telephone by an experienced qualitative researcher. Telephone interviews are particularly preferred with regard to speaking with pregnant women, in order to avoid any stress or inconvenience that may be caused by a researcher visiting their home. Participants will be assured that any quotes used in reports or publications will remain anonymous. Transcripts will be anonymised and will only be shared with members of the research team involved in the process evaluation analysis. This will exclude site leads/local PI's in order to ensure that professional views and those of trial participants remain confidential. It is acknowledged that clinical staff in particular may well be identifiable therefore care will be taken with reporting to ensure that all quotes used are not attributable to individuals. Further information related to the two categories of interviewees are addressed separately below.

1. Semi-structured interviews with professional staff in the four sites. These interviews, which will be face to face as well as over the phone, will explore: barriers, facilitators and contexts of conducting the trial (local capacity, organisational structures and any changes to these); fidelity to trial processes (recruitment to the trial, information given to patients, training issues) (see topicguide).

2. Interviews with women participating in the trial will primarily explore attitudes to taking part in the trial including barriers and facilitators. Some attention will also be paid to previous quit attempts in order to contextualise the current experience. Further data will be collected regarding attitudes to smokingwithin their particular community/demographic (see topicguide).

Interview and observational data will be supplemented by field notes of regular meetings between the implementation teams (e.g. trial managers, smoking cessation staff and midwives) in order to reflect on recruitment data, and barriers and facilitators to trial implementation. Lessons learned regarding successful referral and recruitment strategies will be shared across sites in order to facilitate best practice in trial recruitment. Any changes or improvements made to these processes will be included in data analysis. In addition, a random sample of anonymised audio-recordings of the 'discussion of the trial with smoking cessation advisor at first smoking cessation service telephone contact' and 'consent to trial' processes from participants at each of the sites will be analysed in order to understand any variation in recruitment rates between sites.

The brief, online survey for professionals working in maternity or smoking cessation services within CPIT III trial sites will be constructed using the online survey tool, Online Surveys (https://www.onlinesurveys.ac.uk/), which will produce an internet hyperlink to the survey. The aim of the survey is to provide an opportunity forrelevant staff involved with maternity care and smoking cessation services to contribute their perspectives on the trial, relevant to future implementation and research. Trial sites will be allocated a unique identifier and this will be added to the survey to provide anonymity forworkplace. This will result in the production of seven versions

of the same survey for sending to corresponding trial sites. A key to the unique identifiers will be stored securely at University of Stirling and only known to the process evaluation team. Key contacts located at trial sites will be asked to arrange for the distribution of a standardised email containing summary information about the trial and the survey alongside the survey hyperlink. A mass email will then be sent to individuals via established staff email lists. The email will include a deadline for completing the survey of within 4 weeks, with a reminder email sent after 2 weeks and another sent after 3 weeks (a few day before the deadline), reminding recipients of a last chance to contribute.

The survey will take up to 15 minutes to complete and begin with some questions about the participant, including their service area and level of responsibility but will avoid the collection of personal data including name, contact details and job title. Questions will then be focused on the trial and use a variety of formats for providing answers, including open-ended text boxes, Likert scales and 'yes/no/don't know' tick boxes. Submitted survey responses will be stored in a database supported by, and accessible via, Online Surveys. Once the survey deadline has passed, responses will be copied by researchers to a secure, password protected university computer to facilitate analysis.

12.1.2 Data analysis

The framework approach will guide the analysis of qualitative data: identifying themes, coding, summarising, constructing matrices to compare and contrast themes and concepts across SSS delivering the trial and seeking patterns, explanations and potential solutions for the observed recruitment outcomes. Transcribed interviews, observations and quantitative data for actual and expected recruitment targets will be entered into QSR NVivo (v12) software to facilitate the organisation of data. Analysis will describe baseline patterns of service provision and changes that take place during the trial and will assist in understanding any observed differences in reach or outcomes between SSS. A description of the consequences of introducing financial incentives will be made, which will be of value for future implementation and service provision.

Responses to the online survey distributed amongst professionals will be analysed and reported descriptively by anonymised site, using quantitative data summaries where appropriate. Free text comments will be included in the analysis of the qualitative data. The findings will serve to triangulate the final process evaluation analysis.

12.2 Data Handling

The process evaluation will fully comply with the terms of the Data Protection Act 1998. When participants have been recruited into the study and given informed consent they will be assigned a non-identifiable code and all data (paper and electronic) will use this code. Identifiable data (e.g. contact details) will be held on a separate database (i.e. will not be linked to any data) and will only be used to contact the participant about the study. Interviews will be digitally recorded and transcribed verbatim in order to ensure fidelity to the views of interview participants is retained in the analysis.

All data will be held on a secure, password protected University of Stirling servers. The analysis will be undertaken by the process evaluation researchers and they will be the only members of the team

who will have access to field notes, audio-recordings and anonymised interview transcripts. The analysis will take place on University computers at the University of Stirling.

Data collected via the online, anonymous survey of professionals will be moved from the survey tool, Online Surveys, used to facilitate the survey, to the University of Stirling's computer network, only accessible via password protected computers. It will then be encrypted and securely transferred via OneDrive cloud data storage (located in data centres based in the UK) to CPIT 3 researchers based at Queen's University, Site C and stored within the University's computer network there, again only accessible via password protected computers. Analysis of survey responses will be undertaken by the trial researchers in Site C but they will be blinded to site information with only the process evaluation team at Stirling having access to the trial site code. The Site C trial researchers will share the anonymised findings with the University of Stirling Process Evaluation team to enable the findings to be incorporated in the final process evaluation.

Digital voice recordings will be destroyed at the end of the study. All other recordswill be retained in a secure archive setting for 10 years to facilitate future analysis and publication of the study material.

12.3 Ethical issues related to the process evaluation

Informed consent procedures will ensure that participants understand that participation is entirely voluntary and that they can withdraw from the study/interviews at any time without this affecting their trial participation or other medical treatment. Participants can participate in the trial without participating in the process evaluation. While every precaution will be taken to preserve patient anonymity and confidentiality there will be limits to this. In the event that the researcher has concerns for the well-being of a participant or others, action would be taken to disclose concerns to a named clinical contact within the relevant intervention site though the researcher would speak to the participant about this first. If a participant (either relevant professional or pregnant woman) were to disclose anything indicating unsafe practices or misconduct, they would be urged to follow hospital complaints procedures.

To facilitate anonymity in the online survey of professionals, respondents will not be asked to provide identifiable data and therefore it will not be possible to link responses to individuals at a later point. 12.4 Payment fortaking part in interviews

As a thank you for taking part in an interview and to compensate for their time, pregnant women will be sent a £25 Love2Shop voucher.

13.0 SAFETY ASSESSMENT AND REPORTING

13.1 Definitions

Adverse Event (AE): Any untoward medical occurrence in a trial participant which does not necessarily have a causal relationship with the treatment.

Serious AE (SAE): Any adverse event that a) Results in death; (b) Is life threatening; (c) Requires hospitalisation or prolongation of existing hospitalisation; (d) Results in persistent or significant disability or incapacity; (e) Consists of a congenital anomaly or birth defect; or (f) Is otherwise considered medically significant by the investigator.

Related SAE: Any SAE defined as due to the administration of any research procedure. The relatedness of an event will be reviewed by a Principal Investigator and two members of the TMG.

Expected SAE: It is expected that there may be incidents of still birth, miscarriage, congenital anomaly or birth defect and hospitalisations. Such events, which are deemed unrelated to the study, will not be reported as AEs to the ethics committee but will be reported as outcomes.

Unexpected SAE: Any SAEdefined as a type of event not listed above as an expected occurrence.

13.2 Collecting, recording and reporting of adverse events

We will record all SAEs that are related to taking part in the study. We will report to the Research Ethics Committee only details of any SAEs that are related to taking part in the study and are unexpected. Non-SAEs will not be recorded or reported for this study unless they are related to being in the study or are related to the intervention.

Details of any SAEs reported to the Trial Managerwhich are related and unexpected, willbe recorded using a study adverse event form. The AE reporting period for this study begins as soon as the participant consents to be in the study and ends after their final data collection. The Trial Manager will inform the Principal Investigator who will decide if the event should be reported to the main REC as an SAE. Related and unexpected SAEs will be reported to the main REC within 15 days of the Principal Investigator becoming aware of the event. A copy of the SAE Form will be stored at the recruiting site in the CPIT Portal. The occurrence of adverse events during the trial will be monitored by the Trial Steering Committee (TSC). Due to the low risk nature of this trial, the TSC will take on the role of the Data Monitoring Committee (DMC). The TSC and sponsor will immediately see all SAEs thought to be treatment related and the TSC, and the TMG, will see SAEs not thought to be treatmentrelated at the next scheduled meeting.

14.0 DATA MONITORING AND QUALITY ASSURANCE

14.1 Audits and inspections

The study may be subject to inspection by any of the funding organisations. The study may also be subject to inspection and audit by NHS GG&C undertheir remit as sponsor.

14.2 Data quality assurance

NHS Greater Glasgow and Clyde is the lead sponsor for this study. The study will be fully compliant with the Research Governance Framework.

Data monitoring will be conducted at sites on an 'as-needed' basis and dependent on factors such as recruitment rate and raising of any concerns and by following the progress remotely to ensure integrity of randomisation and data collection. Audits of enrolment, participant retention and outcome assessment will be performed by a trial co-ordinator and/or monitor from York Trials Unit. Written reports will be produced for the TSC (TSC to take the role of the DMC) informing them of any corrective action that is required.

14.3 Data entry, data management and storage

Participants' contact details, baseline data and outcome data will be directly entered as it is collected by the Trial Team at the sites on the ECHO Managed Services Limited database. The TCC at ECHO Managed Services Ltd will also enter data as it is collected from participants. Minimal outcome data will be collected on hard copy CRFs.

Electronic copies of participant consent forms (completed by the TCC advisor at the time of the trial consent call) will be stored within ECHO Managed Services as will audio-recordings of the consent call.

Personal data such as patient contact information and outcome trial data will be stored securely on the online portal database (hosted by ECHO Managed Services) and accessed by the Trial Team and TCC. Personal information will be transferred between NHS maternity services, SSS and the Trial Team by a sFTP protocol as utilised in the feasibility trial and agreed by NHS security advisors in each centre.

When the trial information is required (e.g. for data quality checks, the TSC and at trial completion for final analysis) the data will be downloaded without direct identifiers by ECHO Managed Services and will be stored in NHS GG&C R&D department in a password protected archive and also at York Trials Unit, on their secure network servers. The trial information will include data on trial participants and the limited data on pregnant smokers who gave consent for information to be passed to the Trial Team but who declined to take part in the trial or who could not be contacted for consent to the trial. All data storage will adhere to Good Clinical Practice guidelines.

Laboratory results will be returned electronically from ABS laboratories to the trial team at NHS GG&C R&D department and entered onto the trial database via the secure portal system. These results will be stored for later data checks, and longer term storage adhering to Good Clinical Practice guidelines.

All data will be kept for 10 years in line with the NHS GG&C/University of Glasgow's Research Governance Framework Regulations for clinical research. This data will be stored confidentially on password protected servers maintained on the University of Glasgow network.

15.0 STATISTICAL CONSIDERATIONS

15.1 Determination of sample size

940 participants will give 90% power and 5% significance to show a doubling of quit rate from 7% control to 14% with incentives, allowing 15% loss to follow-up. The figure of 7% in the control group was derived from our recent pilot trial in Glasgow (8.6% abstinent, Tappin 2015) and in two other recent large trials of pregnancy cessation interventions, conducted in regions where we plan to recruit (6.4%, Ussher 2015; 7.6%, Coleman 2012). The average control group abstinence rate was 7.5%. Our estimate of 14% in the intervention group, is based on both the cessation rate in our Glasgow pilot (22.5%, Tappin 2015), plus a consideration of what would be considered clinically important. We have conservatively estimated 14% to allow for differences in the populations and cessation services in study regions compared with Glasgow. A gain of 7% to 14% would be considered clinically important and is similar to pharmaceutical aids in non-pregnant smokers (Cahil 2013). We therefore consider a clinically significant improvement will be a doubling of the smoking cessation rate.

Blocked random allocation will ensure a balanced number of participants in both groups. There is no advantage to stratified randomisation as the sample size is large even for each site (Hewitt and Torgerson, 2006) With sample sizes of greater than 100 at each site (approximately 200 per site) co-variates do not generally need to be stratified for as simple random allocation with covariate adjustment in the analysis is just as efficient as stratified randomisation but with less risk of allocation bias due to prediction of the allocation sequence (Hewitt and Torgerson 2006).

15.2 Main Analysis

15.2.1 1 Primary outcome analysis

Primary outcome analysis will be by intention to treat as the intervention is the offer of a financial incentive to engage with cessation services and quit smoking. The main analysis will use logistic regression adjusting for prognostic variables – age, years of smoking, deprivation/income status and levelof smoking, and centre.

The primary outcome will be supplemented by cotinine measurement on residual routine late pregnancy blood samples for those women who gave consent for their residual routine blood samples to be used for research purposes. This supplementary measure will be utilised for participants either not contactable in late pregnancy or who self report as quit when contacted but are not available to provide a urine and saliva sample for cotinine. This measure will also be used to assess gaming.

15.2.2 2 Secondary outcome analysis

Engagement with SSS and self-reported smoking status at 4-weeks post quit will both be analysed using a logistic regression modeladjusting forthe same co-variates as the primary analysis.

Analysis of birth weight will use regression methods. Treatment groups will be compared adjusting for key prognostic variables including age, centre, height and weight of mother at early pregnancy. The intention to treat estimate will be severely diluted due to low smoking cessation rates and the ‘perprotocol’ analysis will be biased by confounding. We will utilise an instrumentalvariable

approach – Complier Average Causal Effect analysis – which will estimate the true impact of incentive inducted smoking cessation on birth weight (McConnachie 2017).

Continuous abstinence outcomes obtained postpartum (West 2005) will be calculated using logistic regression adjusting for the same covariates (age, centre, height and weight of mother at booking), excluding women who were followed up less than six months postpartum. We will explore tests of interaction between age of mother and outcome; deprivation score, years and levelof smoking. Effects on length of neonatalunit stays will be examined.

Differences by subgroup (e.g. site, deprivation, age group) will be explored and reported as per the CHAMP guidelines. A meta-analysis including data collected in the feasibility study in Glasgow on 612 participants will be undertaken.

15.2.3 Other outcome analysis

Point abstinence outcomes obtained at six months postpartum (i.e. regardless of whether participants were abstinent in late pregnancy) will be calculated and analysed using logistic regression adjusting for the same covariates (age, centre, height and weight of mother at booking). We will explore tests of interaction between age of mother and outcome; deprivation score, years and levelof smoking. Effects on length of neonatal unit stays will be examined.

Data from feasibility trial centre in Glasgow analysed in priori meta-analysis.

15.2.4 Missing data

Where there is missing data for the primary outcome (i.e. cessation of smoking) we will assume that women are continuing to smoke. This assumption will be examined by retesting residual blood samples taken for other reasons in late pregnancy for cotinine as in the single centre feasibility trial (Tappin 2015). This assumption will also apply to the secondary outcome cessation six month post delivery. Other secondary outcomes, e.g. birth weight, are collected routinely and will have little missing data. Long term outcome data collection will be planned from participants and offspring to inform additional follow-up studies.

15.2.5 Sensitivity analyses

A sensitivity analysis including the women who were followed up at less than six months postpartum will be carried out for the six months postpartum abstinence outcomes.

15.3 Health economics analysis

The cost-effectiveness analysis for offering financial incentives in addition to routine smoking cessation services, will use a health service perspective and adhere to best practice guidelines (Husereau 2013, NICE 2013). Resource use and primary outcome data (adjusted cessation at 34-38 weeks gestation) will be reported as incremental cost per quitter. Multiple imputation will account for missing data, assuming data are missing at random, so that missingness can be predicted by other complete cases. A longer term analysis will incorporate additional costs for low birth weight and otherbirth outcomes, and account forsmoking relapse post partum (using the 6 months

postpartum trial data). Lifetime analysis will adapt a published probabilistic decision analytic model (Bauld 2009, Bauld 2011) to examine the incremental cost per QALY gained, presenting confidence limits around cost and QALY outcomes. Extensive sensitivity analysis will include (i) re-analysis to account for gaming identified (ii) self-reported outcomes, (iii) additional cost for smoking related disease.

16.0 REGULATORY CONSIDERATIONS

16.1 Ethical and research governance approval

We will seek ethics approval through the NHS Research Ethics Committee before the trial commences. The trial will also be reviewed by the University of York's Health Sciences Research Governance Committee.

Before any site can enrol participants in the trial the site must have provided local NHS R&D approval for the study.

The trial will be conducted to protect the human rights and dignity of the participant as reflected in the 1996 version of the Helsinki Declaration.

16.2 Informed consent

Verbal telephone consent will be used in this trial as the main consent process. This is important so that this intervention remains pragmatic and integrated into current SSS at all of the study sites. Introducing written consent would make this trial very much more unwieldy and less generalisable as the proportion of women who agreed to take part and their socio-economic status would likely move away from being representative.

The large feasibility trial which enrolled 612 pregnant smokers in Glasgow (Tappin 2015) used verbal consent procedures as in the current trial. No problems occurred during the trial or since related to this form of consent. All consent calls were audio-recorded which will again be the case for this current trial. The participants were sent a copy of the consent form so that they had a written record of the consent process and when it took place. The trial population was shown to be representative of all pregnant smokers in the area covered by the pilot trial (Bessing 2016).

With sites in three of the four UK nations, travel for the qualitative researcher to Site C, Wessex and Site D will be limited. Travel to the homes of eligible women will compound this difficulty. We propose that the qualitative research consent process also uses verbal telephone consent, audio-recorded by the qualitative researcher after being given permission to record the consent process by the eligible pregnant lady. Qualitative research is commonly audio-recorded whether conducted face- to-face oroverthe phone.

The NHS Health Research Authority published guidance in early 2017 regarding consent being proportionate to the risks associated with a trial as far as participant safety and also rights. We hope that the ethics committee will agree with our view that the consent procedures we propose to employ adheres to the thrust of that guidance (http://www.hra.nhs.uk/news/2017/01/31/hra-publishes-new-proportionate-consent-guidance/).

During routine 1st telephone contact by the NHS SSS to each pregnant smoker referred by NHS Maternity Services, verbal permission will be sought by SSS staff from eligible smokers for their contact details to be passed from NHS services to the Trial Team. Those who agree to this transfer to the Trial Team will be sent a PIS about the trial prior to telephone 'consent' contact by the TCC. Verbalpermission for transfer of this information to the Trial Team was approved by the Caldicott

Guardian for the feasibility trial in Glasgow (Tappin, 2015) and will be obtained for all centres for this trial.

The TCC will make a 'consent' contact call to eligible smokers who have given verbal permission for their contact details to be passed. The trial will be discussed including the number of contacts made and that related pregnancy data will be made available to the Trial Team including gestation at birth and birth weight. The clients will also be asked if their residual routinely collected blood samples can be used for research purposes. The importance of follow-up contact will be stressed and the ways to make contact including landline, mobile phone, text messaging, individual Facebook contact, and letter, will be discussed and agreed. Eligible clients will then be asked to consent to the incentives trial during this first telephone contact with the TCC. Consent will also be obtained during this call for a researcher to contact them and ask some questions about their experience of taking part, as well as permission to audio-record this discussion.

A copy of the consent form signed by the TCC will be sent to the participant with an electronic copy kept by the trial team. The consent call will be audio-recorded as is routine Contact Centre policy and this recording will be kept as a record of consent to take part in the trial.

16.3 Confidentiality of data and patient records

Data will be handled in accordance with the Data Protection Act 1998.

Trial data including participants contact details will be stored on the trial database hosted by ECHO Managed Services. The data will be accessed by the Trial Team including The Call Centre via a secure web portal. The same web portal was approved by the NHS Greater Glasgow and Clyde IT security advisor for the pilot trial in Glasgow, who defined security systems for the portal that was in keeping with NHS security standards. When all trial data has been entered onto the database a download of the data without direct identifiers will be made available to York Trials Unit to enable them to perform the data analysis.

For the qualitative study, all personal identifiers will be removed from interview transcripts and observation field notes.

Data from labs will be sent to the central Trial Team in Glasgow and entered through the trial portal onto the trial database.

Electronic data on participants will be stored in accordance with ICH GCP guidelines by Glasgow University and at the York Trials Unit, University of York, until the youngest child is 21 years old.

16.4 Potential risks and benefits

There are few risks to participants. The only appreciable risk is related to the disclosure of identifiable data. The trial data will be held in a database hosted by ECHO Managed Services Ltd and will be used confidentially by the Trial Team including the TCC hosted by ECHO who will be taking patient consent. The data will not be transferred to anyone outside the immediate Trial Team.

Care will be taken not to disclose name or contact details of participants in the intervention group to those in the control group in case of any hostility arising. However, hostility was not seen in the CPIT phase II feasibility study in Glasgow, in fact those interviewed for qualitative purposes in CPIT II understood the importance of having a control group to make sure that giving incentives was effective (Tappin 2015).

All participant data used by the Trial Team and the NHS SSS will be kept strictly confidential, in accordance with NHS procedures, and in accordance with the Data Protection Act 1998.

For the qualitative element of the study, it is possible that participants may discuss complex issues relating to their smoking patterns and attitudes towards smoking. Measures will be in place to minimise any discomfort in discussing their smoking by stating prior to interviews that participants can abandon the interview at any time. If a participant wishes to withdraw, we shall suggest to the participant a follow up call at a later date to ensure that they are alright and to see if they require any further support (e.g. from NHS SSS).

Participants who agree to their residual routinely collected blood samples being used for research purposes at the time of consent will be notified that this will be held at NHSGG&C Bio-repository which has been ethically approved as a research biobank with an approved authorisation process for the use of surplus tissue for research purposes or local secure storage facilities at each centre. If these participants are not contactable in late pregnancy, residual routine blood samples taken in late pregnancy will be retrieved and sent for cotinine testing to provide a supplementary measure of the primary outcome – smoking in late pregnancy (Tappin, 2015).

16.5 Indemnity

NHS GG&C will provide indemnity and compensation in the event of a claim by, or on behalf of participants, for negligent harm as a result of the management of the research. University of Glasgow will provide indemnity and compensation in the event of a claim by, or on behalf of participants for negligent harm as a result of the study design and/or in respect of the protocol authors/research team. Both University of Glasgow and NHS GG&C will provide indemnity with regards to the conduct of the research. The University of Glasgow has in force a Professional Indemnity and/or Clinical Trials Policy which provides cover for negligent harm and the activities here are within that coverage.

17.0 TRIAL OVERSIGHT

17.1 Trial Management Group (TMG)

The trial manager (Lesley Sinclair) will lead this group supported by the Investigators (primarily Professor Tappin). Each site will have a lead who will be a member of the TMG. York Trials Unit will support Lesley Sinclair in all aspects of trial management. This will include a data manager who will be responsible for working with ECHO Managed Services Ltd, a trial manager and local site leads to update the constructed database used for CPIT II (Tappin 2015) and establish systems of data collection and data checks as well as data monitoring. Jennifer McKell, the main researcher on the process evaluation aspect of the trial, will be a member of the TMG. The main researcher conducting the health economic analysis will also be a member of the TMG. The TMG will generally meet on a monthly basis but more frequently orless frequently as the trial requires.

17.2 Trial Steering Committee (TSC)

The TMG will be supported by the Trial Steering Committee (TSC).
The TSC will be made up of an independent chair, Dr Felix Naughton, the Chief Investigator, main statistician, trial management support, representatives from the major funding bodies (CRUK and CSO) , a patient representative and an international scientist with interests in smoking cessation during pregnancy.

Members of the TSC have provided input and advice on the trial design, will assist with the developmentof the study protocol and will monitor and provide overall supervision for the trial. The TSC will meet every six months but more often if required.

Memberswillbe required to sign up to the remit and conditions as set out in the TSC charter.

17.3 Data Monitoring Committee (DMC)

The TSC will take on the role of the DMC. As this is a low risk 'open' study a separate DMC, which would have a role in looking at unblinded data, is, we believe, not necessary. Instead, we propose that independent members of the TSC meet separately with the study statistician, to discuss emerging data with a particular reference to any issues affecting participant welfare. We cannot envisage and did not see any AEs in the large feasibility trial in Glasgow (Tappin 2015).

18.0 USER AND PUBLIC INVOLVEMENT

This study has been informed by the involvement of two lay advisors. Both have commented on the design and have agreed to serve as advisors forthe trial.

19.0 PUBLICATIONS

The study protocol and results will be reported and disseminated in a peer reviewed journal for publication. The trial results, whether negative or positive, will be disseminated using peer reviewed publications and presentations. The final trial paper will be submitted to a peer-reviewed journal within 12 months of the results being available to the study team. The co-investigators will also be available as a resource on 'how financial incentives were delivered' within NHS SSS. The anonymised dataset will be available to external researchers three years after the trial end via the lead investigators.

Care will be taken to ensure no participant is identified on any publications. The names of participants will not be used when publishing quotes from qualitative research.

We will produce a short summary of the resultsof the study which can be distributed to trial participants and participating sites.

20.0 RETENTION OF TRIAL DOCUMENTS

Essential trial documentation (i.e. documents which individually and collectively permit evaluation of the conduct of the trial and the quality of the data produced) will be kept with the Trial Master File and Investigator Site Files. The documents will be retained for a minimum of seven years after completion of the trial in order to comply with standards of Good Clinical Practice. All electronic records will be stored on a password protected server.

The digital voice recordings, obtained for the process evaluation research, will be destroyed at the end of the study. The anonymised transcripts will be retained in a secure archive setting for 5 years to facilitate future analysis and publication of the study material.

21.0 STUDY CLOSURE/DEFINITION OF END OF TRIAL

The study will end when the TSC agrees that one or more of the following situations applies:

- Last patient last study visit;

OR

i. The planned sample size has been achieved;
ii. The Independent Trial Steering Committee (TSC) has advised discontinuation, e.g. because of safety concerns about the trial, or a statistically significant difference in clinical outcomes is evident between the two treatment arms;
iii. There is insufficient funding to support further recruitment, and no reasonable prospect of additional support being obtained;
iv. New information makes it inappropriate to continue to randomise patients to one or other arm of the trial;
v. Recruitment is so poor that completion of the trial cannot reasonably be anticipated

REFERENCES

Bauld L, Briggs A, Boyd K, Chesterman J, Ferguson J, Judge K. Comparing models of smoking treatment in Glasgow. Final report. 2009; Final report.

Bauld L, Boyd KA, Briggs AH, Chesterman J, Ferguson J, Judge K, Hiscock R. Oneyear outcomes and a cost-effectiveness analysis for smokers accessing group-based and pharmacy-led cessation services. Nicotine Tob Res 2011;13(2):135-45.

Bessing B, Bauld B, Sinclair L, Mackay DF, Spence W, Tappin DM. Representativeness of the participants in the smoking Cessation in Pregnancy Incentives Trial (CPIT): A cross-sectional study. Trials 2016; 17(1):426. DOI: 10.1186/s13063-016-1552-5

Boyd KA, Briggs AH, Bauld L, Sinclair L, Tappin D. Are financial incentives cost-effective to support smokingcessation during pregnancy? 2016; Addiction; 111(2): 360-70. doi: 10.1111/add.13160

Cahill K, Stevens S, Perera R, Lancaster T. Pharmacological interventions for smoking cessation: an overview and network meta-analysis. Cochrane Database Syst Rev. 2013 May 31;(5):CD009329. doi: 10.1002/14651858.CD009329.pub2.

Coleman T, Cooper S, Thornton JG, Grainge MJ, Watts K, Britton J, Lewis S. A randomized trial of nicotine-replacement therapy patches in pregnancy. N Engl J Med 2012;366(9):808-18.

Doll R, Peto R, Boreham J, Sutherland I. Mortality in relation to smoking: 50 years' observations onmale British doctors. BMJ 2004;328:1519Hewitt CE, Torgerson DJ. Is restricted randomisation necessary? BMJ 2006; 332 doi: https://doi.org/10.1136/bmj.332.7556.1506

Donovan JL, Rooshenas L, Jepson M, Elliott D, Wade J, Avery K, Mills N, Wilson C, Paramasivan S, Blazeby JM. Optimising recruitment and informed consent in randomised controlled trials: the development and implementation of the Quintet Recruitment Intervention (QRI). Trials (2016) 17: 283. doi:10.1186/s13063-016-1391-4

Hoddinott P, Britten J, Pill R. Why do interventions work in some places and not others: a breastfeeding support group trial. Soc Sci Med 2010;70(5):769-78.

Husereau D, Drummond M, Petrou S, Carswell C, Moher D, Greenberg D, Augustovski F, Briggs AH, Mauskopf J, Loder E. Consolidated health economic evaluation reporting standards (CHEERS) — Explanation and elaboration: A report of the ISPOR health economic evaluations publication guidelines good reporting practices task force. Value Health 2013; 16:231-50

Leonardi-Bee J, Jere ML, Britton J. Exposure to parental and sibling smoking and the risk of smoking uptake in childhood and adolsecence: a systematic review and metaanalysis. Thorax 2011;66:847-55 doi:10.1136/thx.2010.153379

McConnachie A, Haig C, Sinclair L, Bauld L, Tappin D. Birth weight differences betweenthose offered financial voucher incentives for verified smoking cessation and control participants enrolled in the Cessation in Pregnancy Incentives Trial (CPIT), an intuitive approach and a Complier Average Causal Effects (CACE) analysis. Trials 2017, 18 (1), Art. No.:337

Moore GF, Audrey S, Barker M, Bond L, Bonell C, Hardeman W, Moore L, O'Cathain A, Tinati T, Wight D, Baid J. Process evaluation of complex interventions: Medical Research Council guidance BMJ 2015; 350 :h1258

Morgan H, Hoddinott P, Thomson G, Crossland N, Farrar S, Yi D, Hislop J, Moran VH, MacLennan G, Dombrowski SU, Rothnie K, Stewart F, Bauld L, Ludbrook A, Dykes F, Sniehotta FF, Tappin D, Campbell M. Benefits of Incentives for Breastfeeding and Smoking cessation in pregnancy (BIBS): a mixed-methods study to inform trial design. Health Technol Assess. 2015 Apr;19(30):1-522, vii-viii. doi: 10.3310/hta19300.

NICE (National Institute for Health and Care Excellence).Guide to the methods of technology appraisal 2013; Process and Methods Guides, 2013

NICE (National Institute for Health and Care Excellence). Smoking: stopping in pregnancy and after childbirth. 2010; Public Health Guidance 26. https://www.nice.org.uk/guidance/ph26

Parkin DM, Boyd L, Walker LC. The fraction of cancer attributable to lifestyle and environmentalfactors in the UK in 2010. Br J Cancer 2011;105:S77-81.

Portanti M, Whitworth S. A comparison of the characteristics of childlesswomen and mothers in the ONS Longitudinal Study. Office for National Statistics, Population Trends 2009; 136: 10-20.

Tappin D, Bauld L, Purves D, Boyd K, Sinclair L, MacAskill S, McKell J, Friel B, McConnachie A, De Caestecker L, Tannahill C, Radley A, Coleman T. Financial incentives for smoking cessation in pregnancy: Randomised controlled trial. BMJ 2015; 350: h134.

Tappin DM, MacAskill S, Bauld L, Eadie D, Shipton D, Galbraith L. Smoking prevalence and smoking cessation services for pregnant women in Scotland. Subst Abuse Treat Prev Policy 2010;5:1.

Ussher M, Lewis S, Aveyard P, Manyonda I, West R, Lewis B, Marcus B, Riaz M, Taylor A, Daley A, Coleman T. Physical activity for smoking cessation in pregnancy: randomised controlled trial. BMJ 2015;350:h2145.

West R, Hajek P, Stead L, Stapleton J. Outcome criteria in smoking cessation trials: proposal for a common standard. Addiction 2005 ;100(3):299-303.

Yin, R. K. (2013). Case study research. Design and methods. 5th edition. London: Sage

Appendix 1 – Study Timelines

or this current CPIT III trial, enrolment was planned for 18 months from Dec'18 - May'19. Pr ofessor Torgerson York Trials Unit indicated that should enrolment be slower, recruitment could o tinue until Dec'19. The 3 month funded extension agreed by CRUK allows recruitment to March 2020. By January 2020, we had recruited 879 participants (36 per month) leaving 61 to be recruited in February & March 2020.

Follow-up: All participants will be followed up in late pregnancy at the primary outcome point as planned. The drawback with the extended recruitment period was that the secondary outcome – smoking six months after birth - would not be available for participants enrolled after Jun'19. This back up plan was presented to the CRUK funding committee prior to permission to start. Most relapse after the end of pregnancy is immediate. We are therefore now going to follow-up 70% of participants six months after birth and also the last 30% of participants some time in the first six months after birth. This will give a strong measure of relapse by six months after birth as planned, but will also give information about when relapse takes place after birth in this cohort of pregnant smokers.

No-cost extension: A no-cost extension was granted by CRUK and CSO until 30th November 2021. The extension was requested to mitigate issues resulting from the COVID-19 pandemic, (1) the return of participant samples to the research team which were collected pre-COVID but were stuck at participating Wessex sites due to the pandemic, and (2) to allow the distribution of the online questionnaire. An additional no-cost extension was granted by CRUK until 31st July 2022 to complete relevant trial close-down procedures.

Appendix 2 – Proposal for CPIT III follow-up during COVID-19 pandemic

PURPOSE OF THIS DOCUMENT

We are requesting an exemption for CPIT III from the suspension of routine follow up-visits for all GG&C hosted clinical trials during the COVID-19 pandemic. We present below two options for the Sponsor to consider that would allow CPIT III participant follow-up to continue at this time. Both options involve remote contactonly with participants by non-clinical University research staff not currently redirected elsewhere.

PROPOSAL FOR CPIT III FOLLOW-UP DURING COVID-19 PANDEMIC

900+ participants (target 940) are enrolled in CPIT III. To date primary and secondary outcome data has been collected for 75% and 48% of these participants respectively. The current protocol (A) has five follow-up points for women recruited to the trial with follow-up scheduled to continue for seven months until Oct-20. Details (i.e. who, what, when) of the follow-up time points together with two alternative follow-up protocols (B and C) in response to current circumstances are presented in Table 1 below. Both these alternate schedules adhere to current guidelines/advice regarding COVID-19 (this will be reviewed on a daily basis) whilst removing the need for input from study nurses at the recruiting sites who will be deployed elsewhere within the NHS during the pandemic. Both protocols allow completion of follow-up without the need to approach funders for an extension at a later date (likely difficult given the charitable status of funding bodies involved) and importantly will deliver shopping voucher incentives promised to participants at a time when household income is likely to be compromised. In addition, both protocols can be conducted over the telephone from home by non-clinical University research staff at the Universities of Glasgow and Edinburgh and Site C who are not currently being redirected to work elsewhere.

Furtherdetails of follow-up arrangements for allthree protocols are given in Table 1 below. In summary:

Protocol A, the current approved protocol, involves substantial input from NHS study nurses and home visits to participants who self-report as having stopped smoking. A Carbon Monoxide (CO) breath test is conducted at all visits and a saliva/urine sample collected at primary and secondary outcome points. Participants receive shopping voucher incentives based on CO breath test results/acquisition of saliva/urine samples at home visits.

Protocol B involves remote collection of approx. 90 saliva samples from self-reported smoke-free participants over the next seven months. No samples will be collected from any participants reporting COVID-19 related symptoms. Sample collection kits will be sent out and participants guided through collection over the phone by University researchers. Samples will be returned by Royal Mail to NHS Site D R&D and stored in freezers by staff working on COVID- 19 studies. This has been agreed by Berni Welsh NHS R&D Site D. These samples will be stored until ABS Laboratories Ltd confirm capacity to analyse

at a future date. No CO breath tests will be conducted. Shopping voucher incentives will be sent out based on verbal self -reported smoking status and receiptof saliva samples forthose who self-report as smoke free at primary and secondary outcome stages.

Protocol C involves telephone contact only with participants by University researchers. Neither CO breath tests nor remote collection of saliva samples will be conducted. Shopping voucherincentives will be based on verbalself-report of smoking status.

We would like to engage immediately with protocol B moving to protocol C, if necessary, should circumstances change further or additional restrictions be applied. Protocol B allows us to retain the validity of the primary and secondary outcomes collected at follow-up 4 and 5 (see table 1). Protocol C will reduce the validity of the trial outcomes for the last 250 participants recruited to the trial but will allow participants to receive promised shopping vouchers.

Table 1. Current protocol and proposed alternative COVID-19 follow-up arrangements

**Follow-up**	**A. Current protocol arrangements**	**B. Proposed COVID-19 arrangements to be implemented now**	**C. Proposed COVID-19 arrangements to be implemented if further restrictions applied**<br><br>*(i.e. Saliva samples unable to be returned by Royal Mail/other couriers, NHSL unable to receive saliva samples, ECHO Managed Services Ltd unable to complete initial stages of primary and secondary outcome follow-up)*
1. Stop Smoking Services (SSS) Engagement	CPIT study nurses at each site liaise with Stop Smoking Services (SSS) to ascertain uptake of stop smoking behavioural support and obtain each participant's agreed date to stop smoking (quit date).	CPIT University research staff will assume this role.<br><br>This will need to be completed for up to 20 participants in the next eight weeks.	CPIT University research staff will assume this role.<br><br>This will need to be completed for up to 20 participants in the next eight weeks.

2. 4-weeks after agreed quit date	CPIT study nurses at each site liaise with SSS/participants to obtain self-reported smoking status four weeks after the agreed quit date and obtain a Carbon Monoxide (CO) breath test from those participants in the intervention group who self-report as having stopped smoking.	CPIT University research staff will liaise with SSS/participants at each site by telephone to obtain self-reported smoking status only.<br><br>No CO testing will be conducted in line with national guidance for SSS issued by the National Centre for Smoking Cessation Training (NCSCT) https://www.ncsct.co.uk/usr/pub/COVID-19%20bulletin%2018:03:20.pdf<br><br>This will need to be completed for up to 50 participants (including 20 highlighted above) in the next 12 weeks	CPIT University research staff will liaise with SSS/participants at each site by telephone to obtain self-reported smoking status only.<br><br>No CO testing will be conducted in line with national guidance for SSS issued by the National Centre for Smoking Cessation Training (NCSCT) https://www.ncsct.co.uk/usr/pub/COVID-19%20bulletin%2018:03:20.pdf<br><br>This will need to be completed for up to 50 participants (including 20 highlighted above) in the next 12 weeks
3. 12-weeks after agreed quit date	CPIT study nurses at each site liaise with SSS/participants who CO- validated as quit at 4-weeks to obtain self-reported smoking status at twelve weeks after the agreed quit date and obtain a CO breath test from those participants in the intervention group who self-report as having stopped smoking	As per 4-week follow-up above.<br><br>This will need to be completed for up to 70 participants (including 50 highlighted above) in the next six months	As per 4-week follow-up above.<br><br>This will need to be completed for up to 70 participants (including 50 highlighted above) in the next six months
4. Primary Outcome: Late pregnancy (34-38 weeks gestation)	ECHO Managed Services Ltd (ECHO) contact all participants by telephone to ascertain self-reported smoking status and collect other health economic data. No further follow-up is required for	ECHO will continue to telephone participants to ascertain self-reported smoking status and collect other health economic data. No further	CPIT university research staff will telephone participants to obtain self-reported smoking status and collect other health economic related

	those participants who self-report as smoking. CPIT study nurses at each site attempt to contact those participants that ECHO cannot contact to ascertain smoking status. CPIT study nurses visit all participants who self-report as having stopped smoking to obtain a CO breath test result and a saliva/urine sample which they process and store at local labs and arrange onward transportation to ABS Laboratories Ltd, York (ABS).	follow-up will be required for those participants who self-report as smoking.<br><br>CPIT University research staff will attempt to contact those participants that ECHO cannot contact to ascertain smoking status. No CO testing will be conducted.<br><br>This will need to be completed for up to 250 participants (including all previously highlighted participants).<br><br>Those who self-report as having stopped smoking, estimated to be approx. 40 participants (based on the quit rate of 18% from the CPIT feasibility study https://www.bmj.com/content/350/bmj.h134) will have a return freepost saliva collection kit posted to their home and will be telephoned by University research staff to guide them through sample collection. On receipt of saliva sample self-report quitters will be sent their shopping voucher incentives.	data. No CO or saliva sample collection will be conducted.<br><br>This will need to be completed for up to 250 participants (including all previously highlighted participants).<br><br>Shopping voucher incentives will be sent once self-reported smoking status is ascertained

		Saliva samples received by post will be stored in study freezer within NHSL R&D for transportation to ABS for analysis at a future date.	
5. Secondary Outcome: Six months post-partum	As per primary outcome follow-up above.	ECHO will continue to contact participants to ascertain self-reported smoking status and collect other health economic related data. No further follow-up will be required for those participants who self-report as smoking.<br><br>CPIT University research staff will attempt to contact those participants that ECHO cannot contact to ascertain smoking status. No CO testing will be conducted.<br><br>This stage will need to be completed for up to 500 participants (including all previously highlighted participants). Those who self-report as quit estimated to be approx. 50 participants (based on a quit rate of 10% from the CPIT feasibility study https://www.bmj.com/content/350/bmj.h134) will have a return freepost saliva collection kit posted to their home and will be telephoned by University research staff to guide them through sample collection. On receipt of saliva sample	CPIT University research staff will telephone participants to obtain self-reported smoking status and collect other health economic related data. No CO testing or saliva sample collection will be conducted.<br><br>This stage will need to be completed for up to 500 participants (including all previously highlighted participants).<br><br>Shopping voucher incentives will be sent once self-reported smoking status is ascertained.

		self-report quitters will be sent their shopping voucher incentives.<br><br>Saliva samples received by post will be stored in study freezer within NHSL R&D for transportation to ABS for analysis at a future date.	

Shopping voucher incentives are dispatched to participants in the intervention group at each stage of the study on entry of the required data to the study database. Distribution of all shopping vouchers is via Royal Mail and is handled by a private fulfilment house who are continuing to operate at present.

1. Summary of changes to protocol

AM No.	AM Type & No.	AM Date	Purpose of Amendment
N/A	N/A	16/08/2017	Original REC submission
AM01	NSA	14/09/2017	Change of PI in Site D from D Tappin and L Bauld to Shirley Mitchell prior to initial approval
AM02	SA01	01/11/2017	Changes to content of main consent form, main PIS and protocol. No changes to data collected, safety of participants or trial procedures
AM03	NSA	05/01/2018	Changes to PIS: Formatting and layout. Also made clear that £100 voucher at 3 months only available if quit smoking at 4 weeks<br>Change to Consent Form: Paragraph 2 - changed 'treatment' to 'support'
AM04	NSA	24/05/2018	Change to protocol regarding clarification of CO cut off and vouchers new text: 'Incentive payments at 4 and 12 weeks post-quit date (see 10.2.2 and 10.2.3) and 34-38 weeks gestation are based on the SSS accepted CO levels for a non-smoker at each site. In routine practice an element of discretion is exercised around these CO levels to allow for passive smoking. The trial will thus adhere to the same principles and consideration will be given to individual cases (agreed with sponsor).' page 30
AM05	NSA	21/06/2016	AM05 was an "administrative clarification to protocol" dated 21 June 2016 (year looks incorrect) processed by the HRA, which the REC was not informed about that is related to AM04 - Change to protocol regarding clarification of CO cut off levels for incentive payments
AM06	SA02	10/07/2018	See AM06 Full Details tab Appendix 1 for details of the amendment.
AM07	NSA	28/09/2018	GDPR compliance changes and addition of new site (Site A) (Adding a site is a NSA - see 31/07/2018 e-mails from Louise Bell)
AM08	NSA	10/01/2019	Addition of new sites
AM09	NSA	19/03/2019	Addition of new site - Site G
N/A	Administrative change included in AM11	26/07/2029	CPIT Central Office address change
AM10	NSA Dec 2019	05/12/2019	3-month funded extension

AM11	SA03	05/03/2020	Changes to timing of planned post-partum follow-up<br>Addition of sensitivity analyses<br>Change of PI at Site C<br>Addition of new sites (AM07 28/09/2019, NSA Jan 2019, NSA Mar 2019)<br>Inclusion of text re adherence with intervention<br>Incorporation of 3-month funded extension to timelines (NSA Dec 2019)<br>Inclusion of secondary objective/outcome measure previous omitted in error<br>Updates to trial reference numbers (cover page), protocol amendment history (p3), contact details (cover page, section 1.0), funding sources (cover page, Study synopsis), list of abbreviations and TSC to reflect all previous approved SA's and NSA's<br>Revised improved version of study visits flow chart : 2.2 Schedule Of Assessment and Data Collection for Trial Outcomes<br>CPIT Central Office address change<br>Other minor corrections to and formatting of text<br>Full details shown in separate tab/protocol
AM12	NSA	30/03/2020	Exception from suspension of GG&C hosted clinical trials due to Covid-19. Face-to-face contact between NHS research staff and participants replaced with remote contact by university research staff and participants to continue data collection.<br>Full details shown in separate tab
AM13	SA04	18/12/2020	Addition of online HCP survey for Process Evaluation
AM14	NSA	24/03/2021	Revised HP Survey & no-cost extension
AM15	NSA	03/02/2022	Timeline update to reflect further no-cost extension to 31/07/2022 and update to sponsor rep and LS contact details
AM16	NSA	10/03/2022	Minor changes to study documents

Appendix B: Key characteristics of CPIT III trial sites

Appendix B: Key characteristics of CPIT III trial sites

(Characteristics presented for sites A to E were recorded within case studies conducted as part of an embedded process evaluation and reported in: McKell J; Harris F M; Tappin D; Bauld L; Hoddinott P. *Usual care in a multi-centre randomised controlled trial of financial incentives for smoking cessation in pregnancy: qualitative findings from a mixed methods process evaluation.* Submitted to BMJ Open 10/07/2022 (reference bmjopen-2022-066494). Characteristics presented for Sites F and G were recorded in notes of Trial Management Working Group meetings by a process evaluation researcher)

	Site A	Site B	Site C	Site D	Site E	Site F+	Site G+
Policy of Referral (Opt-in or Opt-out)	Opt-out	Opt-out	Opt-out	Opt-out	Opt-out	Opt-out	Opt-out
Target population and behaviour change supported	Pregnant women, smoking and weight management	Pregnant women, smoking only	Pregnant women, smoking only	General population, smoking only	General population, smoking, alcohol, weight management and physical activity	Pregnant women, smoking only	Not collected
Organisation providing SSS*	NHS	NHS	NHS	NHS	Local Government	Local Government	NHS
SSS advisers with or without a midwifery/nursing background	Mixed – some with and some without a midwifery/ nursing background	Midwifery/ nursing background	Midwifery/ nursing background	Midwifery/nursing background	No midwifery/nursing background	No midwifery/nursing background	Midwifery/nursing background
SSS funder (and location of line management)	Local Government (NHS Trust/Board)	NHS Trust/Board (NHS Trust/Board)	Public Health Body (NHS Health Improvement)	NHS Health Improvement (NHS Health Improvement)	Local Government (Local Government)	Local Government (Local Government)	Not collected
Venue for SSS consultations	Hospital consultation room or other	Hospital consultation room	Hospital consultation	Community venue (regular drop-in or	Community venue (regular drop-in	Regular drop-in centre	Not collected

	space (not dedicated). Also, a GP Surgery room for those remotely located	(dedicated) or home visit	room (dedicated)	by appointment) or home visit	or by appointment)		
Methods of SSS consultation	Face to face or telephone	Face to face or telephone	Face to face or telephone	Face to face or telephone	Face to face or telephone	Face to face or telephone	Not collected
SSS ability to provide NRT directly	No	Yes	No	Yes	No	Given a slip to take to pharmacy	Not collected
Co-location of SSS and Maternity Services	Yes	Yes	Yes	No (in most cases)	No	Not collected	Not collected

* SSS has been used to denote Stop Smoking Services within trial sites, though some also provided support for other behaviour change and service titles reflected this.

+ Sites F and G were not included as case studies as part of the process evaluation and information about these is limited.

Appendix C: Statistical Analysis Plan with list of changes made

CPIT III

The Smoking Cessation in Pregnancy Incentives Trial: A Phase III Randomised Controlled Trial

STATISTICAL ANALYSIS PLAN

v1.1

York Trials Unit	Version date: 28/07/21
Department of Health Sciences	Authors: Alex Mitchell & Ada Keding
University of York	Chief Investigators: David Tappin & Linda Bauld
York, YO10 5DD	Trial Managers: Lesley Sinclair & Lyn Robinson-Smith

Contents

1 General 1
1.1 Document Scope 1
1.2 Glossary 1
1.3 Procedural Documentation 1
1.3.1 Standard Operating Procedures 1
1.3.2 Associated Documentation 1
2 Trial Summary 2
2.1 Objectives 2
2.1.1 Primary Objective 2
2.1.2 Secondary Objectives 2
2.2 Design 2
2.3 Intervention 2
2.3.1 Original intervention 2
2.3.2 Changes to the intervention in response to the Covid-19 pandemic 3
2.4 Usual care 4
2.5 Outcomes 4
2.5.1 Primary Outcome 4
2.5.2 Secondary Outcomes 5
2.5.3 Adverse events 6
2.5.4 Other Collected Data 6
2.6 Sample size 7
2.7 Randomisation 7
2.8 Blinding 7
2.9 Follow-up 7
2.9.1 Follow-up 1: SSS Engagement 7
2.9.2 Follow-up 2: Four weeks post quit date 8
2.9.3 Follow-up 3: 12 weeks post quit date 8
2.9.4 Follow-up 4: Late pregnancy (34-38 weeks gestation) 8
2.9.5 Follow-up 5: Six months postpartum 9
3 Study Data 10
3.1 Trial Data 10
3.2 External datasets 10
3.3 Management of Datasets and Data Verification 11
3.4 Location of Data and Associated Files 11

4 Analysis 11
4.1 Strategy for reporting data and general considerations 11
4.2 Recruitment and attrition 11
4.3 Baseline data 11
4.4 Primary outcome analysis 11
4.5 Secondary outcome analyses 12
4.6 Exploratory outcome analysis 13
4.7 Sensitivity analyses 13
4.7.1 Sensitivity of primary analysis to sparse data bias 13
4.7.2 Impact of the Covid-19 pandemic on analysis of the primary and secondary smoking outcomes 13
4.7.3 Subgroup analyses 13
4.7.4 Missing data analyses 14
4.7.5 Impact of participants who were followed up at postpartum earlier than the planned 6 months 14
4.7.6 Sensitivity of primary analysis to assumption regarding participants with missing anabasine test 14
4.8 Compliance with intervention 15
4.9 Gaming of the intervention 15
4.10 Cotinine and anabasine test results 15
4.11 Adverse events 15
4.12 Planned interim review and analyses 15
5 SAP amendment log 15
6 Signatures of approval 16
6.1 Contributions 16
6.2 Signatures 16
7 References 16
8 Appendices 18
8.1 Scoring of Fagerström Test for Nicotine Dependence 18
8.2 Trial flow diagram 19
8.3 CONSORT diagram 20
8.4 Example tables 21
8.4.1 Baseline characteristics 21

List of Figures

Figure 1: A trial flow diagram detailing the flow of participants through the study and the incentives on offer to the intervention group. The shaded boxes indicate the trial team is not involved in this follow-up stage 19
Figure 2: A CONSORT diagram for CPIT III... 20

List of Tables

Table 1: CO threshold for each site 3
Table 2: Thresholds for cotinine samples in saliva 4
Table 3: CPIT III Data Collection Schedule 9
Table 4: Baseline characteristics for all participants and for participants who provided a cotinine/anabasine verified smoking status at the primary outcome follow-up. 21
Table 5: Primary and secondary analyses 22
Table 6: Comparison by treatment group of self-reported smoking status pre-Covid and post-Covid 22
Table 7: Comparison by treatment group of the number of participants who provided a positive biochemical sample, in the subset of participants who self-reported as non-smokers and provided a biochemical sample 23
Table 8: Comparison by treatment group of biochemically verified smoking status pre-Covid and post-Covid 23
Table 9: Comparison by treatment group of the number of participants who provided a biochemical sample, in the subset of participants who self-reported as non-smokers 23
Table 10: Anabasine results presented by treatment group and cotinine result for patients who reported as non-smokers and reported using e-cigarettes or NRT 24

1 General

1.1 Document Scope

This statistical analysis plan (SAP) covers the reporting of the trial progress and planned effectiveness analyses of the CPIT III trial. Analyses relating to health economic or qualitative data or any further exploratory post-hoc analyses are not covered by this SAP.

1.2 Glossary

AE	Adverse event
BMI	Body mass index
CACE	Complier average causal effect
CI	Chief Investigator
CO	Chemical symbol for carbon monoxide
CONSORT	Consolidated Standards of Reporting Trials
DMEC	Data Monitoring and Ethics Committee
EDD	Estimated Date of Delivery
FTND	Fagerström Test for Nicotine Dependence
IMD	Index of Multiple Deprivation
LSOA	Lower-layer Super Output Areas
NHS	National Health Service
NRT	Nicotine replacement therapy
PIS	Patient information sheet
ppm	Parts per million
REC	Research Ethics Committee
SAE	Serious adverse event
SQD	Set quit date
SOP	Standard operating procedure
SSS	Stop Smoking Service(s)
TCC	Trial Contact Centre
TMG	Trial Management Group
TSC	Trial Steering Committee
YTU	York Trials Unit

1.3 Procedural Documentation

1.3.1 Standard Operating Procedures

The following YTU SOPs and guidance documents will apply to the conduct and documentation of the CPIT III trial analysis:

S01	Statistical Considerations	Latest version: 5.0
SG02	Statistical Reporting Guidance	Latest version: 3.0

1.3.2 Associated Documentation

Appropriate YTU standard forms apply. Any assumptions made during the processing and merging of data as well as for the analysis will be documented (internal document reference numbers in bracket) using a Trial Assumptions Form (F23). In the event of necessary changes or additions to analyses detailed here, these will be documented on a Statistical Analysis Plan Departure Form (F24). The statistical analysis will be signed off using a Primary Analysis Sign-off Form (F16) and Statistical Quality Assurance Checklist (C03).

2 Trial Summary

The following sections give a summary of the CPIT III trial. Full details are given in the Study Protocol (latest version 4.0, dated 11/03/2020) and the published trial protocol paper [1].

2.1 Objectives

2.1.1 Primary Objective

The primary objective was to assess whether offering financial incentives in addition to usual Stop Smoking Service (SSS) support to pregnant smokers was effective in increasing the smoking cessation rate at late pregnancy.

2.1.2 Secondary Objectives

The secondary objectives were:

- To compare quit rates at four weeks post quit date between women offered incentives and those receiving usual SSS care only
- To compare quit rates at six months after birth (point abstinence) and until six months after birth (continuous abstinence) between women offered incentives and those receiving usual SSS care only
- To assess the cost effectiveness of financial incentives
- To identify the effects of differences in SSS, maternity care and demographic diversity of pregnant smokers on the effectiveness, cost effectiveness and transferability of financial voucher incentives

2.2 Design

CPIT III is a phase III, pragmatic, multi-centre, randomised controlled trial of the offer of financial incentives added to usual SSS care to engage with SSS and quit smoking versus usual care alone. In addition, economic and process evaluations were embedded in the study.

2.3 Intervention

2.3.1 Original intervention

The intervention was composed of several stages, each of which involved the offer of a financial incentive in the form of a Love2shop gift voucher, delivered via registered post.

The first stage of the intervention was designed to encourage participants to engage with SSS, while the remaining stages were designed to encourage participants to quit smoking and remain abstinent.

At the first stage, if the participant attended an appointment with the SSS and set a date on which they would quit smoking, they received a gift voucher with a value of £50.

At the second stage, the smoking status of those participants who engaged with SSS and set a quit date was obtained from the SSS at four weeks post-quit date. Smoking status was ascertained by asking the participant the question:

1. Have you smoked (even a puff) in the last two weeks?

Participants who answered ‘No’ were contacted by a research nurse to arrange an appointment to obtain a Carbon Monoxide (CO) breath test reading, where this had not already been collected by the SSS. Participants who had a CO reading less than or equal to the accepted threshold for their site received a gift voucher with a value of £50. Table 1 gives details of the CO threshold for each site.

Table 1: CO threshold for each site

Site	CO threshold (<=ppm)
Site D	4
Site C	3
Site A	4
Site B	4
Site F	5
Site G	4
Site E	4

At the third stage, the smoking status of those participants, who met the criteria to receive a shopping voucher at the second stage, was obtained from the SSS (where available) or by a research nurse at 12-weeks post quit date. Smoking status was obtained by asking the participant the question:

1. Have you smoked at all since your one-month follow-up?

Participants who answered 'No' were contacted by a research nurse to arrange an appointment to provide a CO breath test reading, where this had not already been collected by the SSS. Participants who had a CO reading less than or equal to the accepted threshold for their site received a gift voucher with a value of £100.

At the fourth stage, participants were contacted by the trial team at a random date between 34 and 38 weeks gestation, regardless of whether or not they met the criteria for the previous shopping vouchers. The participants were asked the following questions:

1. Have you smoked at all in the last eight weeks?
2. If yes, have you smoked more than five cigarettes in total in the last 8 weeks?

If the participant answered 'No' to the first question, or 'Yes' to the first question and 'No' to the second question, they were contacted by a research nurse to arrange an appointment to obtain a CO reading. Participants who had a CO reading less than or equal to the accepted threshold for their site had to provide a saliva sample and once the saliva sample was collected/received, they were sent a gift voucher with a value of £200.

2.3.2 Changes to the intervention in response to the Covid-19 pandemic

Due to the Covid-19 pandemic, SSS were disrupted. As a result, on the 16th of March 2020 the following changes were made for the intervention group:

- Participants who received SSS behavioural support and set a quit date via telephone (where this would have previously been conducted face-to-face) were considered to have engaged with the SSS, and received a gift voucher with the value of £50.
- Participants who self-reported as having quit with no CO verification at the 4-week follow-up received a gift voucher with the value of £50.
- Participants who self-reported as having quit at the 4-week follow-up were contacted at the 12 week follow-up. If they self-reported as quit with no CO verification at the 12-week follow-up they were sent a gift voucher with the value of £100.
- Participants who self-reported as having quit at the primary outcome stage in late pregnancy and for whom a saliva sample was received by the trial team were sent a gift voucher with the value of £200.

2.4 Usual care

Stop smoking support is freely available to pregnant women throughout the UK. Models of support differ however depending on where women live. In general, two main types of support are offered which can be described as ‘specialist’ (just for pregnant women) or ‘generic’ (for all smokers including pregnant women). Within this framework, support offered commonly includes: (1) individual/group support provided by specially trained advisers who may be nurses, or midwives, (2) support provided in hospital setting, women’s homes or other mutually acceptable venue, (3) at least one face-to-face counselling session with follow-up support, often by telephone, to 12 weeks after a quit date is set, and (4) advice on use of NRT utilising various models of prescribing (e.g. nurse/GP prescribing/pharmacy).

The National Institute of Health and Care Excellence (NICE) - PH26 Smoking: stopping in pregnancy and after childbirth published comprehensive guidance in 2010 regarding services that should be provided to pregnant smokers (National Institute for Health and Care Excellence. Quitting smoking in pregnancy and following childbirth. 2010. https://www.nice.org.uk/guidance/ph26).

2.5 Outcomes

2.5.1 Primary Outcome

The primary outcome is cotinine/anabasine verified abstinence from smoking for at least 8 weeks towards the end of pregnancy. All participants were contacted by the Trial Team at a random date between 34 and 38 weeks gestation and asked the following questions:

1. Have you smoked at all in the last eight weeks?
2. If yes, have you smoked more than five cigarettes in total in the last 8 weeks?

If the participant answered ‘No’ to the first question, or ‘Yes’ to the first question and ‘No’ to the second question, they were contacted by a research nurse to arrange an appointment to biochemically verify their smoking status. Participants were asked to provide a CO reading and a saliva sample (or urine sample when saliva collection could not be tolerated), which was tested for cotinine in the first instance. Where the cotinine result was less than or equal to the threshold (Table 2) and the participant had not reported any NRT/e-cigarette use then the participant was defined as a biochemically verified non-smoker.

Participants who indicated current NRT/e-cigarette use and had a saliva cotinine result < 10ng/ml were defined as biochemically verified non-smokers. Where participants indicated current NRT/e-cigarette use and the saliva cotinine result was ≥ 10ng/ml, saliva samples were also tested for anabasine. Where the anabasine result was less than or equal to the threshold 0.2ng/ml and the saliva cotinine result was ≥ 10ng/ml then the participant was defined as a biochemically verified non-smoker.

Urine samples were also collected from participants who indicated NRT/e-cigarette to allow further assaying in the event of any dubiety. At present however there is no defined threshold for anabasine in urine and a judgement regarding smoking status would need to be taken by the Research team in conjunction with advice from ABS Labs in this scenario.

Table 2: Thresholds for cotinine samples in saliva

**Cotinine threshold, ng/ml**	
Saliva	10.0
Urine	50.0
Plasma	10.0

2.5.2 Secondary Outcomes

2.5.2.1 Engagement with SSS (Locally Defined) and Setting of Quit Date Before 26 weeks Gestation

A participant was defined to have engaged with SSS if they had attended an appointment with a smoking cessation advisor (face-to-face or by telephone) and agreed a quit date before reaching 26 weeks gestation (calculated using the estimated delivery date and the antenatal booking appointment date).

2.5.2.2 CO-validated abstinence from smoking for at least 14 days at four weeks after quit date

At the four-week stage, participants were asked the following question:

- Have you smoked (even a puff) in the last two weeks?

If the participant answered ‘Yes’ to this question, they were defined as a self-reported smoker at the four week time point, and if the participant answered ‘No’ they were defined as a self-reported non-smoker. Participants who provided a CO result less than or equal to the CO threshold for their site were defined as CO-validated non-smokers. Participants whose four-week follow-up was due after the 16th of March 2020 were not able to provide CO samples due to restrictions implemented in response to the Covid-19 pandemic.

2.5.2.3 Cotinine/anabasine verified self-reported point abstinence from smoking for at least 8 weeks at 6 months post-partum

At the 6 months post EDD stage, participants were asked the following question:

- Have you smoked at all in the past eight weeks?

If the participant answered ‘No’ to this question, they were defined as a self-reported non-smoker at the six months post EDD stage. If the participant answered ‘Yes’, they were asked a second question:

- Have you smoked more than five cigarettes in total in the last eight weeks?

If the participant answered ‘No’ to this question, they were defined as a self-reported non-smoker. If the participant answered ‘Yes’, they were defined as a self-reported smoker.

If a participant was defined as a self-reported non-smoker, they were asked to provide a CO reading, and a saliva/urine sample in order for their smoking status to be biochemically verified. The sample was tested for cotinine and a participant defined as a biochemically verified non-smoker where the result was less than or equal to the threshold (Table 2) and the participant had not reported any NRT/e-cigarette use.

Participants who indicated current NRT/e-cigarette use and had a saliva cotinine result < 10ng/ml were defined as biochemically verified non-smokers. Where participants indicated current NRT/e-cigarette use and the saliva cotinine result was ≥ 10ng/ml, saliva samples were also tested for anabasine. Where the anabasine result was less than or equal to the threshold 0.2ng/ml and the saliva cotinine result was ≥ 10ng/ml then the participant was defined as a biochemically verified non-smoker.

2.5.2.4 Cotinine/anabasine verified self-reported continuous abstinence from smoking from late pregnancy to 6 months post-partum

At the 6 months post EDD stage, participants were asked the following question:

- Have you smoked since your baby was born?

If the participant answered 'No' to this question, they were defined as a self-reported continuous non-smoker at the six months post EDD stage. If the participant answered 'Yes', they were asked a second question:

- Have you smoked more than five cigarettes in total since your baby was born?

If the participant answered 'No' to this question, they were defined as a self-reported continuous non-smoker. If the participant answered 'Yes' they were defined as a self-reported continuous smoker.

If a participant was defined as a self-reported non-smoker, they were asked to provide a CO reading, and a saliva/urine sample in order for their smoking status to be biochemically verified. The sample was tested for cotinine and a participant defined as a biochemically verified non-smoker where the result was less than or equal to the threshold (Table 2) and the participant had not reported any NRT/e-cigarette use.

Where participants indicated current NRT/e-cigarette use and the saliva cotinine result was >= 10ng/ml saliva samples were also tested for anabasine. Where the anabasine result was less than or equal to the threshold 0.2ng/ml then the participant was defined as a biochemically verified non-smoker.

2.5.2.5 Birth Weight

The weight of the baby in kilograms was collected to two decimal places. If the participant gave birth to more than one baby, the weight of the lightest baby was used.

2.5.3 Adverse events

Serious adverse events (SAEs) that are related to the intervention will be documented. It is not anticipated that the provision of shopping vouchers to women will be associated with any related SAEs.

2.5.4 Other Collected Data

- *Demographics:* Maternal age, height and weight, household income and ethnicity were collected at baseline.
- *Index of Multiple Deprivation (IMD) quintile:* The IMD is a measure that ranks each lower layer super output area (LSOA) in order of deprivation, with the most deprived LSOA being ranked the highest. The IMD was derived for each participant by mapping to the participant's postcode. From the IMD the IMD quintile was then derived. A value of 1 represents the most deprived quintile, while 5 represents the least deprived. The postcode used to derive the IMD quintile was collected at baseline.
- *The Fagerström Test for Nicotine Dependence (FTND):* The FTND is used for assessing nicotine dependence. The test is composed of six questions, the scoring of which is detailed in Appendix 8.1. The FTND was collected at baseline.
- *Other smoking information:* The age at which the participant started smoking and whether the participant was currently living with someone who smokes were also collected at baseline, along with information on whether the participant used NRT and/or e-cigarettes.

- *Antenatal appointment information:* The date of the antenatal appointment and the CO reading taken as part of routine care (where available) were collected.
- *EQ-5D-5L:* The EQ-5D-5L was collected as a standardised measure of current health status developed by the EuroQol Group for clinical and economic appraisal. The EQ-5D-5L consists of five questions and a visual analogue scale, each assessing a different quality of life dimension (Mobility, Self-care, Usual activities, Pain/Discomfort and Anxiety/Depression). A weighted and population referenced summary index is derived and will be reported and analysed as part of the health economic analysis.
- *Other birth data:* Parity and the baby's birth date was collected as well as the status of baby at birth (live/stillbirth).
- *Neonatal stay:* For babies admitted to neonatal care, length of stay was recorded.
- *Miscarriage data:* Any known occurrence of miscarriage was collected throughout the study duration. The date the event became known to the trial team and date of miscarriage (where available) was collected.

2.6 Sample size

The aim was to recruit 940 participants to the trial (470 per arm). This gave 90% power to detect a doubling of the smoking cessation rate from 7% to 14%, allowing 15% loss to follow-up. The smoking cessation rate in the control group was derived from the smoking cessation rate found in the feasibility trial [2], and two other large trials of smoking cessation interventions in pregnant smokers [3, 4]. The smoking cessation rate in the intervention group was derived from the cessation rate in the feasibility trial, along with considerations of the effect size that would be considered clinically important.

2.7 Randomisation

Participants were allocated using a 1:1 allocation ratio to either intervention or control using random permuted blocks with randomly varying block sizes. No stratification factors were used when randomising the participants.

To try to prevent participants in the incentives arm using the timing of the primary outcome to 'game' the study and falsely obtain the final incentives voucher, all participants were randomly assigned a date for primary outcome follow-up between 34 and 38 weeks gestation, which was calculated using the participant's EDD.

2.8 Blinding

Due to the nature of the intervention, it was not possible to blind participants to treatment allocation in this pragmatic trial. In addition, due to the design of the trial, it was not possible for the statistician to be blinded to the treatment allocation. However, collection of self-reported smoking status at 34-38 weeks gestation was initiated blind to treatment allocation.

2.9 Follow-up

Follow-up of participants was undertaken at the engagement stage, 4 week post quit date, 12 week post quit date, 34-38 weeks gestation and six months post-partum (see section 2.9.5.1 for details of changes of the timing of the six months postpartum follow-up). A brief graphical outline of follow-up is given in Figure 1.

2.9.1 Follow-up 1: SSS Engagement

After the participant consented and was informed of group allocation, trial research staff contacted the participant's local SSS to ascertain if the participant attended a first appointment with an SSS advisor and set a quit date. This information was entered into the trial database for both control and intervention group participants. A £50 voucher was automatically dispatched to intervention group participants who attended and set a quit date.

2.9.2 Follow-up 2: Four weeks post quit date

For participants who engaged with the SSS and set a quit date, trial research staff contacted the participant's local SSS four weeks after this quit date to obtain smoking status in the last two weeks and CO breath test result as recorded by the SSS. Where a breath test result was not available from the SSS, trial research nurses collected this directly from the woman in the incentives group to initiate incentive payments. CO breath test results were collected for the control group only where these were available from the SSS in line with national SSS guidelines. This information was entered onto the trial database. If the CO result was at or below the accepted level for a non-smoker at the site, a £50 voucher was automatically dispatched to women in the incentives group.

2.9.3 Follow-up 3: 12 weeks post quit date

For participants in the intervention group who were confirmed quit at four weeks, trial research staff contacted the participant's local SSS eight weeks later to obtain smoking status and CO breath test result as recorded by the SSS. Where this was not available from the SSS, trial research nurses collected this directly from the participant. This information was entered into the trial database. If the CO result was at or below the accepted level for a non-smoker at the site, a £100 voucher was automatically dispatched.

2.9.4 Follow-up 4: Late pregnancy (34-38 weeks gestation)

All participants were followed up at the primary outcome stage in late pregnancy. Follow-up telephone contact was attempted by the trial contact centre at a random date between 34 and 38 weeks gestation allocated at the time of initial randomisation. Trial research nurses reviewed participants' notes one week prior to the telephone contact to check the health status of mother and baby and alert TCC staff to any adverse events e.g. miscarriage or stillbirth, that required particular sensitivity when conducting follow-up. TCC staff were blind to group allocation.

Three attempts were made by the TCC to contact women. If no contact was established, local research staff followed up women by telephone, text, and letter. On successful contact, women were asked: 'Have you smoked in the last 8 weeks?' If yes, 'Have you smoked more than 5 cigarettes in that time?'. EQ-5D-5L data, and current NRT/electronic cigarette use were also collected at this time point.

Self-report of not smoking was corroborated by cotinine estimation on saliva or urine (when saliva collection could not be tolerated). Where women were using NRT or e-cigarettes, anabasine assay on saliva was also conducted. Cotinine and anabasine were assayed by ABS Laboratories Limited (https://www.acmgloballab.com/about-us/our-locations/europe-london-uk). To minimise the potential for women to 'game' the primary outcome, incentive payments were dependent on the CO result, which is an immediate measure, and not on the cotinine or anabasine level.

An important aspect of the primary outcome for this phase III trial is the proportion of women successfully followed up in both the intervention and control group. To minimise loss to follow-up, particularly among controls, women in both groups will receive Love2Shop vouchers of £50 and £25 for providing data and saliva/urine samples where applicable at the primary (late pregnancy) and secondary (six months post-partum) outcome time points respectively (Figure 2.).

To assess if a) women lost to trial follow-up are still smoking towards the end of pregnancy, and b) the primary outcome has been 'gamed' (saliva cotinine below the cut-off but still smoking in late pregnancy) residual blood from routine late pregnancy samples, where available, will be tested.

2.9.5 Follow-up 5: Six months postpartum

Similar to the late pregnancy follow-up all women were contacted at six months after their expected delivery date to ascertain smoking status and collect a saliva/urine sample for those women who self-reported as quit. Quit status six months after birth was ascertained by two sets of questions:
1. 'Have you smoked in the last 8 weeks?' If yes 'Have you smoked more than 5 cigarettes in that time?', and 2. 'Have you smoked since your baby was born?' If yes, 'Have you smoked more than 5 cigarettes in total since your baby was born?'

Follow-up procedures (i.e. no. of contact attempts, data collection and saliva/urine sample collection and assay) were the same as those described for the late pregnancy follow-up. Biological samples of saliva and urine will not be available for use by other researchers.

2.9.5.1 Change to six months postpartum follow-up

Due to funding restrictions, data collection will end on 31/10/2020, and as a result it will not be possible to follow-up all participants to six months postpartum. Approximately 65% of women were followed up at six months postpartum as planned. The remaining 35% were followed up at less than six months postpartum by the TCC, as the follow-up period of the study was not long enough to follow-up these women at six months postpartum. Women who were followed up earlier than six month postpartum were contacted by the TCC between 01/08/2020 and 30/09/2020. Participants who could not be contacted by the TCC during this time period will be followed-up by research nurses up to 31/10/2020.

Table 3: CPIT III Data Collection Schedule

	**Timepoint obtained/measured**						
	**Baseline**	**Follow-up 1: SSS Engagement**	**Follow-up 2: 4 weeks post quit date**	**Follow-up 3: 12 weeks post quit date**	**Follow-up 4: Late pregnancy (34-38 weeks gestation)**	**Delivery**	**Follow-up 5: 6 months post-partum**
Age	x						
Height	x						
Weight	x						
Ethnicity	x						
English speaking	x						
Household income	x						
Deprivation quintile	x						
Quality of Life	x				x		x
Event dates	x	x	x	x	x	x	x
Age first smoked	x						
Nicotine dependence level	x						
SSS attendance		x	x	x			
Self-reported smoking status	x		x	x	x		x

	Timepoint obtained/measured						
	**Baseline**	**Follow-up 1: SSS Engagement**	**Follow-up 2: 4 weeks post quit date**	**Follow-up 3: 12 weeks post quit date**	**Follow-up 4: Late pregnancy (34-38 weeks gestation)**	**Delivery**	**Follow-up 5: 6 months post-partum**
CO breath test result	x	x	x	x	x		x
Quit date		x					
Urine/saliva cotinine/ anabasine					x		x
Current E-cig use	x				x		x
Current NRT use	x	x			x		x
Expected date of delivery	x						
Actual date of delivery						x	
Multiple birth						x	
Birthweight						x	
Stillbirth/ Miscarriage		x	x	x	x	x	
Parity						x	

3 Study Data

3.1 Trial Data

Data extracts without direct identifiers are passed to York Trials Unit from the Database Management Company using sFTP encryption in transit.

3.2 External datasets

Screening logs were received from each site on a monthly basis via the University of York DropOff Service.

Data was downloaded from the following websites to calculate the index of multiple deprivation for postcodes in England, Wales, Scotland and Northern Ireland:

- https://www.gov.uk/government/statistics/english-indices-of-deprivation-2015 (accessed 08/10/2018, published September 2015)
- https://statswales.gov.wales/Catalogue/Community-Safety-and-Social-Inclusion/Welsh-Index-of-Multiple-Deprivation/WIMD-2014/wimd2014 (accessed 08/10/2018, published November 2014)
- https://www.gov.scot/Topics/Statistics/SIMD (accessed 08/10/2018, published August 2016)
- https://ons.maps.arcgis.com/home/item.html?id=ef72efd6adf64b11a2228f7b3e95deea (accessed 08/10/2018, published August 2016)

- https://www.nisra.gov.uk/publications/nimdm17-sa-level-results (accessed 27/08/2018, published November 2017)

The postcodes for Northern Ireland were downloaded from the Central Postcode Directory on 27/08/2018, and were published in November 2015. Data from the Central Postcode Directory is not freely available to the public.

3.3 Management of Datasets and Data Verification

The Database Management Company has a long history of managing government related services and is able to demonstrate commitment to data security and quality management through ISO27001 and ISO9001 accreditations and recent GDPR legislation. ISO27001 accredited Information Security Management Systems demand that all of systems and processes are maintained with confidentiality, integrity and availability of data at the core. In addition, the Database Management Company is ISO9001 accredited, the internationally recognized standard for Quality Management Systems. Regular external audits ensure adherence to ISO9001 and ISO27001 standards.

The Business Requirements Specification documents stored in the Y Drive contain comprehensive details of both functional and non-functional requirements of the study database. These documents incorporate all data validation/verification rules applied.

3.4 Location of Data and Associated Files

Data and documents relevant to the statistical analysis will be kept electronically (Y:\ Project -- A - Statistics).

4 Analysis

4.1 Strategy for reporting data and general considerations

Data will be analysed and reported according to CONSORT guidelines [5]. A CONSORT diagram will be produced (see Figure 2). Analyses will be conducted using Stata version 16 or later [6]. The version of Stata to be used will be confirmed in the final report. All analyses will be conducted following the principle of intention-to-treat unless stated otherwise. Statistical tests will be two-sided at the 5% significance level. Effect size estimates will be presented with 95% confidence intervals.

4.2 Recruitment and attrition

The number of participants screened, consented and randomised to the trial will be reported, along with reasons for ineligibility. All withdrawals will be reported along with the reasons, where given, for withdrawal.

4.3 Baseline data

All baseline data will be summarised descriptively by trial arm. In addition, baseline data by trial arm will be presented for those who provided a smoking status at the primary outcome follow-up (Table 4) [7]. No formal statistical comparisons will be undertaken [8]. Continuous measures will be reported as means and standard deviations (and/or median, interquartile range, and minimum and maximum as appropriate) while categorical data will be reported as counts and percentages.

4.4 Primary outcome analysis

The primary outcome will be analysed using a mixed-effects logistic regression model adjusting for treatment group, age, years of smoking, income status (as measured by the IMD quintile within the participant’s nation), level of smoking (as measured by the Fagerström score) and whether the primary outcome was collected before the 16th of March

2020 (the date on which the intervention changed due to the Covid-19 pandemic), with centre as a random effect.

For a small number of participants in Site C, a biochemical sample was provided but due to a miscommunication with the lab the sample was not analysed for anabasine. It was decided with the TSC that in this scenario, those who self-reported as quit and had a CO reading less less than 4 ppm would be classed as non-smokers for the primary analysis.

If a participant is missing data on the primary outcome, it shall be assumed the participant is smoking, as per the Russell Standard [9]. If a participant is missing data on any covariates to be included in the analysis models, the missing values will be replaced with centre-specific means [10]. The assumptions of the model will be checked. If the assumptions are found to be questionable, the data will be transformed or non-parametric data analysis methods will be used.

It was considered whether sparse data bias would be an issue when conducting the primary analysis [11]. Given the sample size calculation assumption that 7% of participants in the control group would quit, it can be assumed that if the full sample size of 940 are recruited and the intervention has no effect, then the ‘event’ of a participant quitting smoking at the late pregnancy stage would be expected to occur approximately 66 times during the study. The primary analysis model contains five fixed effect variables, which means approximately 13 events per variable are expected. This exceeds the recommended minimum of 10 events per variable when using logistic regression and as a result it was not considered necessary to use an alternative statistical model [12].

4.5 Secondary outcome analyses

Engagement with SSS will be analysed using a mixed-effects logistic regression model adjusting for treatment group, age, years of smoking, income status, level of smoking (as measured by the Fagerström score) and whether the engagement data was collected before the 16th of March 2020, with centre as a random effect.

CO-validated smoking status at 4 weeks post-quit date will be analysed using a mixed-effects logistic regression model, adjusting for treatment group, age, years of smoking, income status, level of smoking (as measured by the Fagerström score) and whether the CO-validated smoking status was collected before the 16th of March 2020, with centre as a random effect. If a participant is missing their smoking status, it shall be assumed the participant is smoking.

Birth weight will be analysed using a mixed-effects linear regression model, adjusting for treatment group, the age, height and weight of the mother at booking, years of smoking, income status, level of smoking (as measured by the Fagerström score) and whether the birthweight data was collected before the 16th of March 2020 as fixed effects, and centre as a random effect. If a participant is missing data on birth weight, it shall be assumed the data is missing at random conditional on the covariates included in the analysis model [13, 14].

Continuous and point abstinence outcomes obtained at six months postpartum will be analysed using mixed-effects logistic regression adjusting for the same covariates and applying the same assumptions used in the primary outcome analysis (with the exception that whether the postpartum point abstinence outcome was collected before 16th of March 2020 will be adjusted for instead of adjusting for whether the primary outcome was collected before the 16th of March 2020). If a participant is missing their smoking status, it shall be assumed the participant is smoking. Participants who at postpartum were followed up earlier than 6 months will be excluded from the analysis of continuous and point abstinence

outcomes obtained at six months postpartum, and will be accounted for in a sensitivity analysis.

4.6 Exploratory outcome analysis

Length of neonatal stay will be summarised descriptively by treatment group.

4.7 Sensitivity analyses

4.7.1 Sensitivity of primary analysis to sparse data bias

To assess the sensitivity of the primary analysis to sparse data bias, the primary outcome will be analysed using a Firth logistic regression model adjusting for treatment group, age, years of smoking, income status (as measured by IMD score), level of smoking (as measured by the Fagerström score), whether the primary outcome was collected before the 16th of March 2020 and site [11]. All variables will be adjusted for as fixed effects, as the Stata command used to implement Firth logistic regression cannot incorporate random effects.

4.7.2 Impact of the Covid-19 pandemic on analysis of the primary and secondary smoking outcomes

To assess the impact of the Covid-19 pandemic on the primary and secondary smoking outcomes, the number of self-reported non-smokers pre-Covid and post-Covid will be compared descriptively by treatment group (Table 6). This will allow assessment of the possibility that participants in the incentives group were more likely to report as non-smokers to receive financial incentives post-Covid compared to pre-Covid. In addition, the number of participants who self-reported as non-smokers but provided a positive biochemical sample pre-Covid and post-Covid will be compared descriptively by treatment group (Table 7).

The number of biochemically verified non-smokers pre-Covid and post-Covid will also be compared descriptively by treatment group (Table 8), in order to assess the possibility that the treatment effect pre-Covid is different to the treatment effect post-Covid.

If the above checks indicate the Covid-19 pandemic had an impact on the analysis of the primary and secondary smoking outcomes, the analyses of these outcomes shall be repeated with the addition of an interaction term between treatment allocation and a pre-Covid/post-Covid variable, with effect size estimates pre-Covid and post-Covid presented alongside corresponding 95% confidence intervals and p-values.

Finally, in order to assess whether the change in intervention had an impact on the return of biochemical samples, the number of returned biochemical samples pre-Covid and post-Covid will be compared descriptively by treatment group in the subset of self-reported non-smokers (Table 9).

4.7.3 Subgroup analyses

The primary analysis will be repeated, with the addition of interaction terms between treatment group and each of the following covariates:

- Maternal age (≤28 years vs >28 years, with 28 years being the mean age of a first time mother according to the ONS in 2015)
- Index of multiple deprivation quintile (1/2/3/4/5)
- Years of smoking (≤10 years vs >10 years)
- Fagerström score (the cut-off will be decided using data on the distribution of the Fagerström score in the previous CPIT RCT)

4.7.4 Missing data analyses

If a participant is missing data on the primary outcome, the participant shall be assumed to be smoking, as per the Russell Standard [9]. The robustness of the primary analysis to this assumption will be explored using two methods.

The first method will use multiple imputation by chained equations [15]. Missing values of baseline covariates will be replaced with centre-specific means. The imputation model for the biochemically verified smoking status at late pregnancy will include the baseline covariates used in the primary analysis, the treatment allocation, engagement with SSS, CO-validated smoking status at 4 weeks post-quit and biochemically verified point abstinence at 6 months post-partum.

Each imputed dataset will be analysed using the primary analysis model. The estimates obtained from analysis of the imputed datasets will then be combined using Rubin's rules.

The second method will explore the sensitivity of the results to the missing data mechanism using a pattern mixture model, which will be implemented using the `rctmiss` command in Stata [16]. The pattern mixture model expresses assumptions about the missing data mechanism in the form of a logistic regression model regressing the outcome on a set of covariates and a missing data indicator, whose parameter shall be denoted as β_m. The Russell standard is equivalent to assuming that $\beta_m = -\infty$ i.e. missing=non-quitter. Negative values of β_m assume that participants with missing smoking status are less likely to have quit than participants with non-missing smoking status, while positive values assume that participants with missing smoking status are more likely to have quit than participants with non-missing smoking status. The consequences of varying β_m over a range of values shall be explored and displayed graphically.

4.7.5 Impact of participants who were followed up at postpartum earlier than the planned 6 months

The number and proportion of participants who were followed up earlier than 6 months postpartum will be summarised descriptively by trial arm. The time in months between the planned follow-up date and actual follow-up date will be summarised descriptively by trial arm.

Participants who provide a smoking status postpartum will be grouped by whether the postpartum follow-up took place between:

- Less than two months postpartum
- More than or equal to two months postpartum and less than four months postpartum
- More than or equal to four months postpartum and less than six months postpartum
- More than or equal to six months postpartum

Within these groups, the number and proportion of participants who were cotinine/anabasine validated non-smokers will be summarised descriptively.

4.7.6 Sensitivity of primary analysis to assumption regarding participants with missing anabasine test

In the primary analysis it will be assumed that for those participants who provided a biochemical sample but due to a miscommunication with the lab were not tested for anabasine, that if they have a CO reading less than 4 ppm then they are non-smokers. The impact of this assumption on the primary analysis will be explored by repeating the primary analysis under two different scenarios. The first scenario will assume that the participants in the incentives group were smokers while the participants in the control group were non-

smokers. The second scenario will assume the participants in the incentives group were non-smokers while the participants in the control group were smokers.

4.8 Compliance with intervention

A CACE analysis for the outcome of birth weight will be used to obtain an unbiased estimate of the effect of the intervention with full compliance (defined as the participant being found to be a biochemically verified non-smoker at late pregnancy). An instrumental variable model will be used, using the compliance variable as the endogenous variable, and treatment group, the age, height and weight of the mother at booking, years of smoking, income status, level of smoking (as measured by the Fagerström score), whether the engagement data was collected before the 16th of March 2020, and centre as exogenous variables.

4.9 Gaming of the intervention

To assess the extent to which participants 'gamed' the primary outcome i.e. stopped smoking a few days before sample collection, the number and proportion of women who were found to be biochemically verified non-smokers but tested positive for smoking in late pregnancy residual blood samples (where available) will be summarised descriptively by treatment group.

4.10 Cotinine and anabasine test results

For participants who reported as non-smokers and using e/cigarettes or NRT at late pregnancy, the results of the cotinine and anabasine tests will be summarised descriptively, in order to assess the impact of anabasine testing on the derivation of the primary outcome (Table 10). The analysis will be repeated for the 6 months post-partum time point.

4.11 Adverse events

Adverse events related to the study will be presented descriptively by treatment group.

4.12 Planned interim review and analyses

No formal interim analyses will be undertaken.

5 SAP amendment log

Amendment/addition to SAP and reason for change	New version number, name and date
The derivation of biochemically verified smoking status was changed in line with the latest cut-offs advised by ABS laboratories (Table 2).<br><br>A descriptive analysis of cotinine and anabasine results was added (Section 4.10 and Table 10).<br><br>Amendment of analysis of smoking status at 4 weeks, birth weight and sparse data sensitivity analysis to include additional covariates (Sections 4.5, 4.7.1 and 4.8). For all three analyses, whether the outcome was collected before the 16th March 2020 was included as an additional covariate. For the analysis of birth weight, years of smoking, income status and level of smoking were also included as additional covariates.	CPIT III Statistical Analysis Plan_1.1_20210728

6 Signatures of approval

6.1 Contributions

AM and AK drafted the Statistical Analysis Plan; however, sections of this document have been copied and/or adapted from the trial protocol. This document will be reviewed by members of the TMG and TSC.

6.2 Signatures

Sign-off of the Statistical Analysis Plan by, as a minimum, the person writing the SAP, a relevant senior statistician, and the Chief Investigator.

Name	Trial Role	Signature	Date
Prof. David Tappin	Chief Investigator	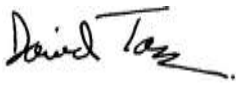	29/07/2021
Prof. Linda Bauld	Chief Investigator	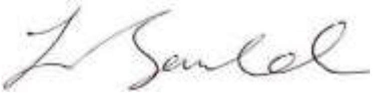	05/08/2021
Alex Mitchell	Study Statistician	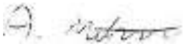	28/07/2021
Prof. Catherine Hewitt	Senior Statistician	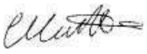	28/07/2021

7 References

1. Sinclair, L., et al., *The smoking cessation in pregnancy incentives trial (CPIT): study protocol for a phase III randomised controlled trial.* Trials, 2020. **21**(1): p. 1-12.
2. Tappin, D., et al., *Financial incentives for smoking cessation in pregnancy: randomised controlled trial.* Bmj, 2015. **350**: p. h134.
3. Coleman, T., et al., *A randomized trial of nicotine-replacement therapy patches in pregnancy.* New England Journal of Medicine, 2012. **366**(9): p. 808-818.
4. Ussher, M., et al., *Physical activity for smoking cessation in pregnancy: randomised controlled trial.* bmj, 2015. **350**: p. h2145.
5. Moher, D., et al., *CONSORT 2010 explanation and elaboration: updated guidelines for reporting parallel group randomised trials. BMJ 340: c869.* doi. org/10.1136/bmj. c869, 2010.
6. StataCorp, *Stata Statistical Software: Release 15*. 2017: College Station, TX: StataCorp LLC.
7. Dumville, J.C., D.J. Torgerson, and C.E. Hewitt, *Reporting attrition in randomised controlled trials.* Bmj, 2006. **332**(7547): p. 969-971.
8. Senn, S., *Testing for baseline balance in clinical trials.* Statistics in medicine, 1994. **13**(17): p. 1715-1726.
9. West, R., et al., *Outcome criteria in smoking cessation trials: proposal for a common standard.* Addiction, 2005. **100**(3): p. 299-303.

10. White, I.R. and S.G. Thompson, *Adjusting for partially missing baseline measurements in randomized trials.* Statistics in medicine, 2005. **24**(7): p. 993-1007.
11. Greenland, S., M.A. Mansournia, and D.G. Altman, *Sparse data bias: a problem hiding in plain sight.* bmj, 2016. **352**: p. i1981.
12. Peduzzi, P., et al., *A simulation study of the number of events per variable in logistic regression analysis.* Journal of clinical epidemiology, 1996. **49**(12): p. 1373-1379.
13. Kang, H., *The prevention and handling of the missing data.* Korean journal of anesthesiology, 2013. **64**(5): p. 402.
14. White, I.R., et al., *Strategy for intention to treat analysis in randomised trials with missing outcome data.* Bmj, 2011. **342**: p. d40.
15. White, I.R., P. Royston, and A.M. Wood, *Multiple imputation using chained equations: issues and guidance for practice.* Statistics in medicine, 2011. **30**(4): p. 377-399.
16. White, I., *RCTMISS: Stata module to analyse a randomised controlled trial (RCT) allowing for informatively missing outcome data.* 2018.

8 Appendices

8.1 Scoring of Fagerström Test for Nicotine Dependence

The Fagerström Test for Nicotine Dependence is scored in the following manner:

- How soon after waking do you smoke your first cigarette?
    - (3) Within 5 minutes
    - (2) 5-30 minutes
    - (1) 31-60 minutes
- Do you find it difficult to refrain from smoking in places where it is forbidden?
    - (1) Yes
    - (0) No
- Which cigarette would you hate to give up?
    - (1) The first in the morning
    - (0) Any other
- How many cigarettes a day do you smoke?
    - (3) 31 or more
    - (2) 21-30
    - (1) 11-20
    - (0) 10 or less
- Do you smoke more frequently in the morning?
    - (1) Yes
    - (0) No
- Do you smoke even if you are sick in bed most of the day?
    - (1) Yes
    - (0) No

The Fagerström score is calculated by adding up the scores from each question, and can take values between 0 and 10. There is no published advice in regards to how to account for missing responses to the Fagerström questionnaire.

8.2 Trial flow diagram

Figure 1: A trial flow diagram detailing the flow of participants through the study and the incentives on offer to the intervention group. The shaded boxes indicate the trial team is not involved in this follow-up stage.

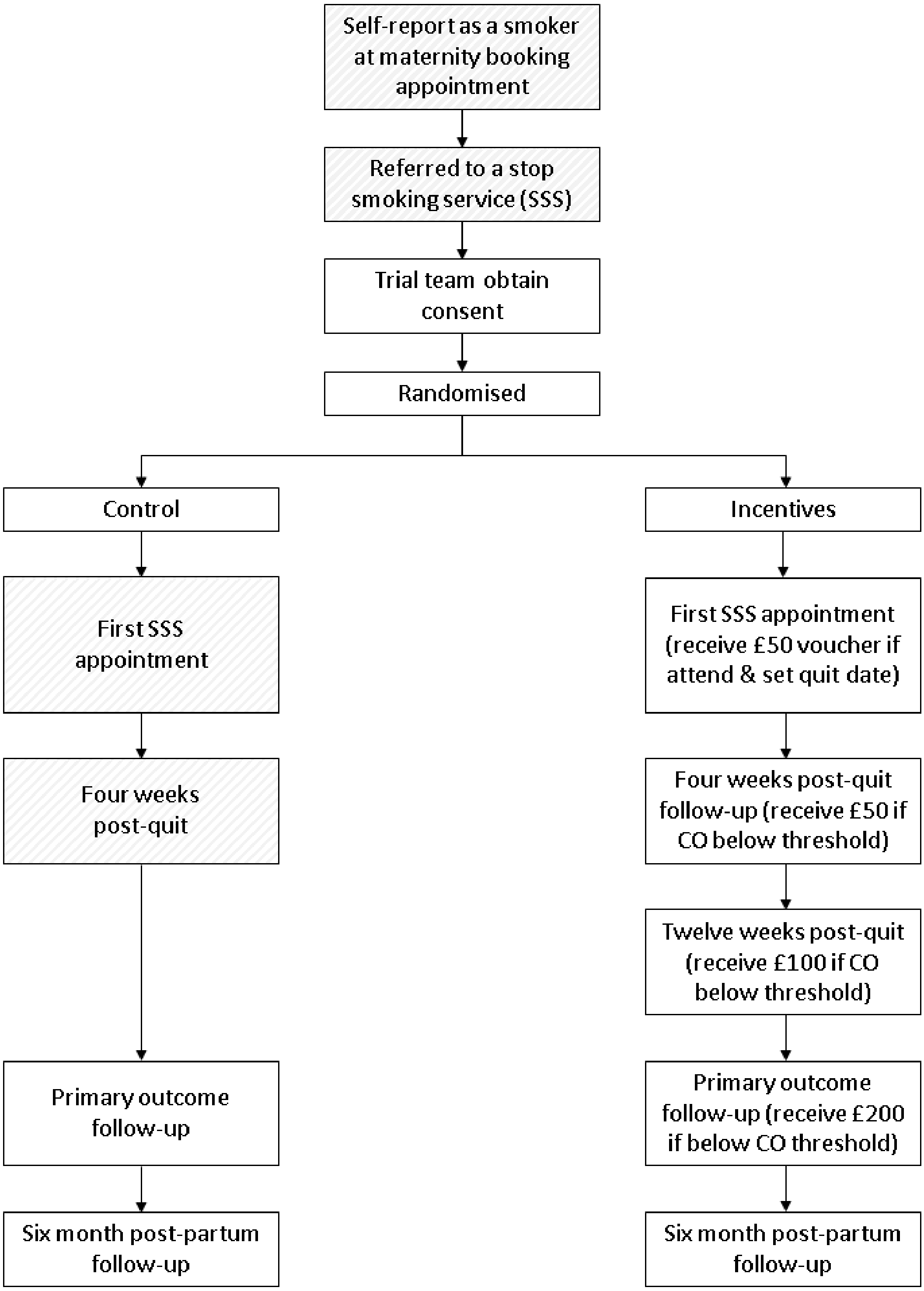

8.3 CONSORT diagram

Figure 2: A CONSORT diagram for CPIT III.

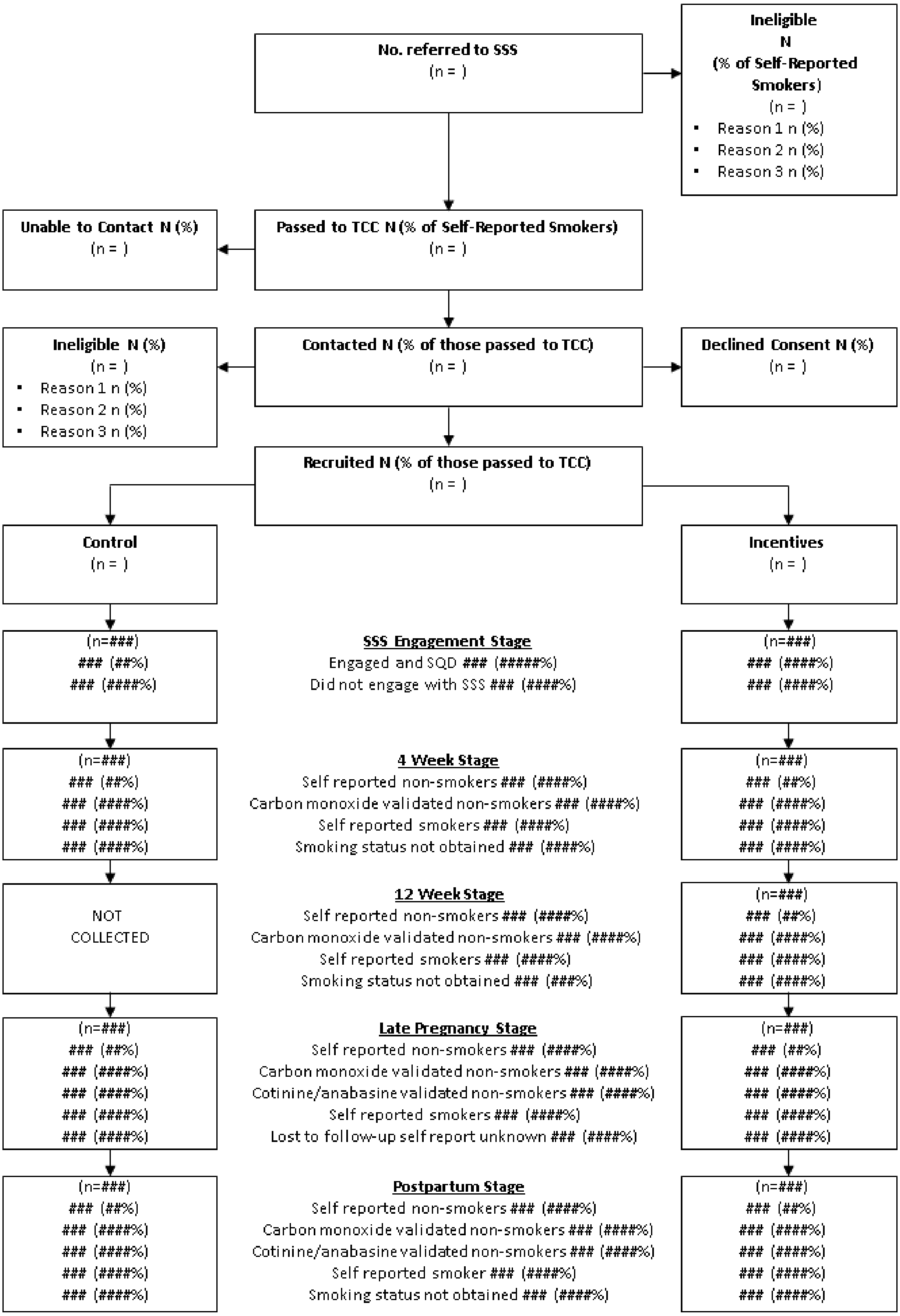

8.4 Example tables

8.4.1 Baseline characteristics

Table 4: Baseline characteristics for all participants and for participants who provided a cotinine/anabasine verified smoking status at the primary outcome follow-up.

	All randomised participants		Participants who provided a smoking status at the primary outcome stage	
	**Control (n=)**	**Intervention (n=)**	**Control (n=)**	**Intervention (n=)**
**Height, m**<br>n (%)<br>Mean (SD)				
**Weight, kg**<br>n (%)<br>Mean (SD)				
**BMI, kg/m^2**<br>n (%)<br>Mean (SD)				
**Ethnicity, n (%)**				
**Maternal age at booking, years**<br>n (%)<br>Mean (SD)				
**Previous live births**<br>Median (range)				
**Index of multiple deprivation, n (%)**<br>1st quintile (most deprived)<br>2nd quintile<br>3rd quintile<br>4th quintile<br>5th quintile (least deprived)				
**CO reading at maternity booking, ppm**<br>n (%)<br>Mean (SD)				
**First cigarette within 5 minutes of waking, n (%)**<br>Within 5 minutes<br>5-30 minutes<br>31-60 minutes				
**Difficulty not smoking in forbidden places, n (%)**<br>Yes<br>No<br>Missing				
**1st cigarette most difficult to give up, n (%)**<br>The first in the morning<br>Any other<br>Missing				
**Cigarettes smoked a day, n (%)**<br>10 or less<br>11-20<br>21-30<br>31 or more<br>Missing				
**Smoke more frequently in the morning, n (%)**				

Yes<br>No<br>Missing				
**Smoke even if sick in bed most of the day, n (%)**<br>Yes<br>No<br>Missing				
**Fagerström score**<br>n (%)<br>Mean (SD)				
**Partner smokes, n (%)**<br>Yes<br>No<br>Missing				
**Age at which participant started smoking, years**<br>n (%)<br>Mean (SD)				
**Uses NRT n (%)**<br>Yes<br>No<br>Missing				
**Uses e-cigarettes n (%)**<br>Yes<br>No<br>Missing				

Table 5: Primary and secondary analyses

	**Number of events/Number in group**	**Adjusted odds ratio (95% CI)**	**p-value**
**Late pregnancy**<br>Incentives<br>Usual care			
**Engagement with SSS**<br>Incentives<br>Usual care			
**4 week post-quit**<br>Incentives<br>Usual care			
**6 months post-partum (continuous abstinence)**<br>Incentives<br>Usual care			
**6 months post-partum (point abstinence)**<br>Incentives<br>Usual care			

Table 6: Comparison by treatment group of self-reported smoking status pre-Covid and post-Covid

	**Incentives**		**Usual care**	
	**Pre-Covid**	**Post-Covid**	**Pre-Covid**	**Post-Covid**
**4 week, n (%)**				

	Incentives		Usual care	
	Pre-Covid	Post-Covid	Pre-Covid	Post-Covid
Non-smoker<br>Smoker				
**12 week, n (%)**<br>Non-smoker<br>Smoker				
**Late pregnancy, n (%)**<br>Non-smoker<br>Smoker				
**6 months post-partum, n (%)**<br>Non-smoker<br>Smoker				

Table 7: Comparison by treatment group of the number of participants who provided a positive biochemical sample, in the subset of participants who self-reported as non-smokers and provided a biochemical sample

	Incentives		Usual care	
	Pre-Covid	Post-Covid	Pre-Covid	Post-Covid
**Self-reported as non-smoker and tested positive in biochemical sample, n (%)**<br>Late pregnancy<br>6 months post-partum				

Table 8: Comparison by treatment group of biochemically verified smoking status pre-Covid and post-Covid

	Incentives		Usual care	
	Pre-Covid	Post-Covid	Pre-Covid	Post-Covid
**4 week, n (%)**<br>Non-smoker<br>Smoker				
**12 week, n (%)**<br>Non-smoker<br>Smoker				
**Late pregnancy, n (%)**<br>Non-smoker<br>Smoker				
**6 months post-partum, n (%)**<br>Non-smoker<br>Smoker				

Table 9: Comparison by treatment group of the number of participants who provided a biochemical sample, in the subset of participants who self-reported as non-smokers

	Incentives		Usual care	
	Pre-Covid	Post-Covid	Pre-Covid	Post-Covid
**Provided a biochemical sample, n (%)**<br>Late pregnancy<br>6 months post-partum				

Table 10: Anabasine results presented by treatment group and cotinine result for patients who reported as non-smokers and reported using e-cigarettes or NRT.

	**Cotinine**			
	**Incentives**		**Usual care**	
	**<10ng/ml**	**≥10ng/ml**	**<10ng/ml**	**≥10ng/ml**
**Anabasine result, n (%)**				
≤0.2				
>0.2				

Appendix D. Changes to trial conduct methods forced by COVID with resulting outcomes before and during the COVID pandemic.

COVID stopped all carbon monoxide breath testing by Smoking Cessation Services and also home visits by Research Nurses. After 14/03/2020 the 4-week and 12-week vouchers were sent to intervention participants if they self-reported as abstinent when contacted by research staff. In late pregnancy and 6 months after birth self-reported abstinent participants were sent a saliva collection kit. Research staff telephoned abstinent participants and talked them through collection of the saliva sample. The final £200 voucher was triggered when a saliva sample for cotinine/anabasine estimation had been received at a central storage facility by research staff.

Outcomes

Table 4a presents a comparison by treatment group of self-reported smoking status pre-Covid and post-Covid (excluding three participants who withdrew and requested data be removed).

Table 4a

	**Incentives**		**Usual care**	
	**Pre-Covid**	**Post-Covid**	**Pre-Covid**	**Post-Covid**
**4 week, n (%)**				
Non-smoker	165 (37.2)	11 (39.3)	86 (19.4)	6 (22.2)
Smoker	109 (24.6)	7 (25.0)	112 (25.3)	8 (29.6)
Missing	169 (38.1)	10 (35.7)	245 (55.3)	13 (48.1)
**12 week[1], n (%)**				
Non-smoker	123 (79.4)	20 (76.9)	NA	NA
Smoker	18 (11.6)	5 (19.2)	NA	NA
Missing	14 (9.0)	1 (3.8)	NA	NA
**Late pregnancy, n (%)**				
Non-smoker	124 (34.6)	45 (39.8)	67 (18.8)	20 (17.7)

	Incentives		Usual care	
	Pre-Covid	Post-Covid	Pre-Covid	Post-Covid
Smoker	207 (57.8)	60 (53.1)	276 (77.3)	84 (74.3)
Missing	27 (7.5)	8 (7.1)	14 (3.9)	9 (8.0)
**6 months post-partum, n (%)**				
Non-smoker	17 (9.2)	28 (17.2)	12 (6.3)	26 (17.2)
Smoker	130 (70.3)	100 (61.3)	136 (71.2)	96 (63.6)
Missing	38 (20.5)	35 (21.5)	43 (22.5)	29 (19.2)

[1]Higher self-reported non-smoking rates at the 12 week time point likely to be higher than at other time points due only to only those who were confirmed quitters at 4 weeks being followed up at 12 weeks.

Table 5a presents a comparison by treatment group of the number of participants who provided a positive biochemical sample (or CO sample for the 4 and 12 week time points), in the subset of participants who self-reported as non-smokers and provided a biochemical/CO sample (excluding three participants who withdrew and requested data be removed).

	Incentives		Usual care	
	Pre-Covid	Post-Covid	Pre-Covid	Post-Covid
**Tested positive for smoking in CO sample at 4 weeks, n (%)**				
Positive	10 (6.2)	0 (0)	2 (3.4)	0 (0)
Negative	151 (93.8)	11 (100)	56 (96.6)	6 (100)
Missing	0 (0)	0 (0)	0 (0)	0 (0)
**Tested positive for smoking in CO sample at 12 weeks, n (%)**				

	Incentives		Usual care	
	Pre-Covid	Post-Covid	Pre-Covid	Post-Covid
Positive	4 (3.4)	18 (100))	NA	NA
Negative	114 (96.6)	0 (0)	NA	NA
Missing	0 (0)	0 (0)	NA	NA
**Tested positive for smoking in biochemical sample at late pregnancy, n (%)**				
Positive	13 (11.7)	6 (16.2)	12 (20.0)	1 (6.7)
Negative	96 (86.5)	30 (81.1)	45 (75.0)	13 (86.7)
Missing	2 (1.8)	1 (2.7)	3 (5.0)	1 (6.7)
**Tested positive for smoking in biochemical sample at 6 months postpartum, n (%)**				
Positive	7 (50.0)	16 (34.8)	3 (37.5)	12 (38.7)
Negative	7 (50.0)	30 (65.2)	5 (62.5)	19 (61.3)

Table 6a presents a comparison by treatment group of biochemically-verified smoking status (or CO-verified smoking status for the 4 week and 12 week time points) pre-Covid and post-Covid (excluding three participants who withdrew and requested data be removed).

	Incentives		Usual care	
	Pre-Covid	Post-Covid	Pre-Covid	Post-Covid
**4 weeks, n (%)**				
Non-smoker	151 (34.1)	11 (39.3)	56 (12.6)	6 (22.2)

	Incentives		Usual care	
	Pre-Covid	Post-Covid	Pre-Covid	Post-Covid
Smoker	133 (30.0)	7 (25.0)	121 (27.3)	8 (29.6)
Missing	159 (35.9)	10 (35.7)	266 (60.0)	13 (48.1)
**12 weeks, n (%)**				
Non-smoker	106 (77.4)	18 (72.0)	NA	NA
Smoker	17 (12.4)	4 (16.0)	NA	NA
Missing	14 (10.2)	3 (12.0)	NA	NA
**Late pregnancy, n (%)**				
Non-smoker	96 (26.8)	30 (26.5)	45 (12.6)	13 (11.5)
Smoker	220 (61.5)	66 (58.4)	288 (80.7)	85 (75.2)
Missing	42 (11.7)	17 (15.0)	24 (6.7)	15 (13.3)
**6 months post-partum, n (%)**				
Non-smoker	23 (6.4)	14 (12.4)	17 (4.8)	7 (6.2)
Smoker	255 (71.2)	85 (75.2)	259 (72.5)	95 (84.0)
Missing	80 (22.3)	14 (12.4)	81 (22.7)	11 (9.7)

Table 7a Comparison by treatment group of the number of participants who provided a positive biochemical sample (or CO sample for the 4 and 12 week time points), in the subset of participants who self-reported as non-smokers. The number of missing samples provides a measure of biochemical sample non-return rate in participants who reported as non-smokers (excluding three participants who withdrew and requested data be removed).

	Incentives		Usual care	
	Pre-Covid	Post-Covid	Pre-Covid	Post-Covid
**Tested positive for smoking in CO sample at 4 weeks, n (%)**				
Positive	10 (6.1)	0 (0)	2 (2.3)	0 (0)
Negative	151 (91.5)	11 (100)	56 (65.1)	6 (100)
Missing	4 (2.4)	0 (0)	28 (32.6)	0 (0)
**Tested positive for smoking in CO sample at 12 weeks, n (%)**				
Positive	4 (3.3)	0 (0)	NA	NA
Negative	114 (93.4)	18 (90.0)	NA	NA
Missing	4 (3.3)	2 (10.0)	NA	NA
**Tested positive for smoking in biochemical sample at late pregnancy, n (%)**				
Positive	13 (10.5)	6 (13.3)	12 (17.9)	1 (5.0)
Negative	96 (77.4)	30 (66.7)	45 (67.2)	13 (65.0)
Missing	15 (12.1)	9 (20.0)	10 (14.9)	6 (30.0)
**Tested positive for smoking in biochemical sample at 6 months postpartum, n (%)**				
Positive	7 (41.2)	16 (29.1)	3 (25.0)	12 (29.3)
Negative				

	Incentives		Usual care	
	Pre-Covid	Post-Covid	Pre-Covid	Post-Covid
Missing	7 (41.2)	30 (54.5)	5 (41.7)	19 (46.3)
	3 (17.6)	9 (16.4)	4 (33.3)	10 (24.4)

Appendix E: Study and data monitoring plan

STUDY & DATA MONITORING PLAN

The Smoking **C**essation in **P**regnancy **I**ncentives **T**rial (CPIT):

A Phase III Randomised Controlled Trial

Version 2.0

CHIEF INVESTIGATORS (CIs):

Professor David M Tappin<br>Professor of Clinical Trials for Children<br>University of Glasgow<br>Scottish Cot Death Trust<br>West Glasgow Ambulatory Care Hospital<br>Dalnair Street, Yorkhill<br>Glasgow, G3 8SJ	Professor Linda Bauld<br>Chair of Public Health<br>Usher Institute<br>The University of Edinburgh<br>Doorway 1<br>Old Medical School<br>Teviot Place<br>Edinburgh, EH9 4LA
**Phone:** 07784 304568	**Phone:** 0131 650 3213
**Email:** david.tappin@glasgow.ac.uk	**Email:** linda.bauld@ed.ac.uk

**SPONSOR:**<br>NHS Greater Glasgow and Clyde	**SPONSOR REPRESENTATIVE:**<br>Ms Emma McDonough<br>Clinical Research & Development Central Office<br>Ward 11 Dykebar Hospital<br>Grahamston Road<br>Paisley, PA2 7DE
	**Phone:** 0141 314 4011
	**Email:** emma.mcdonough@ggc.scot.nhs.uk

**SITE AND DATA MONITORING:**<br>York Trials Unit<br>ARRC Building, Lower Ground Floor<br>University of York<br>Heslington<br>York<br>YO10 5DD	**SITE MONITOR:**<br>Up to October 2019:<br>Helen Tilbrook<br>Research Fellow<br>York Trials Unit<br><br>From October 2019:<br>Dr Lyn Robinson-Smith<br>Research Fellow/Trial Manager<br>York Trials Unit
	**Phone:** 01904 321692
	**Email:** lynrobinsonsmith@york.ac.uk

REFERENCE NUMBERS

Sponsor Project Number: GN14OB206

ISRCTN: ISRCTN15236311 (date *registered* 13/10/2017)

IRAS Project ID: 227489

REC Ref: 17/WS/0173. West of Scotland REC 4

Trial web site: https://www.york.ac.uk/healthsciences/research/trials/research/trials/cpit-iii/

Contents

1.0 Introduction 4

2.0 Person(s) responsible for conducting trial monitoring 4

3.0 Risk assessment 4

4.0 Data quality assurance 4

5.0 Remote or on-site data monitoring 4

5.1 Timing and frequency of site-specific monitoring 5

5.2 Preparation prior to data monitoring event 5

5.3. Checks to be made during data monitoring events 5

5.4 Site-self data monitoring 6

6.0 Remote data quality assurance 6

6.1 Data exports data monitoring – purpose and frequency 6

6.1.1. Data summaries 6

6.1.2 Other specific data checks 8

6.1.2.1 Screening stages 8

6.1.2.2 Voucher fulfilment criteria 8

6.1.2.3 Checking for miscarriage variable 9

6.3 ECHO Managed Services monitoring 9

6.4 Changes to Data Collection and Monitoring Resulting from the COVID-19 Pandemic 9

Appendix 1 Overview of Data Items requiring Validation 10

Appendix 2 ISF Checklist 24

Appendix 3: Site Self-Monitoring Source Data 24

Appendix 4: Site Self-Monitoring Source Data during COVID-19 26

1.0 Introduction

The purpose of this document is to outline the monitoring requirements and data quality assurance for the CPIT III study.

2.0 Person(s) responsible for conducting trial monitoring

Monitoring and/or audits will be conducted by the trial co-ordinator and/or monitored from York Trials Unit (YTU), University of York (CPIT III protocol, section 14.2, v5.0 29th July 2020).

Remote data monitoring will also be conducted by the trial statistician at YTU. The study may also be subject to inspection by the funders and/or inspection and audit by the sponsor (CPIT III protocol, section 14.1, v5.0, 29th July 2020).

The CPIT Trial Administrator based within NHS Greater Glasgow & Clyde, will conduct or assist with monitoring.

Participating sites will be responsible for self-monitoring at specified time-points.

3.0 Risk assessment

A formal risk assessment has not been conducted by the sponsor as CPIT III is a non-CTIMP study and based on the pilot study design, intervention, participant recruitment and retention is considered to be minimal risk. The main risk is disclosure of identifiable data (CPIT III protocol, section 16.4, v5.0, 29th July 2020).

4.0 Data quality assurance

The study will be fully compliant with the Research Governance Framework. Audits of enrolment, participant retention and outcome assessment will be performed by a trial co-ordinator and/or monitored from YTU. Written reports will be produced for the Trial Steering Committee (TSC to take the role of the Data Management Committee) informing them of any corrective action that is required (CPIT III protocol, section 14.2, v5.0, 29th July 2020).

5.0 Remote or on-site data monitoring

Site-specific data monitoring includes that conducted remotely or on-site by the YTU data monitor.

The purpose of site data monitoring is to:-

- Verify research data to ensure accuracy and completeness from source documents (see Appendix 1).
- Assess protocol compliance and ensure trial procedures are being followed.
- Check use of current trial documents.
- Make checks around samples that are being collected (saliva, urine, breath test and blood). This will include checking a sample of specimens to ensure the information listed on specimen labels matches that recorded within the ECHO portal.

5.1 Timing and frequency of site-specific monitoring

Data monitoring (on-site or telephone) will be conducted at least once to capture each stage of data collection and on an 'as-needed' basis thereafter and dependent on factors such as:

- Recruitment rates.
- If there are any concerns reported by sites which may indicate the integrity of randomisation or data collection may be compromised.
- The amount of data identified as inaccurate/missing during the first data monitoring event.
- If a stage of data collection e.g. recruitment, 4 week stage, 12 week stage, late pregnancy stage (primary outcome) or 6-month postpartum stage was not captured during the first data monitoring event.

Where an on-site visit is not deemed necessary (see section below), site monitoring will be completed remotely over the phone between YTU and each participating site whereby data noted on relevant data sources can be verbally confirmed and checked against ECHO database.

5.2 Preparation prior to data monitoring event

All sites will be requested to send the following documents to the YTU trial monitor in advance of the site monitoring event:-

- Delegation log
- Screening log
- Electronic Investigator Site File (ISF)
- CO Validation Collection Form (where used)

The participant data to be checked during the data monitoring event will be randomly selected by the trial statistician at YTU. Prior to data monitoring, YTU will provide sites with a list of (randomly) selected participant IDs where applicable and the corresponding source data required for verification.

5.3. Checks to be made during data monitoring events

During data monitoring events, the following checks will be made:

- **Source data verification** (SDV): Where possible trial data held to be checked against a proportion of source data (includes: date of birth, CO results, adverse event data). For full details see Appendix 1. NOTE: Adverse event data will be entered directly in to the database portal held by ECHO. The CI will review SAEs on the database portal. Data will not be entered on to hard copy forms.
- **Screening log**: Where possible to check a proportion of source data against screening log data.
- **Investigator Site file**: Check that current documents and procedures are being used; check CVs and GCP certificates are on file; check that delegation log is on file and is complete.
- Check that all documentation and data is complete, accurate and legible.
- Checks around samples being collected (saliva, CO, urine and blood).

A 'Monitoring Summary Report' will be completed following the monitoring event (using the template provided by York Foundation Trust R&D Unit Form R&D/F69) and where necessary

an 'Action List' will be completed (using a template provided by York Foundation Trust R&D Unit Form RDF66 Version 5 (1) Action List).

5.4 Site-self data monitoring
Site-self monitoring refers to the site itself checking that source data matches what is recorded in the ECHO Managed Services (ECHO) database records and reporting back to the YTU data monitor.

Self-monitoring may also be completed to help ensure that the study is being run and documented to a standard that would be acceptable to being audited by the local site R&D Department. We would propose the YTU data monitor instructs that sites complete the self-monitoring ISF checklist (Appendix 2) yearly and self-checking of source data items (Appendix 3) at different stages throughout the trial to ensure all data collection points have been checked at least one. Sites are to provide self-monitoring feedback to the YTU data monitor.

6.0 Remote data quality assurance

6.1 Data exports data monitoring – purpose and frequency
All trial data (screening, consent, follow-up and adverse event data) will be available regularly (approximately weekly) for download from ECHO. Two datasets will be made available by ECHO to York Trials Unit; one will be anonymised (i.e. names, postal addresses, email addresses, telephone numbers and GP contact details will be removed) for use by the trial statistician and downloaded weekly and the other will be non-anonymised for use by the trial manager at YTU) and downloaded on an as-needed basis. Initially, data should be checked monthly. Subsequently, data will be checked for reporting to Trial Management Group meetings and Trial Steering Group meetings.

Electronic screening logs are completed at the recruiting sites. Sites should send electronic Screening Logs on the first working day of the month to lyn.robinson-smith@york.ac.uk (previously helen.tilbrook@york.ac.uk) and Alex Mitchell, alex.mitchell@york.ac.uk. The data should be sent via a secure data transfer method.

The purpose of remote monitoring is to:
- Ensure that data is accurate and complete.
- Assess protocol compliance/trial procedures are being followed.

6.1.1. Data summaries
Summary reports required for data monitoring and data quality assurance will be generated by the YTU from the data provided by ECHO. The majority of the reports required are listed in Table 1. Many of the summary reports listed below will need to be produced by YTU from ECHO's data export.

**Data summaries**	**Trial Total**	**By site**	**Random. group**	**Checks/Purpose**
• Total number of participants consented<br>• Total number of participants randomised	✓	✓	✓	Check against target recruitment for the trial and for sites.

Data summaries	Trial Total	By site	Random. group	Checks/Purpose
Recruitment rate:<br>Total number of participants randomised in each month	✓	✓	✓	Check against target recruitment for the trial and for sites.
Number and proportion of eligible patients recruited (= no. randomised/screened as eligible[1])	✓	✓	n/a	Eligibility rate
Number and proportion of eligible patients who consented (= no. consent/screened as eligible[1])	✓	✓	n/a	Consent rate
Number and proportion of eligible patients who refused consent (= no. refused consent/screened as eligible[1])	✓	✓	n/a	Refused consent rate
Follow-up rate (4 week, 12 week, late pregnancy and 6 months post-partum):<br>Number and proportion of completed follow-ups (=completed follow-up /follow-up due).	✓	✓	✓	Follow-up rate
Number and proportion of participants withdrawn from the trial grouped by time of last follow-up (i.e. (1) Withdrawn before the end of pregnancy, and (2) withdrawn after pregnancy and before end of final follow-up (=no withdrawn from trial/total randomised)	✓	✓	✓	Withdrawal rate
Number and proportion of participants lost to follow-up grouped 4 week, 12 week, late pregnancy and post-partum	✓	✓	✓	Lost to follow-up
Number and proportion of participants who have withdrawn their data (i.e. do not want their data to be used) (=no. withdrawn their data/total randomised)	✓	✓	✓	No. who have withdrawn data
Adverse Event data (to include participant ID, site ID, description of event, date of onset, date of resolution, causal assessment, severity assessment (i.e. serious or non-serious).	✓	✓	✓	For adverse event reporting to TMG & TSC

[1]To include participants that have withdrawn from trial or treatment or have been lost to follow-up. NOTE regarding screening Logs. Site D and Site C sites – All patients referred to the SSS are included on the screening log.

6.1.2 Other specific data checks

6.1.2.1 Screening stages

Check that ineligible patients at the screening stage have not been uploaded to ECHO database.

- Stop Smoking Service (SSS) referrals are typically received from the relevant maternity services - occasionally referrals come from other sources e.g. GP, self-referral.
- SSS make contact with individuals to discuss the smoking cessation support available to them.
- SSS staff will assess individuals against all CPIT eligibility criteria, adding a checklist label to the paper referral form (where this exists) and ticking off criteria as they are met.
- Maternity referral forms may specify the number of weeks/days gestation at time of referral, current age AND date of birth, and these details are confirmed with the individual. Where these data items are not provided on the referral form SSS staff will need to calculate these.
- Level of spoken English will be assessed by SSS during first routine contact.
- If the individual is eligible to participate in the study, SSS/Research staff will discuss the study and seek relevant permissions to pass details to the study team.
- CPIT Team will then transcribe individuals' data from paper referral form to Excel spreadsheet (Import Workbook).
- This provides a second check of all eligibility criteria before individuals are imported to the CPIT database.
- All individuals – eligible (those who are eligible are assigned a CPIT URN) and ineligible – are added to the Screening Log
- Eligibility criteria are further checked electronically during the import process and alerts generated to the trial team to investigate as appropriate.
- During the consent call Expected Delivery Date (EDD) is verified with the participant as a further check on gestation.
- On receipt of the screening logs the trial statistician makes further checks.

6.1.2.2 Voucher fulfilment criteria

The procedure for checking that only participants who meet the fulfilment criteria are sent vouchers is below (see notes below tables in Appendix 1 for fulfilment criteria. Criteria are also specified in the Business Requirement Specification document).

- After vouchers are dispatched, ECHO update the CPIT Portal to add Royal Mail tracking references to each participant's Event Log
- This information is contained within the weekly Glasgow CPIT data extract, allowing CPIT study team to export and collate tracking information for all vouchers sent by Latchem Direct
- Voucher Tracking spreadsheet is updated to add: URN, Voucher Type, Voucher Number, Voucher Value, Voucher Date Sent, Tracking Reference Number

- CPIT Administrator checks each tracking reference on Royal Mail Track and Trace website to confirm voucher has been received
- Voucher Tracking spreadsheet is updated to include: Delivery Confirmation Details (date, time and name of signature), Patient Name (flagged in bold if different to signature), Delivery Notes (e.g. Royal Mail Delivery Office)
- If delivery has been unsuccessful, an SMS will be sent to participant to advise their voucher is awaiting collection.
- Where necessary, further contact with participant is made to ensure delivery of vouchers and voucher tracking spreadsheet updated accordingly.

6.1.2.3 Checking for miscarriage variable

Check Miscarriage variable [Entry TRUE/FALSE] coincides with Miscarriage-date variable, i.e. Where Miscarriage variable is True, there is a miscarriage date and vice versa and has a recorded SAE.

6.3 ECHO Managed Services monitoring

ECHO Managed Services will undergo remote monitoring of the consent process by the qualitative data monitor based at University of Stirling. Here a proportion of audio-recorded consent calls will be listened to as part of the process evaluation.

It is proposed that a sample of four consent call recordings from each of 3 call handlers employed by ECHO who had carried out >10 consent calls (involving 2 calls to women in Site C and 2 calls to women in Site D for each individual) will be checked. Overall this would come to 12 call checks. The checks will involve ensuring that participants have answered "yes" to mandatory consent questions 1-5 & 15. If any issues are identified during this review, ECHO will be informed immediately to ensure appropriate re-training of call handlers and the qualitative trial monitor may choose to conduct a further check at a later time point, if deemed necessary.

Operators taking consent: GCP certificates to be obtained for staff employed by ECHO Managed Services who are taking patient consent.

6.4 Changes to Data Collection and Monitoring in response to the COVID-19 Pandemic

As a result of the COVID-19 pandemic, the final phase of data collection was completed by the central research team, in most cases. A detailed description of the changes to the trial's data collection due to COVID-19 can be found in the revised protocol: v5.0 29th July 2020.

As a consequence of COVID-19, researchers were not permitted to conduct any further site visits for data monitoring purposes. To ensure some data monitoring checks were completed, site-self data monitoring checks were refined to focus on variables that were key to primary and secondary outcomes (see Appendix 4). This was to ensure not to over-burden NHS research staff who were redeployed for COVID-duties.

Appendix 1 Overview of Data Items requiring Validation

The tables summarise key data items collected in the CPIT Portal that require to be checked to ensure a high level of data quality and integrity in the final dataset available for analysis.

The Tables within this Appendix include a column ‘Manual Check Required’. This indicates where a variable requires a manual check to be carried out over and above the automated electronic check conducted based on the data extract provided by ECHO. For most variables no manual checks are required as the data will be input directly into the CPIT Portal, for example, whilst the patient is on the phone with the call handler at ECHO.

The availability of source data is likely to vary from site to site, as source data may be passed from different services e.g. from the midwifery service to the Stop Smoking Service. In these instances, data monitoring will start from the point that eligibility screening took place i.e. where the referral information was received.

Table 1: Key Data Items requiring Validation: Eligibility, Consent and SSS Engagement

Data Item	Data Item Related to								
	Demo-graphics	Eligibility Criteria	Consent Criteria	Primary Outcome	2ndary Outcome	Voucher Dispatch	Manual Check Required<br><br>Checks to be made during monitoring	Source data if applicable	Checks made by YTU via data coding
**% of Records to be Checked**	**10%**	**100%**	**100%**	**100%**	**10%**	**100%**	**Y/N**		
**Eligibility/Import Stage**									
Site		√					N		
Maternal height	√						Y	NHS record (BADGERNET in NHSL and NIMATS in BHSCT).<br><br>If not available on referral form it is collected during consent call by self-report. If the latter, no check required.	
Maternal weight	√						Y	NHS record (BADGERNET in NHSL and NIMATS in BHSCT).<br><br>If not available on referral form it is collected during consent call by	

								self-report. If the latter, no check required.	
Initial CO Result	√						Y	SSS or NHS record (BADGERNET in NHSL and NIMATS in BHSCT).<br><br>Not always available on referral form.	
AN Appointment Date		√					Y	Screening log<br><br>SSS or NHS record (BADGERNET in NHSL and NIMATS in BHSCT).	
**Self-Reported Smoker**		√					Y	Screening log<br><br>SSS or NHS record (BADGERNET in NHSL and NIMATS in BHSCT).	
**Date of Birth**		√					Y	NHS record	Checked at consent and TM informed of errors as can't be updated by ECHO.

**Estimated Delivery Date (<24 wks)**		√					Y	Import file matches NHS record	Can be amended by ECHO during consent (where incorrect).
**English Speaking**		√					N	Site	Checked by ECHO
**Consent Call Stage**									
Date of Consent (ConsentCallStage_Stage_Closed_At)			√				N		
All consent questions answered			√				N		
All mandatory consent questions answered 'YES'			√				N		√
Age started smoking	√						N		
Fagerstrom: First Cigarette	√						N		√
Fagerstrom: Forbidden Smoking	√						N		√
Fagerstrom: Which Cigarette	√						N		√
Fagerstrom: Cigarettes per day	√						N		√
Fagerstrom: Smoke Morning More	√						N		√
Fagerstrom: Smoke If Ill	√						N		√
Partner Smoke	√						N		√
Fagerstrom Score	√						N		√
EQ-5D: Mobility					√		N		√
EQ-5D: Self care					√		N		√
EQ-5D: Usual activities					√		N		√
EQ-5D: Pain discomfort					√		N		√
EQ-5D: Anxiety & Depression					√		N		√
EQ-5D: VAS Score					√		N		√
Household income	√				√		N		
Use NRT	√				√		N		
Use Ecig	√				√		N		
Ethnicity	√						N		
Ethnicity Group	√						N		
Ethnicity Other	√						N		
Randomisation ID		√					N		
Randomisation (Assigned)		√					N		

Randomisation date (Assigned_at)		√					N		
**SSS Engagement Stage**									
Skipped At (i.e. engagement)					√	√	N	SSS – Client record/SSS database	
Appointment Date					√		Y	As above	
Attended[2]					√	√	Y [1]	As above	
CO Result available					√		N		
CO Result					√		Y	As above	
Quit Date Set					√	√	N	As above	
Quit Date[2]					√	√	Y [1]	As above	

[1] 100% Intervention Group and 100% Control Group

[2]Intervention group: If appointment attended and a quit date is set, check that £50 voucher has been sent. Control group: Check that a voucher has not been sent.

Table 2: Key Data Items requiring Validation: 4 Week Stage

	Data Item Related to				
**Data Item**	**Primary Outcome**	**2ndary Outcome**	**Voucher Dispatch**	**Manual Check Required Checks to be made during - monitoring**	**Source data if applicable**
**% of Records to be Checked**	**100%**	**10%**	**100%**	**Y/N**	
**4 Week Stage (Int & control)**					
Skipped stage date/reason		√	√	Y	SSS Client record/database
Appointment Date		√		Y	SSS Client record/database/ CPIT 4-week CO Form
Attended		√	√	Y	SSS Client record/database/ CPIT 4-week CO Form
CO Result Available		√	√	Y	SSS Client record/database/ /CPIT 4-week CO Form
CO Result[1,2]		√	√	Y	SSS Client record/database/ /CPIT 4-week CO Form
CO Result Date		√	√	Y	SSS Client record/database/ /CPIT 4-week CO Form
Smoked in last 2 weeks[1]		√	√	Y	SSS Client record/database/ /CPIT 4-week CO Form

[1] Intervention group: If self-report as quit **AND** CO result is <4ppm (Site C site) or <5ppm (Site D, Site A, Site B, Site E, Site G sites) or <6ppm (Site F site), check that £50 has been sent. Control group: Check that a voucher has **NOT** been sent. This is the self-report as quit variable in the export template. The Russell Standard question defines a self-reported quitter as someone who has not smoked in the last 2 weeks of the last 4 weeks i.e. they are allowed 2 weeks grace since they set their quit date

[2]Data should be checked for 10% complete for intervention group (Note: CO result will only be available where CO result available = Y). Data may be missing for the control group as will only be available if taken as part of routine care.

Table 3: Key Data Items requiring Validation: 12 Week Stage

	Data Item Related to				
**Data Item**	**Primary Outcome**	**2ndary Outcome**	**Voucher Dispatch**	**Manual Check Required Checks to be made during monitoring**	**Source data if available**
**% of Records to be Checked**	**100%**	**10%**	**100%**	**Y/N**	
**12 Week Stage (Only Int & Cont participants who self-report as quit at 4 wks)**					
Skipped stage date/reason		√	√	Y	SSS Client record/database
Appointment Date		√		Y	SSS – Client record/SSS database/ CPIT 12-week CO Form
CO Result Available		√	√	Y	SSS/CPIT 12-week CO Form
CO Result[1, 2]		√	√	Y	SSS/CPIT 12-week CO Form
CO Result Date		√	√	Y	SSS/CPIT 12-week CO Form
Smoked Question[2]		√	√	Y	SSS/CPIT 12-week CO Form

[1]Data should be 10% complete for **intervention participants** who self-report as quit at 12 weeks **IF** participants were self-reported quit at 4 weeks **AND** CO result was within the permitted level (see below). Data may be missing for the control group as will only be available if taken as part of routine care.

[2] **Intervention group**: If self-report as quit and CO result is <4ppm (Site C site) or <5ppm (Site D, Site A, Site B, Site E, Site G sites) or <6ppm (Site F site) **AND** self-reported as quit at 4 weeks **AND** 4 week CO result was within the permitted level, check that £100 voucher has been sent (If all criteria were not met then a voucher should not be sent). **Control group**: Check that a voucher has **NOT** been sent.

Table 4: Key Data Items requiring Validation: Late pregnancy stage (primary outcome)

	Data Item Related to				
**Data Item**	**Primary Outcome**	**2ndary Outcome**	**Voucher Dispatch**	**Manual Check Required Checks to be made during monitoring**	**Source data if available**
**% of Records to be Checked**	**100%**	**10%**	**100%**	**Y/N**	
**34-38 Week Call Stage (Int & Cont)**					
Skipped Stage Date/Reason	√			N	
a) Smoked in last 8 weeks?[1,2,3]	√		√	N	Captured during call by RN or ECHO call Handler and entered directly into database
b) If yes, Smoked more than 5?[1,2,3]	√		√	N	As above
**Self Reported Quit (Int & Cont)**					
Skipped Stage Date/Reason	√		√	Y	
CO Result Available	√		√	Y	SSS Client Record/Database, CPIT III LP CRF
CO Result[2,3]	√		√	Y	SSS Client Record/Database, CPIT III LP CRF
CO Result Date	√		√	Y	SSS Client Record/Database, CPIT III LP CRF
Urine Collected[3]	√		√	Y	CPIT III LP CRF, Lab Log
Urine Sample Number	√			Y	CPIT III LP CRF, Lab Log
Urine Sample Date	√			Y	CPIT III LP CRF, Lab Log

Urine Cotinine Level	√			Y	ABS Labs Report
Urine Anabasine Level[3]	√			Y	ABS Labs Report
Saliva Collected[3]	√		√	Y	CPIT III LP CRF, Lab Log
Saliva Sample Number	√			Y	CPIT III LP CRF, Lab Log
Saliva Sample Date	√			Y	CPIT III LP CRF, Lab Log
Saliva Cotinine Level	√			Y	ABS Labs Report
Saliva Anabasine Level[3]	√			Y	ABS Labs Report
Blood Collected[3]	√			Y	Lab Log
Blood Sample Number	√			Y	Lab Log
Blood Sample Date	√			Y	Lab Log
Blood Cotinine Level	√			Y	ABS Labs Report
Blood Anabasine Level	√			Y	ABS Labs Report
Currently using NRT	√			Y	SSS Client Record/Database, CPIT III LP CRF
Currently using e-cig	√			Y	SSS Client Record/Database, CPIT III LP CRF
EQ-5D: Mobility		√		N	
EQ-5D: Self care		√		N	
EQ-5D: Usual activities		√		N	
EQ-5D: Pain discomfort		√		N	
EQ-5D: Anxiety & Depression		√		N	
EQ-5D: VAS Score				N	

[1]If the participant answers “No” to question ‘a’ **OR** No” to question ‘b’, the participant will have reported as a ‘non-smoker’ for the primary outcome (see trial protocol, section 10.2.4., V4.0 11 March 2020).

[2]**Intervention group**: If self-report as quit (i.e. non-smoker (see [1]) **AND** CO result is <4ppm (Site C site) or <5ppm (Site D, Site A, Site B, Site E, Site G sites) or <6ppm (Site F site) **AND** provide **at least one** of the biochemical samples, check that £200 voucher has been sent. **Control group**: Check that a £200 voucher has **NOT** been sent.

[3]**Intervention and control group**: If provide an answer to 'a' AND 'b' as appropriate (**AND** provide if quit **at least one** of the biochemical samples), check that a £50 voucher has been sent.

Table 5: Key Data Items requiring Validation: Six months post-partum (secondary outcomes)

	Data Item Related to				
**Data Item**	**Primary Outcome**	**2ndary Outcome**	**Voucher Dispatch**	**Manual Check Required**<br><br>**Checks to be made during monitoring**	**Source data if available**
**% of Records to be Checked**	**100%**	**10%**	**100%**	**Y/N**	
**Post-partum Call Stage (Int & Cont)**					
Skipped Stage Date/Reason		√	√	N	
a) Smoked in last 8 weeks?[1]		√	√	N	Captured during call by RN or ECHO call Handler and entered directly into database
b) If yes, Smoked lmore than 5?[1]		√	√	N	As above
c) Smoked since baby born		√	√	N	As above
d) If Yes, Smoked more than 5?		√	√	N	As above
**Self Reported Quit (Int & Cont)**					
Skipped Stage Date/Reason		√		Y	
CO Result Available		√		Y	SSS Client Record/Database, CPIT III PP CRF

CO Result[2,3]		√		Y	SSS Client Record/Database, CPIT III PP CRF
CO Result Date		√		Y	SSS Client Record/Database, CPIT III PP CRF
Urine Collected[2]		√		Y	CPIT III PP CRF, Lab Log
Urine Sample Number		√		Y	CPIT III PP CRF, Lab Log
Urine Sample Date		√		Y	CPIT III PP CRF, Lab Log
Urine Cotinine Level		√		Y	ABS Labs Report
Urine Anabasine Level		√		Y	ABS Labs Report
Saliva Collected[2]		√		Y	CPIT III PP CRF, Lab Log
Saliva Sample Number		√		Y	CPIT III PP CRF, Lab Log
Saliva Sample Date		√		Y	CPIT III PP CRF, Lab Log
Saliva Cotinine Level		√		Y	ABS Labs Report
Saliva Anabasine Level		√		Y	ABS Labs Report
Currently using NRT		√		Y	SSS Client Record/Database, CPIT III PP CRF
Currently using e-cig		√		Y	SSS Client Record/Database, CPIT III PP CRF
EQ-5D: Mobility		√		N	
EQ-5D: Self care		√		N	
EQ-5D: Usual activities		√		N	
EQ-5D: Pain discomfort		√		N	
EQ-5D: Anxiety & Depression		√		N	
EQ-5D: VAS Score		√		N	

[1] *(Questions/variables to be determined in Phase II development).*

[1] If the participant answers "No" to question 'a' **OR** "No" to question 'b', the participant will have reported as a 'non-smoker' for the primary outcome (see trial protocol, section 10.2.4., V4.0 11 March 2020).

[12] **Intervention and control group**: If provide an answer to 'a' AND 'b' as appropriate (**AND** provide if quit **at least one** of the biochemical samples), check that a £25 voucher has been sent.

Table 6: Key Data Items requiring Validation: End of pregnancy

	Data Item Related to				
**Data Item**	**Demo-graphics**	**Primary Outcome**	**2ndary Outcome**	**Manual Check Required Checks to be made during monitoring**	**Source data if available**
**% of Records to be Checked**	**10%**	**100%**	**10%**	**Y/N**	
**Gestation Stage**					
Birth Weight			√	Y	NHS record
Delivery Date			√	Y	NHS record
Parity[1]	√			Y	NHS record[1]
Still Birth			√	Y	NHS record

[1] Site C site – Parity is not updated in source data (NIMATS) by the NHS staff until women present with a new pregnancy. Therefore research team will use birth data, miscarriage data, etc to obtain parity.

Table 7: Key Data Items requiring Validation: Adverse events

All adverse event data will be entered directly into the trial database at site.

	Data Item Related to		

**Data Item**	**Demo-graphics**	**Primary Outcome**	**2ndary Outcome**	**Manual Check Required (Not applicable – no source data)**	**Source data if available**
**% of Records to be Checked**	**10%**	**100%**	**10%**		
**All time points**					
Date of onset			√		
Description of AE event			√		
Causal (Relatedness)			√		
Serious			√		
Expected			√		
PI Informed*			√		
AE reviewed			√		
AE resolution			√		

*Only required if SAE

Table 8: Key Data Items requiring Validation: Others

	**Data Item Related to**				
**Data Item**	**Demographics**	**Primary Outcome**	**2ndary Outcome**	**Manual Check Required**	**Source data if available**

% of Records to be Checked		100%	10%	Y/N	
**All time points**					
Withdrawn		√		N	
Loss to follow-up		√		N	

Appendix 2 ISF Checklist

Commented [RL1]: TO BE ADDED once confirmed.

Appendix 3: Site Self-Monitoring Source Data

York Trials Unit will provide a list of randomly selected participant IDs for records to be checked.

Demographics

10% of all records (control/intervention groups) in portal to be checked against source data:

- Maternal Height (if not recorded at first appointment, it is recorded directly by ECHO)
- Maternal Weight (if not recorded at first appointment, it is recorded directly by ECHO)
- Initial CO results (taken by the midwifery service around the time of referral)

Eligibility

100% of all records (control/intervention groups) in portal to be checked against source data:

- AN Appointment Date/Referral date to SSS – eligibility confirmed
- Self-reported smoker
- Patient's date of birth

100% of records in import file matches source data:

- Estimated delivery date – Check that import file matches source data

SSS Engagement

100% of intervention & 10% of control groups records in portal to be checked against source data:

- Appointment dates with SSS advisor
- Attended
- Quit date
- CO result

4 & 12 week – Intervention group only

10% of records in portal to be checked against source data – e.g. logs, forms:

- CO result
- CO result date
- Smoked in the last 2 weeks

Primary outcome and six months post-partum

100% of records who have reported to have stopped smoking in portal to be checked against source data – e.g. logs, forms:

- Self-reported smoking status
- CO result
- CO result date
- Sample results – cotinine/anabasine validated result, from lab. – urine or saliva

10% of sample labels (saliva, urine, blood) to be checked to ensure the information listed on specimen labels matches that recorded within the ECHO portal.

<u>End of pregnancy</u>

10% of all records (intervention/control group) in portal to be checked against source data.

- Birth weight
- Delivery date
- Parity
- Still birth

Appendix 4: Site Self-Monitoring Source Data during COVID-19

Due to the COVID-19 pandemic, starting in March 2020, data monitoring plans were streamlined to have focus on checking variables that were key to the trial's primary and secondary outcomes and to reduce the workload on NHS trial staff who were deployed to COVID-specific duties.

York Trials Unit provided each study site with a list of randomly selected participant IDs with populated data fields for records to be checked.

CHECKS NOT PLANNED TO BE COMPLETED DURING COVID-19:

Demographics: 10% of all records (control/intervention groups) in portal to be checked against source data:

- Maternal Height (if not recorded at first appointment, it is recorded directly by ECHO)
- Maternal Weight (if not recorded at first appointment, it is recorded directly by ECHO)
- Initial CO results (taken by the midwifery service around the time of referral)

Eligibility: 100% of all records (control/intervention groups) in portal to be checked against source data:

- AN Appointment Date/Referral date to SSS – eligibility confirmed
- Self-reported smoker
- Patient's date of birth
- 100% of records in import file matches source data:
- Estimated delivery date – Check that import file matches source data

12 week – intervention group only: 10% of records in portal to be checked against source data – e.g. logs, forms:

- CO results
- CO result date
- Smoked in last 2 weeks

CHECKS PLANNED TO BE COMPLETED DURING COVID-19:

SSS Engagement: 100% of intervention & 10% of control groups records in portal to be checked against source data:

- Appointment dates with SSS advisor
- Attended
- Quit date
- CO result

4 week – Intervention group only: 10% of records in portal to be checked against source data – e.g. logs, forms:

- CO results
- CO result date
- Smoked in last 2 weeks

Primary outcome and six months post-partum: 100% of records who have reported to have stopped smoking in portal to be checked against source data – e.g. logs, forms

- Self-reported smoking status
- CO result
- CO result date
- Sample results – cotinine/anabasine validated result, from lab. – urine or salivia

10% of sample labels (saliva, urine, blood) to be checked to ensure the information listed on specimen labels matches that recorded within the ECHO portal.

End of pregnancy: 10% of all records (control/intervention groups) in portal to be checked against source data.

- Birth weight
- Delivery date
- Parity
- Still birth

Appendix F: Additional analysis methods

Meta-analysis with CPIT II

Random effects meta-analysis using the restricted maximum likelihood method was carried out using the meta command in Stata version 17.0. The pooled risk ratio was reported alongside the I^2 statistic.

CACE analysis of birthweight

CACE analysis was carried out using instrumental variable regression, via the two-stage least squares approach. The endogenous variable of full compliance was defined as whether the participant was found to be a biochemically verified non-smoker at late pregnancy, with the instrumental variable being random group allocation. The exogenous variables were age, height and weight of the mother at booking, years of smoking, income status (as measured by the Index of Multiple Deprivation), level of smoking (as measured by the Fagerström score), whether the engagement data was collected before the 16th of March 2020, and centre. Centre was included as a fixed effect as the ivregress command does not allow for random effects.

Appendix G

NRT and E-cigarette use gathered at the primary outcome point in late pregnancy and 6-month post-partum

NRT use at late pregnancy, presented by group.

	Incentives (n=472)	Control (n=472)
**Currently using NRT, n (%)**		
**Number with data**	**373 (79.0)**	**355 (75.2)**
Yes	66 (17.4)	70 (19.7)
No	307 (82.3)	285 (80.3)
**Used NRT during pregnancy, n (%)**		
**Number with data**	**371 (78.6)**	**355 (75.2)**
Yes	234 (63.1)	226 (63.7)
No	137 (36.9)	129 (36.3)
**Frequency of NRT use, n (% of those who used during pregnancy)**		
**Number with data**	**231 (98.7)**	**222 (98.2)**
Daily or almost daily	171 (74.0)	173 (77.9)
Less than daily, but at least once a week	30 (13.0)	26 (11.7)
Less than weekly, but at least once a month	10 (4.3)	4 (1.8)
Less than monthly	20 (8.7)	19 (8.6)
**Duration of NRT use, n (% of those who used during pregnancy)**		
**Number with data**	**231 (98.7)**	**222 (98.2)**
One month	136 (58.6)	112 (50.5)
Two months	33 (14.2)	44 (19.8)

Three months	27 (11.6)	33 (14.9)
More than three months	35 (15.1)	33 (14.9)
**NRT obtained via, n (% of those who used during pregnancy)**		
**Number with data**	**233 (99.6)**	**223 (98.7)**
Dispensed via NHS, SSS, GP or pharmacy	209 (89.7)	194 (87.0)
Purchased	18 (7.7)	21 (9.4)
Both	6 (2.6)	8 (3.6)

NRT use at 6-months postpartum, presented by group.

	**Incentives (n=472)**	**Control (n=472)**
**Currently using NRT, n (%)**		
**Number with data**	**242 (51.3)**	**239 (50.6)**
Yes	19 (7.9)	37 (15.5)
No	223 (92.1)	202 (42.8)
**Used NRT since pregnancy, n (%)**		
**Number with data**	**240 (50.8)**	**237 (50.2)**
Yes	64 (26.7)	85 (35.9)
No	176 (73.3)	152 (64.1)
**Frequency of NRT use, n (% of those who used since pregnancy)**		
**Number with data**	**64 (100)**	**85 (100)**
Daily or almost daily	44 (68.8)	71 (83.5)
Less than daily, but at	10 (15.6)	11 (12.9)

least once a week		
Less than weekly, but at least once a month	4 (6.3)	1 (1.2)
Less than monthly	5 (7.8)	1 (1.2)
**Duration of NRT use, n (% of those who used since pregnancy)**		
**Number with data**	**63 (98.4)**	**84 (98.8)**
One month	28 (44.4)	41 (48.8)
Two months	14 (22.2)	10 (11.9)
Three months	3 (4.8)	9 (10.7)
More than three months	18 (28.6)	24 (28.6)
**NRT obtained via, n (% of those who used since pregnancy)**		
**Number with data**	**64 (100)**	**85 (100)**
Dispensed via NHS, SSS, GP or pharmacy	49 (76.6)	67 (78.8)
Purchased	11 (17.2)	17 (20.0)
Both	4 (6.3)	1 (1.2)

E-cigarette use at late pregnancy, presented by group.

	**Incentives (n=472)**	**Control (n=472)**
**Currently using e-cigarettes, n (%)**		
**Number with data**	**270 (57.2)**	**249 (52.8)**
Yes	41 (15.2)	35 (14.1)
No	229 (84.8)	214 (85.9)

**Used e-cigarettes during pregnancy, n (%)**		
**Number with data**	**268 (56.8)**	**248 (52.5)**
Yes	87 (32.5)	89 (35.9)
No	181 (67.5)	159 (64.1)

E-cigarette use at 6-months postpartum, presented by group.

	**Incentives (n=472)**	**Control (n=472)**
**Currently using e-cigarettes, n (%)**		
**Number with data**	**244 (51.7)**	**240 (50.8)**
Yes	44 (18.0)	48 (20.0)
No	200 (82.0)	80.0
**Used e-cigarettes since pregnancy, n (%)**		
**Number with data**	**239 (50.6)**	**238 (50.4)**
Yes	78 (32.6)	96 (40.3)
No	161 (67.4)	142 (59.7)

Appendix H: Pattern mixture model

Pattern mixture model

Figure 1 is a graphical illustration of the results of the pattern mixture model.

Figure 1: Illustration of results of missing data sensitivity analysis using the pattern mixture model approach. The graph demonstrates how the adjusted odds ratio of being a biochemically-verified non-smoker varies for varying values of the informative missingness odds ratio in incentives only, both arms and control only i.e. varying amounts of deviation from the missing at random assumption.

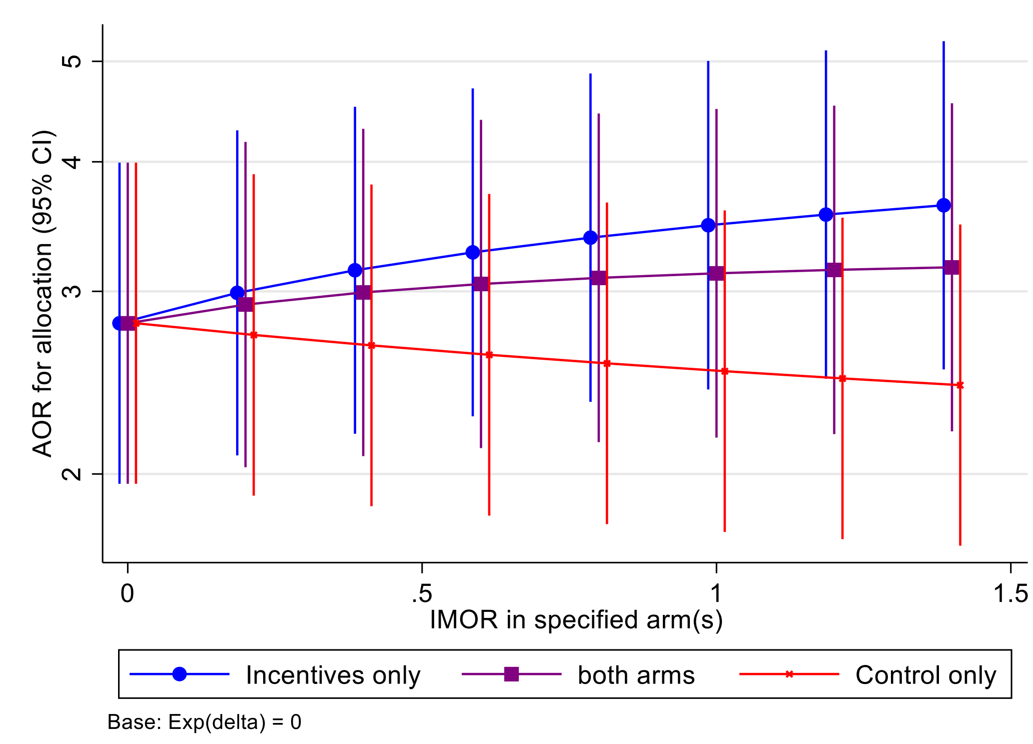

Supplement: Supplementary file 1 — Web appendix: Supplementary material [file tapd071522.ww.pdf]
